# Design, Synthesis, Molecular Docking, and Evaluation Antioxidant and Antimicrobial Activities for Novel 3-phenylimidazolidin-4-one and 2-aminothiazol-4-one Derivatives

**DOI:** 10.3390/molecules27030767

**Published:** 2022-01-24

**Authors:** Wesam S. Shehab, Maged Abdelaziz, Nourhan Kh. R. Elhoseni, Mohamed G. Assy, Magda H. Abdellattif, Eman O. Hamed

**Affiliations:** 1 Department of Chemistry, Faculty of Science, Zagazig University, Zagazig 44519, Egypt; mrmagedraof2017@gmail.com (M.A.); nourhankhairy123@gmail.com (N.K.R.E.); profmassy@yahoo.com (M.G.A.); dremanomar54@gmail.com (E.O.H.); 2 Department of Chemistry, College of Science, Taif University, P.O. Box 11099, Taif 21944, Saudi Arabia; m.hasan@tu.edu.sa

**Keywords:** imidazolothiazole, imidazotriazine, azabene, antioxidant activity, ABTS assay, antimicrobial agents, molecular docking

## Abstract

On our way to discovering and developing compounds that have an antioxidant impact compared to ascorbic acid and other biological activities, we designed, synthesized, and evaluated a new series of heterocyclic moieties drugs (**1**–**11**) as antioxidants and antimicrobial agents. As starting moieties, these new candidates were derived from two promising heterocyclic compounds, imidazoldin-4-one and thiazol-4-one. Firstly, diphenylimidazol **1** was obtained because of the cyclo condensation one-pot ternary reaction of urea, aniline, and chloroacetic acid under thermal conditions. Out of this starting compound, we could design and create new vital rings such as purine and triazine as in compounds **5** and **6**, respectively. Secondly, the start thiazole derivative **7** was obtained from the intermolecular cyclization of thiourea, chloroacetic acid, *p*-nitobezaldehyde in the presence of sodium acetate. We synthesized various derivatives from this second starting compound **7** by being subjected to different reagents such as aniline, phenylenediamine, phenylhydrazine, and barbituric acid to yield **8**, **9**, **10**, and **11**, respectively. Using ascorbic acid as the standard compound, the pharmacological testing for antioxidant activity assessment of the produced derivatives was evaluated against ABTS (2,20-azinobis (3-ethylbenzothiazoline-6-sulfonic acid). Candidate **6** exhibited the best activity as an antioxidant agent compared to ascorbic acid as a reference compound. Moreover, all compounds were evaluated as antimicrobial agents against a series of bacteria and fungi. Among all synthesized compounds, compound **6** achieved high efficiency against two types of fungi and four kinds of bacteria, as Clotrimazole and Ampicillin were used as the reference agents, respectively. All chemical structures of the novel synthesized candidates were unequivocally elucidated and confirmed utilizing spectroscopical and elemental investigations.

## 1. Introduction

Synthesis of heterocyclic candidates is a very effective approach in the drug discovery field. Five-membered ring individuals had a significant effect among heterocyclic compounds in both chemistry and biology tracks [1]. Remarkably, imidazole and thiazole nuclei exhibited a wide variety of different significant biological activities. Imidazole derivatives achieved potential results as effective drugs such as antioxidants [2], anti-inflammatory [3], antimicrobial [4], and so on. Another unique five-membered ring is thiazole. For a long time, thiazole derivatives were of great interest to scientists due to their significant and numerous pharmacological activities [5]. Thiazole derivatives have shown promise as therapeutic medicines such as anticancer [6], antimicrobial [7], antidiabetic agents [8], antiviral agents [9]. The fused imidazole was designed and drug discovery because it has a tremendous synthetic versatility that permits structural modifications throughout its periphery. Purine, one of the most important heterocyclic moieties discovered, includes the imidazole ring. Purines and pyrimidines have numerous pharmacological activities as they were an essential part of DNA and RNA as nitrogenous bases [10]. Purine derivatives achieved various biological activities as drugs [11]. On the other hand, the pyrimidine ring has unique pharmacological activities [12]. In this regard, chemists make many and various attempts to discover novel pyrimidine derivatives via different synthetic strategies and utilize various important catalysts [13,14,15]. Fused pyrimidines were designed and evaluated as antitumors [16], antioxidants [17], and anticancer [18]. In addition, imidazopyrimidines exhibited highly biological activities where they act as anti-inflammatory [19], antimicrobial [20], and cytotoxic agents [21]. Researchers designed effective drugs by reacting pyridine ring with nucleus pyrimidine ring as antioxidants [22], antimicrobial [23], and anticancer [24]. So, Hugh efforts have been made to construct this category of these heterocyclic candidates by inspired scientists [22,23,24]. Moreover, in light of the relevance of the aforementioned concerns and our ongoing efforts to synthesize novel condensed imidazole and thiazole systems, we present in this paper utilization imidazoldin-4-one, and thiazol-4-one as reactive synthones for the development of some novel fused thiols, imidazoles, purines, and pyrimidines, respectively.

The present study aimed to test the antioxidant properties and antimicrobial activity. Among the most dangerous species on the human body and living organisms, in general, are reactive oxygen species (ROS) naturally produced in the human body [25]. However, ROS overproduction endangers the human body in several ways, posing a range of hazards [26]. Vitamin C (ascorbic acid) has significant inhibitory properties that can minimize the hazards of ROS in the human body and other microorganisms such as Streptococcus pneumoniae and Saccharomyces cerevisiae [27]. Thus, we targeted synthesizing moieties expected to exhibit similar inhibitory activities of ascorbic acid as imidazoldin-4-one and thiazol-4-one. We also used molecular docking to investigate our compounds, which predicts the most common binding mode(s) of a ligand with a protein in a three-dimensional structure, out of our interest in ascorbic acid and moieties similar to ascorbic acid structure and functions. Thus, we choose appropriate proteins (PDB code: 2X08 and PDB code: 1F9G) where ascorbic acid is presented as an original ligand in those proteins [28,29]. Docking is a powerful tool for finding the best candidates by doing virtual screening on huge drug libraries and making structural assumptions about how the ligands inhibit the target [30,31].

## 2. The Rationale of the Work

**Figure 1:**
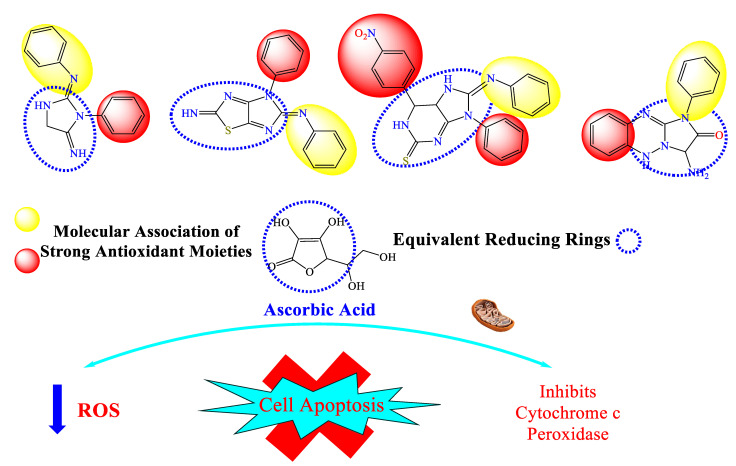
The rationale of the newly synthesized imidazole and thiazol derivatives using molecular association.

In our previous work, we designed and synthesized some new antioxidant compounds [22]. Continuing our efforts in this approach, we designed and synthesized a new series of promising antioxidant candidates. Our strategy was to synthesize candidates containing different reducing moieties (Thiazoles, pyrazoles, imidazotiazine, and purine-2-thiones); each one is encapsulated between two possible antioxidant moieties such as phenyl derivatives rings. These moieties exhibit reductive characteristics comparable to and even surpass those of ascorbic acid. Employing molecular docking to predict the potential antioxidant activities of these candidates is shown in Figure 1.

## 3. Result and Discussion

### 3.1. Chemistry

Previously, we have designed and synthesized a novel series of 1*H*-3-indolyl derivatives, which achieved high levels of activities as antioxidants [22]. Our vision in this work is to synthesize two novel series of promised five-membered rings: imidazoles and thiazoles. In this approach, we targeted candidates expected vital activities against bacterial infections and oxidants that the human body suffers.

**Scheme 1:**
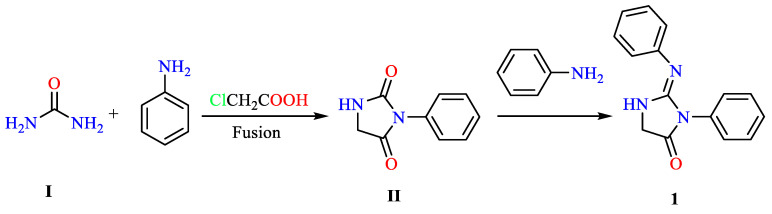
Synthesis and cyclization of imidazole **1**.

Firstly, we aimed to synthesize various novel imidazoles in a simple traditional way. A ternary mixture of urea, aniline, and chloroacetic acid undergo thermal cyclization without solvent to produce *N*-phenylimidazole condensed with another molecule of aniline to furnish (*E*)-3-phenyl-2-(phenylimino) imidazolidin-4-one 1 (Scheme 1). Compound **1** showed NH peaked at 3480 cm^−1^ while C=O was located at 1712 cm^−1^ due to the involvement of nitrogen (N) lone pair in aromatic resonance. The NH signal was detected at 11.4 ppm in addition to the aromatic multiplet and CH_2_ signal.

**Scheme 2:**
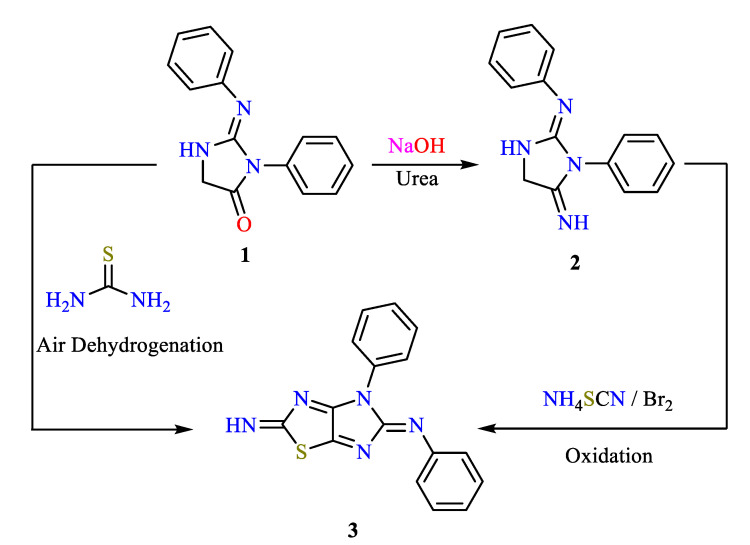
Synthesis of 4-diphenyl-2*H*-imidazo[4,5-*d*] thiazole-2,5(4*H*)-diimine by two different methods and reagents.

Subjecting of imidazole derivative **1** to react with urea / NaOH mixture provided iminoimidazole derivative **2** (Scheme 2). IR spectrum **2** contained NH and C=N peaks 3331 cm^−1^ and 1649 cm^−1^, respectively. The D_2_O exchangeable NH and NH_2_ signals were located at 11.20, 8.67 ppm, while CH_2_ was observed at 4.44 ppm. Compound **2**, NH_4_SCN, and Br_2_ in acetic acid led to oxidation, followed by the addition of exocyclic imino function to cyano group affording thiazole ring **3** (Scheme 2). Another direct way to generate the exact thiazole ring derivative **3** was depicted in (Scheme 2). As delectated in Scheme 2, imidazole derivative **1** was condensed with thiourea followed by air dehydrogenation providing thiazole cyclization **3.** The spectra of target **3** contain NH and C=N stretching frequency peaks at 3471 cm^−1^ and 1635 cm^−1^, respectively. The NH D_2_O exchangeable signal was observed at 8.86 ppm. 

**Scheme 3:**
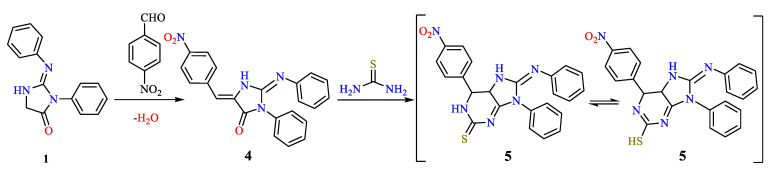
Synthesis of purine derivatives.

Imidazole **4** was generated from the condensation reaction of *p*-nitrobenzaldehyde with compound **1** (Scheme 3)**.** Compound **5** was synthesized by conjugate addition of thiourea to unsaturated system **4**. The NH and SH stretching absorption spectrum was observed around 3440 cm^−1^ while C=S was located at 1283 cm^−1^. The SH and NH D_2_O exchangeable signals were observed at 11.82, 10.16, and 10.07 ppm. The carbon signal of *sp^2^* thioxo carbonyl carbon was shown at 168.76 ppm.

**Scheme 4:**
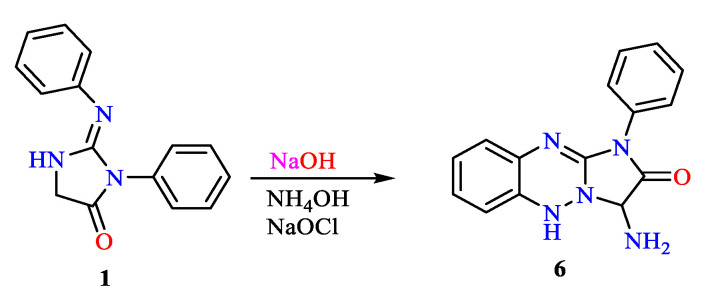
Synthesis of fused system imidazotriazine **6**.

**Scheme 5:**
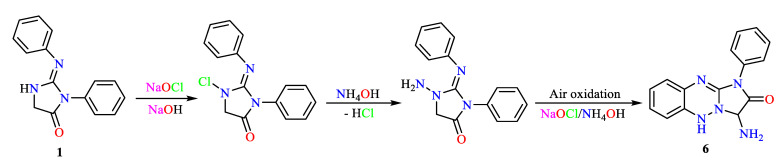
The proposed mechanism for the formation of compound **6**.

Compound **1** underwent *N*-amination followed by oxidative cyclization resulting in condensed triazine derivative **6** (Scheme 4). The formation of candidate **6** proposed underwent according to this mechanism illustrated in Scheme 5. The triazine derivative provided peaks for NH and C=O at 3267 cm^−1^ and 1701 cm^−1^ due to NH and CO groups, respectively. The NH downfield D_2_O exchangeable signals were observed at 10.46 and 8.65 ppm. The two different signals provided to methyl proton were located at 4.32 and 3.21 ppm.

**Scheme 6:**
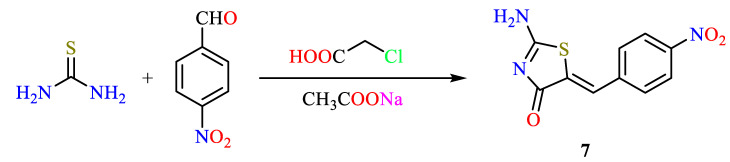
Synthesis of thiazole ring as the start for the second approach.

The second approach in our work is the generation of highly promising novel thiazole derivatives. Thiourea, *p*-nitrobenzaldehyde, chloroacetic acid, and sodium acetate were refluxed in an acidic medium to provide thiazole derivative (*Z*)-2-amino-5-(4-nitrobenzylidene) thiazol-4(5*H*)-one **7** (Scheme 6). Similar to 5-arylidene-4-thiazolidinones, in all cases, only Z-isomers were formed (confirmed by X-ray data) [32,33]. IR spectrum of **7** contained peaks at 3471 and 1677 cm^−1^ for NH and carbonyl groups. The NH_2_ signal was located at 12.8 ppm.

**Scheme 7:**
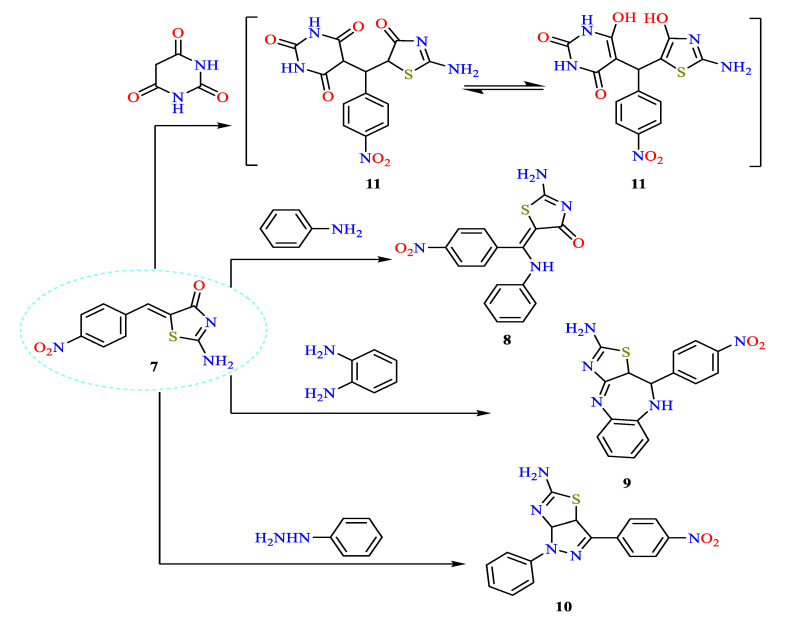
Synthesis of different fused systems composed of imidazole, pyridine, and pyrimidine rings with thiazole ring.

Aniline undergoes addition to *α, β*-unsaturated system of **7** followed by dehydrogenation to give (*E*)-2-amino-5-((4-nitrophenyl) (phenylamino)methylene)thiazol-4(5*H*)-one **8**. The *E*-configuration was assigned to structure **8** according to the Kandeel et al. study [34,35]. Kandeel et al. had identified the *E*- and *Z*-isomers of analogous 5-arylmethylene compounds. It was shown that the olefinic proton of the Z-configured isomers was more deshielded by the 4-oxo group of the thiazole moiety compared with the E-counterparts and appeared at the lower field (δ ≈ 8.00–8.20 ppm) relative to the E-isomer (δ ≈ 7.50–7.80 ppm). The heterocyclic **8** displayed frequencies for NH and CO at 3201 cm^−1^ and 1679 cm^−1^, respectively, and D_2_O exchangeable signal at 10.89 and 9.63 ppm. *o*-phenylenediamine undergoes 1,4 additions to compound **7** followed by cyclodehydration resulting in thiazolodiazepine derivative **9** (Scheme 7). The compound **9** leads to frequencies at 3400 cm^−1^ and 1604 cm^−1^ for NH and C=N, respectively. The NH D_2_O exchangeable signal was observed at 13.29 ppm.

Phenylhydrazine undergoes as a Micheal followed by cyclization and subsequent dehydrogenation of hydrogen to provide the fused product **10**. IR spectrum of **10** leads to NH peak at 3302 cm^−1^ and C=N at 1597 cm^−1^. The down shielded NH D_2_O exchangeable signal was observed at 10.89 ppm. Barbituric acid was added to the unsaturated system to provide the diol compound **11**. The structure of diol compound **11** was estimated by IR spectrum frequencies at 3471, 1674, and 1613 cm^−1^ for (NH, OH), (C=O), and (C=N), respectively. The D_2_O exchangeable downfield signals were observed at 11.36, 10.90, 10.33, 10.16, and 9.36 ppm for OH and NH, in addition to methyl proton shown at 2.90 ppm. The carbonyl carbon signal was detected at 180.41 ppm.

### 3.2. Biological Activity Studies

#### 3.2.1. Antioxidant Evaluation

**Figure 2:**
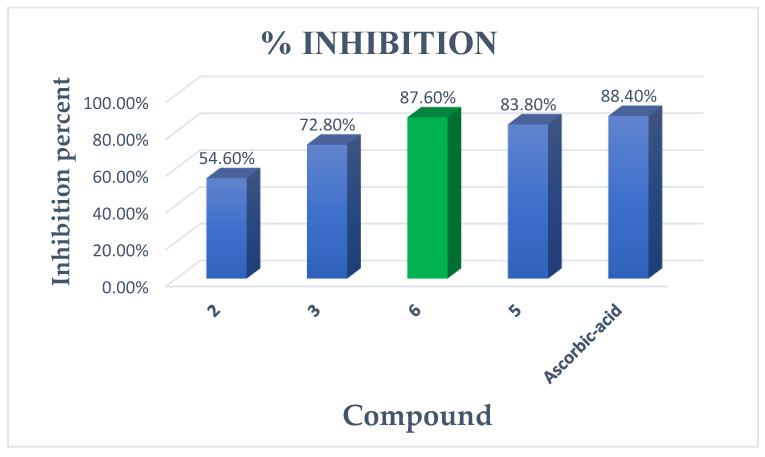
Antioxidant activity screening assay.

The antioxidant activities of the synthesized compounds were determined and listed in Table 1 and Figure 2. The results revealed that all compounds were found to be potent. Moreover, the results showed that compound **6** had the most potent levels of activity. Additionally, compounds **3** and **5** were found to have moderate activity. While compound **2** was found to have the lowest potent levels. In comparison between compound **6** and the other compounds, it was noticed that compound **6** indicated that the presence of the NH_2_ group was more effective than the other compounds. 

#### 3.2.2. Antimicrobial Activity

The activity of the synthesized candidates was tested and measured as antimicrobial agents. Firstly, they tested as antibacterial agents against *Bacillus subtilis* and *Staphylococcus aureus* as two Gram-positive bacteria, in addition to *Pseudomonas aeruginosa* and *Escherichia coli* as two Gram-negative bacteria. All tested candidates exhibited various potential activities as antibacterial agents, as shown in Table 2. Compound **6** exhibited an inhibitory and antibacterial effect on the four bacteria kinds like the qualified reference compound already known for its potent effect (Ampicillin).

Secondly, they tested as antifungal agents against two common fungi *Aspergillus flavus* and *Candida albicans*. Compound **6** achieved high efficiency against two types of fungi as Clotrimazole was used as the reference agent.

A modified Kirby-Bauer disc diffusion technique was used to evaluate the antimicrobial activity of the candidates tested. To summarise, 100 μL of test bacteria/fungus were cultivated in 10 mL of a new medium until a count of about 108 cells/mL for bacteria and 105 cells/mL for fungi was obtained [36].

### 3.3. Docking Studies 

On our way to predicting these synthetic compounds’ eligibility and theoretically validating their activity as an antioxidant, we modeled them with the appropriate protein under the molecular docking MOE program. So, we carried out a molecular docking study for new synthesized thiols and imidazoles in our work according to co-crystallized ascorbic acid as a reference ligand isolated from cytochrome *c* peroxidase enzyme (PDB code: 2X08) [28]. It set up four H-bonds with His181, Gly41, Val45, and Arg184 amino acids to bind to the γ-heme edge of cytochrome c peroxidase.

Our candidates exhibited very promising binding scores (**3** = −6.47, **5** = −5.49, **6** = −5.79 kcal/mol) compared to the binding score of ascorbic acid (−5.26 kcal/mol), as shown in Table 3. These results lead us to conclude that these compounds are stabilized inside the binding pocket of cytochrome *c* peroxidase. Moreover, ascorbic acid was stabilized inside the binding pocket of cytochrome *c* peroxidase via bound four H-bonds with Lys179, Leu177, Lys179, and Arg184 at 3.09, 2.87, 3.21, and 3.09 Å, respectively. While candidates **2**, **3**, **5**, and **6** were stabilized in the binding pocket by binding with crucial amino acids, as shown in Table 3. Firstly, compound **2** was bound to Gly41 via one H-bond and Asp37 one ionic bond at 2.97, 3.83 Å. Secondly, compound **3** was bound to Ser185, Arg184, and Thr180 through three H-bonds at 3.03, 3.46, and 4.23 Å, respectively. Thirdly, compound **5** was bound to Tyr42, Arg184, and His181 through three H-bonds at 3.28, 3.13, and 4.83 Å, respectively. Finally, the best one, compound **6,** was bound to three crucial amino acids Gly41, Asp37, and Arg184 via four H-bonds at 2.68, 2.79, 3.05, and 2.78 Å, respectively. The 2D and 3D receptor interactions of the prepared ligands with the largest pocket (2X08) protein were depicted in Table 4, and for further revision, these figures are included in Supplementary Materials details Figures S1–S10.

Our synthesized candidates did not exhibit significant antibacterial or antifungal activities against the series of bacteria and fungi mentioned above in Table 2. However, by studying the proposed mechanism of action for the newly designed and synthesized drug candidates through molecular docking against the *Streptococcus pneumoniae* bacteria, these candidates have significant results. Docking results proposed that these series of synthesized candidates have considerable potential activities to inhibit the action of the *Streptococcus pneumoniae* hyaluronate lyase enzyme. We carried out a molecular docking study for our synthesized thiols and imidazoles according to co-crystallized ascorbic acid as a reference ligand extracted from *Streptococcus pneumoniae* hyaluronate lyase enzyme (PDB code: 1F9G) [29]. Vitamin C exhibited significant inhibition enzymatic activity of *Streptococcus pneumoniae* hyaluronate lyase enzyme through binding to the enzyme active site and competing with the binding of the hyaluronan substrate via bound with five crucial amino acids (Arg243, Asn290, Trp292, Tyr408, and Asn580) [29]. In addition to its antioxidant and free radical scavenging activities, the inhibitory impact demonstrates that Vc is undoubtedly actively involved in inhibiting bacterial invasion. 

Table 5 shows that compounds (**2**, **3**, **5**, and **6**) got stabilized inside the binding pocket of streptococcus pneumoniae hyaluronate lyase with very promising binding scores of −5.41, −5.40, −7.13, and −5.76 kcal/mol, respectively, compared with that of ascorbic acid (−4.54 kcal/mol). This is good evidence that these compounds are stabilized inside the binding pocket of the streptococcus pneumoniae hyaluronate lyase enzyme, and they have significant interaction with it. Moreover, ascorbic acid was stabilized inside the binding pocket of streptococcus pneumoniae hyaluronate lyase enzyme via bound three H-bonds with His399, Asn-580, and Asn-290 at 3.40, 3.02, and 3.01 Å, respectively. While candidates **2**, **3**, **5**, and **6** were stabilized in the binding pocket by binding with crucial amino acids, as shown in Table 5. Compound **2** was bound via two hydrogen bonds to Trp292 and Asn290 and with His399 through pi-cation at 3.79, 2.98, 4.50 Å, respectively. While compound **3** was bound to five crucial amino acids; Asn-290 through one H-bond and Asp293 through ionic bond and His399 through pi-cation and Trp292 and Tyr408 via pi-pi force at 2.97, 3.49, 4.03, 3.98, and 3.94 Å, respectively. Furthermore, compound **5** was bound to six crucial amino acids. It bound through four H-bonds with Arg466, His399, Trp291, and Asn290 at 4.48, 3.14, 3.19, and 3.59 Å, respectively. At the same time, it is bound with Arg462 via pi-cation at 3.64 Å and with Trp292 via at 3.99 Å. Finally, the best one, compound **6**, was bound to three crucial amino acids, Tyr408, Trp292, and His399 via two H-bonds and one pi-cation at 3.09, 3.78, and 4.28 Å, respectively. The 2D and 3D receptor interactions of the prepared ligands with the largest pocket (1F9G) protein were depicted in Table 6, and for further revision, these figures are included in Supplementary Materials details Figures S11–S20.

## 4. Conclusions

Out of our vision, we have designed and synthesized a novel combination of compounds starting from two promising bioactive moieties imidazoldin-4-one and thiazol-4-one. Our candidates achieved significant antioxidant activities compared to ascorbic acid as a reference standard compound, especially compound **6**. Compound **6** exhibits a high level of activity as an antioxidant via ABTS assay, where the percentage of inhibition was 87.6% while ascorbic acid was 88.4%. Otherwise, we tested our candidates as antimicrobial against crucial series of Gram-positive bacteria, Gram-negative bacteria, and fungi. Unfortunately, their results were disappointing. Nevertheless, examining these compounds as Streptococcus pneumoniae hyaluronate lyase enzyme inhibitors via molecular docking gives promising results. Docking solutions revealed that candidates **2**, **3**, **5**, and **6** are potent inhibitors for this enzyme revealed the stabilization of the ligand inside the enzyme pocket. Therefore, these compounds could be promising Streptococcus pneumoniae hyaluronate lyase enzyme inhibitors compared to ascorbic acid. Thus, the next goal in this approach will be to demonstrate the activity of these compounds in vitro and in vivo as Streptococcus pneumoniae hyaluronate lyase enzyme inhibitors.

## 5. Experimental

### 5.1. Antioxidant Screening Assay (ABTS Method) 

Essential chemicals such as *L*-Ascorbic acid and 2, 20-azinobis-(3-ethylbenzthiazoline-6-sulphonic acid) (ABTS) were purchased from Sigma 2 mL ABTS solution (60 mM) was added to 3 M magnesium oxide (MnO_2_) solution (25 mg/mL) in phosphate buffer for each of the studied compounds (pH 7, 0.1 M). Firstly, the absorbance (A control) of the resultant green-blue solution (ABTS radical solution) was set at ca. 0.5 at 734 nm after shaking, centrifuging, and filtering. After that, add 50 mL of a 2 mM test chemical solution in spectroscopic grade methanol/phosphate buffer (1:1). The absorbance (A test) was determined, and the percentage inhibition of color intensity was calculated. The following equation, Equation (1), was used to determine the inhibition of each compound.

% Inhibition = [A (control) − A (test)/ A (Control)] × 100(1)

The typical antioxidant was ascorbic acid (vitamin C) (positive control). Instead of a sample, a blank sample was run without ABTS and with methanol/phosphate buffer (1:1) instead. Instead of the tested chemical, a negative control sample was performed using a 1:1 mixture of methanol and phosphate buffer [37].

### 5.2. Antimicrobial Screening Assay:

The activity of the synthesized candidates was tested and measured as antimicrobial agents. Firstly, they tested as antibacterials against *Bacillus subtilis*, *Staphylococcus aureus*, and as two Gram-positive bacteria, in addition to *Pseudomonas aeruginosa*, *Escherichia coli* as two Gram-negative bacteria. Secondly, they tested as antifungal agents against two common fungi *Aspergillus flavus* and *Candida albicans*. Each drug was dissolved in DMSO, and a solution with a concentration of 1 mg/mL was produced individually. Whitman filter paper discs of standard size (5 cm) were cut and sterilized in an autoclave. The paper discs were placed aseptically in Petri dishes containing nutrient agar media (agar 20 g + beef extract 3 g + peptone 5 g) seeded with Staphylococcus aureus, Bacillus subtilis, E. coli, Pseudomonas aeruginosa, Candida albicans, and Aspergillus flavus, soaked in the desired concentration of the complex solution. The inhibitory zones were measured after 24 h of incubation at 36 degrees Celsius in Petri dishes. Three times each therapy was carried out. Antibacterial and antifungal activities of ampicillin as common standard antibiotic and antifungal Clotrimazole were also measured using the same technique and solvents as previously. The formula calculated the % activity index for the complex as showed in Equation (2):

% Activity Index = Zone of inhibition by test compound (diameter) / Zone of inhibition by standard (diameter)(2)

### 5.3. Chemistry

All chemicals were purchased from Sigma-Aldrich (Taufkirchen, Germany), and all solvents were purchased from El-Nasr Pharmaceutical Chemicals Company (analytical reagent grade, Egypt). All chemicals were used as supplied without further purification. The melting points were measured by a digital Electrothermal IA 9100 Series apparatus Cole-Parmer, Beacon Road, Stone, Staffordshire, ST15 OSA, UK) and were uncorrected. C, H, and N analyses were carried out on a PerkinElmer CHN 2400. IR spectra were recorded on FT-IR 460 PLUS (KBr disks) in the range from 4000 to 400 cm^−1^. ^1^H and ^13^C-NMR spectra were recorded on a Bruker 400 MHz NMR Spectrometer using tetramethylsilane (TMS) as the internal standard, chemical shifts are expressed in δ (ppm), and DMSO-*d*_6_ was used as the solvent. At the Regional Centre for Mycology & Biotechnology (RCMB) Al-Azhar University, Naser City, Cairo.

**(*E*)-3-Phenyl-2-(phenylimino) imidazolidin-4-one (1)**

A mixture of phenylamine (10 mmol, 0.94 gm), urea (10 mmol, 0.69 gm), and chloroacetic acid (10 mmol, 0.95 gm) was consolidated for 30 min then triturated with hot dilute methanol. The reaction mixture was cooled, and therefore the separated solid product was filtered off, dried, crystallized from ethanol, and gave buff crystals of compound **1**. Yield: 77%; m.p. 140 °C. IR (KBr, ν, cm^−1^): broadband 3450–3500 cm^−1^ (NH str), 1712 cm^−1^ (C=O), 1595 cm^−1^ (C=N str). ^1^H NMR (DMSO-*d*_6_, 400 MHz): δ = 4.62 ppm (s, 2H, CH_2_), 7.40–7.69 ppm (m, 10H, ArH’s), 11.4 ppm (s, H, NH). Analytical for C_15_H1_3_N_3_O (251.29); calcd:C, 71.65; H, 5.22; N, 16.68; found:C, 71.70; H, 5.21; N, 16.72.

**(E)-N2,3-Diphenylimidazolidine-2,4-diimine (2)**

A mixture of imidazolidin-4-one **1** (10 mmol, 2.5 gm), urea (10 mmol, 0.69 gm) and NaOH (10 mmol, 0.4 gm) was refluxed for 4 h. The reaction mixture was cooled, and the formed solids was collected filtration, dried, crystallized from ethanol to produce yellow crystals of compound **2.** Yield: 55%; m.p. 200 °C. IR (KBr, ν, cm^−1^): broad band 3370–3500 cm^−1^ (2NH str), 1597 cm^−1^ (C=N str). ^1^H NMR (DMSO-*d*_6_, 400 MHz): δ = 4.62 ppm (s, 2H, CH_2_), 6.96–7.62 ppm (m, 10H, ArH’s), 8.67 ppm (s, 2H, NH_2_ exchangeable by D_2_O),11.2 ppm (s, H, NH exchangeable by D_2_O). ^13^C NMR (DMSO-*d*_6_, 100 MHz): δ = 39.50, 50.90, 118.13, 118.16, 121.81, 123.34, 128.82, 128.92, 138.18, 139.71, 152.53, 154.86, 170.50 ppm. Analytical for C_15_H1_4_N_4_ (250.31); calcd: C, 71.89; H, 5.60; N, 22.34; found:C, 71.98; H, 5.64; N, 22.38.

**(Z)-N5,4-Diphenyl-2H-imidazo [4,5-d] thiazole-2,5(4H)-diimine (3)**

**This compound was prepared via two methods.**

**Firstly:** A mixture of imidazolidine-2,4-diimine **2** (10 mmol, 2.5 gm), NH_4_SCN (10 mmol, 0.76 gm) and bromine (10 mmol, 1.6 gm) in acetic acid (20 mL) was left over night. the precipitate formed up on dilution was filtered off, dried, crystallized from ethanol to give brown crystal of compound **3**. Yield: 57%; m.p. 215 °C. IR (KBr, ν, cm^−1^): broad band 3460–3500 cm^−1^ (2NH str), 1635 cm^−1^ (C=N str). ^1^H NMR (DMSO-*d*_6_, 400 MHz): δ = 7.15–7.71 ppm (m, 10H, ArH’s), 8.6 ppm (s, H, NH exchangeable by D_2_O). Analytical for C_17_H_12_N_4_S (304.37); calcd: C, 67.10; H, 3.91; N, 18.35; S, 10.50; found:C, 67.08; H, 3.97; N, 18.41; S, 10.53.

**Secondly:** Also, the targeted molecule **3** was synthesized by refluxing a mixture of imidazolidin-4-one **1** (10 mmol, 2.5 gm), thiourea (10 mmol, 0.76 gm), and NaOH (10 mmol, 0.4 gm) in ethanol (10 mmol, 0.5 gm) for 6 h. The reaction mixture was cooled to produce the precipitate that was filtered off, dried, crystallized from ethanol, and gave yellow crystals of **3**. Yield: 69%; m.p. 215 °C. IR (KBr, ν, cm^−1^): broadband 3335–3290 cm^−1^ (NH str), 3032 cm^−1^ (CH str Ar), 1651 cm^−1^ (C=N str). ^1^H NMR (DMSO-*d*_6_, 400 MHz): δ = 7.47–6.95 ppm (m, 10H, ArH’s), 8.65 ppm (s, H, NH exchangeable by D_2_O). Analytical for C_16_H_11_N_5_S (305.36); calcd: C, 62.90; H, 3.58; N, 22.89; S, 10.45; found: C, 62.93; H, 3.63; N, 22.94; S, 10.50.

**(Z)-5-((Z)-4-Nitrobenzylidene)-3-phenyl-2-(phenylimino) imidazolidin-4-one (4)**

A mixture of imidazolidin-4-one **1** (10 mmol, 2.5 gm), *p*-nitrobenzaldehyde (10 mmol, 1.5 gm), and sodium metal (10 mmol, 0.23 gm) in ethanol (20 mL) was refluxed for 6 h. The separated solid product was collected by filtration, dried, crystallized from ethanol 95% to give green crystals of compound **4**. Yield: 65%; m.p. 280 °C. IR (KBr, ν, cm^−1^): broadband 3260–3420 cm^−1^ (NH str), 1769 cm^−1^ (C=O). ^1^H NMR (DMSO-*d*_6_, 400 MHz): δ = 4.95 ppm (s, H, CH methyl), 6.96–8.07 ppm (m, 14H, ArH’s), 10.79 ppm (s, H, NH). Analytical for C_22_H_16_N_4_O_3_ (384.40); calcd:C, 68.70; H, 4.16; N, 14.53; found: C, 68.74; H, 4.20; N, 14.58.

**(Z)-6-(4-Nitrophenyl)-9-phenyl-8-(phenylimino)-5,7,8,9-tetrahydro-6H-purine-2-thiol (5)**

A mixture of imidazolidin-4-one **1** (10 mmol, 2.5 gm), thiourea (10 mmol, 0.76 gm) and *p*-nitrobenzaldehyde (10 mmol, 1.5 gm) was refluxed for 4 h. The separated solid product was collected by filtration, dried, crystallized from ethanol to provide black crystals of compound **5**. Yield: 64%; m.p. 220 °C. IR (KBr, ν, cm^−1^): broadband 3300–3500 cm^−1^ (NH and SH str), 1597 cm^−1^ (C=N str), 1283 cm^−1^ (C=S str). ^1^H NMR (DMSO-*d*_6_, 400 MHz): δ = 4.22 ppm (s, H, CH methyl proton), 7.04–8.36 ppm (m, 14H, ArH’s), 10.16 ppm (s, H, NH_2_ exchangeable by D_2_O), 11.82 ppm (s, H, SH exchangeable by D_2_O). ^13^C NMR (DMSO-*d*_6_, 100 MHz): δ = 39.43, 40.27, 114.10, 119.08, 122.49, 123.02, 123.95, 123.98, 124.45, 125.71, 129.35, 130.37, 130.88, 133.06, 134.60, 139.90, 146.89, 150.53, 166.71, 167.07 ppm, and 168.76 ppm for (C=S). Analytical for C_23_H_18_N_6_O_2_S (442.50); calcd:C, 62.35; H, 4.00; N, 18.90; S, 7.19; found:C, 62.43; H, 4.10; N, 18.99; S, 7.25.

**3-Amino-1-phenyl-1,5-dihydrobenzo[e]imidazo[1,2-b]** [1,2,4] **triazin-2(3H)-one (6)**

A mixture of imidazolidin-4-one **1** (10 mmol, 2.5 gm), NaOCl (10 mmol, 0.75 gm), NH_4_OH (10 mmol, 0.35 gm), and NaOH (10 mmol, 0.4 gm) in ethanol (20 mL) at room temperature and left for an hour. The produced solids were collected by filtration, dried, crystallized from ethanol to furnish yellow crystals of compound **6**. Yield: 58%; m.p. 230 °C. IR (KBr, ν, cm^−1^): broad band 3320–3260 cm^−1^ (NH and NH_2_ str), 3035 cm^−1^ (CH str Ar), 1701 cm^−1^ (C=O). ^1^H NMR (DMSO-*d*_6_, 400 MHz): δ = 7.60–6.53 ppm (m, 9H, ArH’s), 8.65 ppm (s, 2H, NH_2_ exchangeable by D_2_O), 10.46 ppm (s, H, NH exchangeable by D_2_O). Analytical for C_15_H_13_N_5_O (279.30); calcd: C, 64.48; H, 4.63; N, 25.10; found: C, 64.51; H, 4.69; N, 25.07.

**(Z)-2-Amino-5-(4-nitrobenzylidene) thiazol-4(5H)-one (7)**

A mixture of *p*-nitrobenzaldehyde (10 mmol, 1.5 gm), thiourea (10 mmol, 0.76 gm), and chloroacetic acid (10 mmol, 0.95 gm) in acetic acid (20 mL) was refluxed for 6 h. The reaction mixture was cooled, and the formed solid product was collected by filtration, dried, crystallized from ethanol to give yellow crystals of compound **7**. Yield: 60%; m.p. 203 °C. IR (KBr, ν, cm^−1^): broad band 3490–3395 cm^−1^ (NH_2_ str), 3048 cm^−1^ (CH str Ar), 1677 cm^−1^ (C=O). ^1^H NMR (DMSO-*d*_6_, 400 MHz): δ = 7.81 ppm (d, H, CH_2_), 8.29–8.31 ppm (m, 3H, ArH), 12.79 ppm (s, 2H, NH_2_). Analytical for C_10_H_7_N_3_O_3_S (249.24); calcd: C, 48.14; H, 2.78; N, 16.81; S, 12.82; found:C, 48.19; H, 2.83; N, 16.86; S, 12.86.

**(E)-2-Amino-5-((4-nitrophenyl) (phenylamino)methylene) thiazol-4(5H)-one (8)**

A mixture of benzylidenethiazol-4(5*H*)-one **7** (10 mmol, 2.5 gm) and aniline (10 mmol, 0.93 gm) in dimethylformamide (15mL) was refluxed for 6 h. The reaction mixture was cooled and the solid formed upon concentration to furnish black precipitate of compound **8**. Yield: 65%; m.p. 262 °C. IR (KBr, ν, cm^−1^): broad band 3205–3150 cm^−1^ (NH_2_ and NH str), 1679 cm^−1^ (C=O). ^1^H NMR (DMSO-*d*_6_, 400 MHz): δ = 8.35–6.60 ppm (m, 9H, ArH’s), 9.36 ppm (s, 2H, NH_2_ exchangeable by D_2_O), 9.63 ppm (s, H, NH exchangeable by D_2_O). ^13^C NMR (DMSO-*d*_6_, 100 MHz): δ = 39.50, 40.25, 112.68, 124.23, 124.23, 126.46, 128.97, 130.28, 130.88, 133.85, 139.37, 140.67, 146.94, 167.16, 167.44, 175.18 ppm, and (C=O) at 179.84 ppm. Analytical for C_16_H_12_N_4_O_3_S (340.36); calcd:C, 56.44; H, 3.57; N, 16.40; S, 9.47; found:C, 56.46; H, 3.55; N, 16.46; S, 9.42.

**10-(4-Nitrophenyl)-10,10a-dihydro-9H-benzo[b]thiazolo[4,5-e]** [1,4] **diazepin-2-amine (9)**

A mixture of benzylidenethiazol-4(5*H*)-one **7** (10 mmol, 2.5 gm) and *o*-phenylenediamine (10 mmol, 1.08 gm) in dimethylformamide (15mL) was refluxed for 6 h. The reaction mixture was cooled, and the separated solid product was filtered off, dried, crystallized from ethanol, and yield red crystals of compound **9**. Yield: 50%; m.p. 300 °C. IR (KBr, ν, cm^−1^): broadband 3450–3350 cm^−1^ (NH_2_ str), 1604 cm^−1^ (C=N str). ^1^H NMR (DMSO-*d*_6_, 400 MHz): δ = 8.45–8.40 ppm (m, 8H, ArH’s), 13.29 ppm (s, 2H, NH_2_ exchangeable by D_2_O). Analytical for C_16_H_11_N_5_O_2_S (337.36); calcd: C, 56.90; H, 3.23; N, 20.71; S, 9.53; found:C, 56.97; H, 3.29; N, 20.76; S, 9.50.

**3-(4-Nitrophenyl)-1-phenyl-3a,6a-dihydro-1H-pyrazolo[3,4-d] thiazol-5-amine (10)**

A mixture of benzylidenethiazol-4(5*H*)-one **7** (10 mmol, 2.5 gm) and phenylhydrazine (10 mmol, 1.08 gm) in dimethylformamide (15 mL) was refluxed for 6 h. The reaction mixture was cooled, and the isolated precipitate was filtered off, dried, crystallized from ethanol, and gave brown crystals of compound **10**. Yield: 52%; m.p. 150 °C. IR (KBr, ν, cm^−1^): broadband 3350–3250 cm^−1^ (NH_2_ str), 3055 cm^−1^ (CH str Ar), 1679 cm^−1^ (C=O), 1597 cm^−1^ (C=N str). ^1^H NMR (DMSO-*d*_6_, 400 MHz): δ = 8.36–6.82 ppm (m, 9H, ArH’s), 9.63 ppm (s, 2H, NH_2_ exchangeable by D_2_O), 10.89 ppm (s, H, NH exchangeable by D_2_O). Analytical for C_16_H_15_N_5_O_2_S (341.39); calcd:C, 56.25; H, 4.38; N, 20.48; S, 9.35; found:C, 56.29; H, 4.43; N, 20.51; S, 9.39.

**5-((2-Amino-4-hydroxythiazol-5-yl) (4-nitrophenyl)methyl)-6-hydroxypyrimidine-2,4(1H,3H)-dione (11)**

A mixture of benzylidenethiazol-4(5*H*)-one **7** (10 mmol, 2.5 gm) and barbituric acid (10 mmol, 1.3 gm) in dimethylformamide (15mL) was refluxed for 6 hrs. The reaction mixture was cooled, and the isolated solid product was filtered off, dried, crystallized from ethanol, and furnished black crystals of compound **11**. Yield: 52%; m.p. above 300 °C. IR (KBr, ν, cm^−1^): broad band 3560–3450 cm^−1^ (NH_2_, NH, and OH str), 3222 cm^−1^ (CH str Ar), 1674 cm^−1^ (C=O), 1613 cm^−1^ (C=N str). ^1^H NMR (DMSO-*d*_6_, 400 MHz): δ = 2.90 ppm (s, H, CH methyl), 8.35–7.69 ppm (m, 4H, ArH’s), 9.36 ppm (s, 2H, NH_2_ exchangeable by D_2_O),10.16 ppm (s, H, NH exchangeable by D_2_O),10.33 ppm (s, H, NH exchangeable by D_2_O), 10.90 ppm (s, H, 2 OH exchangeable by D_2_O), 11.36 ppm (s, H, OH exchangeable by D_2_O). ^13^C NMR (DMSO-*d*_6_, 100 MHz): δ = 39.14, 39.35, 39.56, 34.88, 39.77, 40.18, 40.39, 124.70, 127.06, 130.74, 134.28, 141.07, 147.46, 175.83 ppm, and 180.41 ppm (C=O). Analytical for C_14_H_11_N_5_O_6_S (377.33); calcd: C, 44.51; H, 2.88; N, 18.52; S, 8.53; found: C, 44.56; H, 2.94; N, 18.56; S, 8.50.

## Figures and Tables

**Table 1:** Antioxidant assay for the tested new compounds.

Compounds	Absorbance of Samples	% Inhibition
**2**	0.227	54.6%
**3**	0.136	72.8%
**6**	0.062	87.6%
**5**	0.081	83.8%
**Ascorbic-acid**	0.058	88.4%
**Control of ABTS**	0.500	0

**Table 2:** In vitro antibacterial and antifungal screening of the newly synthesized compounds.

Compound	*E. Coli*		*Pseudomonas Aeuroginosa*		*S. Aureus*		*Bacillus Subtilis*		*C. Albicans*		*A. Flavus*	
	Diameter of Inhibition Zone (mm)	% Activity Index	Diameter of Inhibition Zone (mm)	% Activity Index	Diameter of Inhibition Zone (mm)	% Activity Index	Diameter of Inhibition Zone (mm)	% Activity Index	Diameter of Inhibition Zone (mm)	% Activity Index	Diameter of Inhibition Zone (mm)	% Activity Index
**3**	3	12	NA	----	2	18	NA	----	2	8	3	15
**5**	10	40	3	25	5	45.5	4	17.4	7	28	6	30
**2**	NA	----	NA	----	NA	----	NA	----	NA	----	2	10
**6**	20	80	11	91.7	10	90.9	9	39.1	12	48	15	75
**Ampicillin**	25	100	12	100	11	100	20	100	NA	----	NA	----
**Colitrimaz-ole**	NA	----	NA	----	NA	----	NA	----	25	100	20	100

NA: This compound has No Action or Negligible Action on this specific type of microorganism.

**Table 3:** The binding scores, RMSD values, receptor interactions, distances, and energies of our promising candidates (**2**, **3**, **5**, and **6**) were recorded compared to the docked ascorbic acid as a reference standard inside the binding pocket of cytochrome c peroxidase.

Ligand	Docking Score	RMSD	Recepitor Interaction	Distance Å	E (Kcal/mol)
**2**	−4.49	0.90	Gly-41/H-donor Asp-37/Ionic	2.97 3.83	−3.0 −0.9
**3**	−6.47	1.52	Ser-185/H-donor Arg-184/H-acceptor Thr-180/pi-H	3.03 3.46 4.23	−0.6 −1.1 −1.1
**5**	−5.49	1.65	Tyr-42/H-acceptor Arg-184/H-acceptor His-181/H-pi	3.28 3.13 4.83	−1.2 −2.7 −0.6
**6**	−5.79	1.37	Gly-41/H-donor Asp-37/H-donor Gly-41/H-donor Arg-184/H-acceptor	2.68 2.79 3.05 2.78	−3.9 −2.9 −1.9 −6.7
**Ascorbic acid** **(Docked)**	−5.26	0.80	Lys-179/H-donor Leu-177/H-donor Lys-179/H-donor Arg-184/H-acceptor	3.09 2.87 3.21 3.09	−0.7 −1.1 −1.2 −4.4

**Table 4:** 2D and 3D receptor interactions and receptor positioning of the most significant potential candidates (**2**, **3**, **5**, and **6**) compared to the docked ascorbic acid as a reference standard inside the binding pocket of cytochrome c peroxidase.

Molecule	2D Receptor Interaction	3D Receptor Interaction	3D Receptor Positioning
**2**	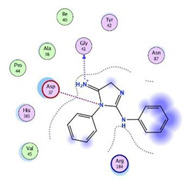	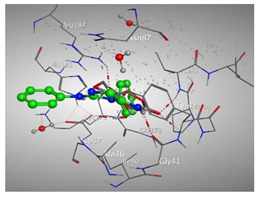	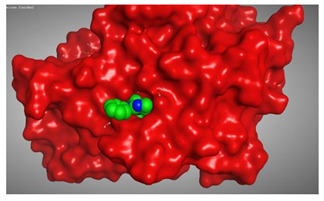
**3**	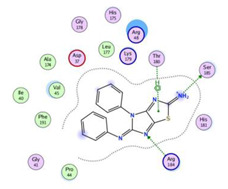	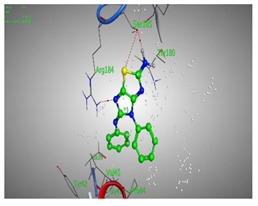	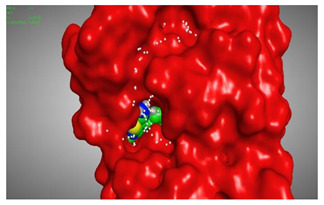
**5**	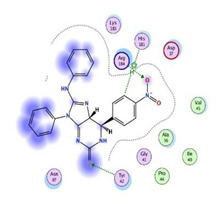	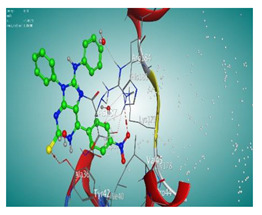	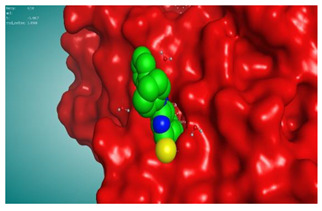
**6**	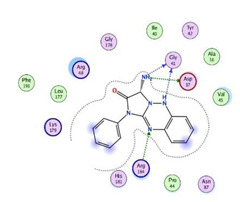	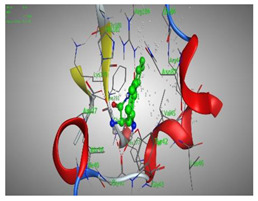	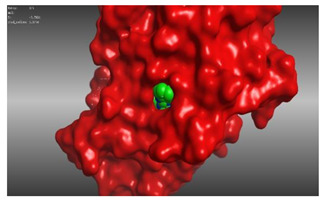
**Ascorbic Acid** **(Docked)**	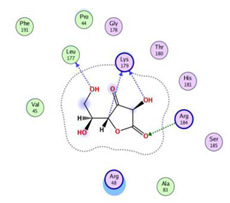	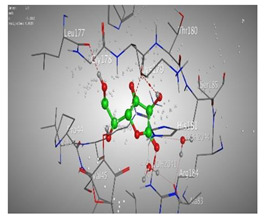	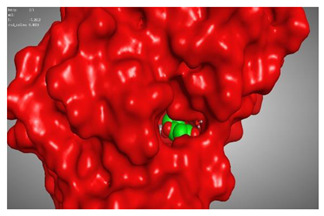

**Table 5:** The binding scores, RMSD values, receptor interactions, distances, and energies of our promising candidates (**2**, **3**, **5**, and **6**) were recorded compared to the docked ascorbic acid as a reference standard inside the binding pocket of Streptococcus pneumoniae hyaluronate lyase enzyme.

Ligand	Docking Score	RMSD	Recepitor Interaction	Distance Å	E (Kcal/mol)
**2**	−5.41	1.075	Trp-292/H-pi Asn-290/H-donor His-399/pi-cation	3.79 2.98 4.50	−0.8 −2.0 −1.0
**3**	−5.40	0.65	Asn-290/H-donor Asp-293/Ionic His-399/pi-cation Trp-292/pi-pi Tyr-408/pi-pi	2.97 3.49 4.03 3.98 3.94	−3.0 −1.9 −1.0 −0.0 −0.0
**5**	−7.13	1.70	Arg-466/H-acceptor His-399/H-acceptor Trp-291/H-acceptor Asn-290/pi-H Arg-462/pi-cation Trp-292/pi-pi	4.48 3.14 3.19 3.59 3.64 3.99	−1.2 −2.4 −1.1 −0.6 −0.6 −0.0
**6**	−5.76	0.89	Tyr-408/H-acceptor Trp-292/H-pi His-399/pi-cation	3.09 3.78 4.28	−1.2 −0.8 −1.0
**Ascorbic acid** **(Docked)**	−4.54	1.21	His-399/H-acceptor Asn-580/H-acceptor Asn-290/H-donor	3.40 3.02 3.01	−0.9 −1.4 −2.2

**Table 6:** 2D and 3D receptor interactions and receptor positioning of the most significant potential candidates (**2**, **3**, **5**, and **6**) compared to the docked ascorbic acid as a reference standard inside the binding pocket of Streptococcus pneumoniae hyaluronate lyase.

Molecule	2D Receptor Interaction	3D Receptor Interaction	3D Receptor Positioning
**2**	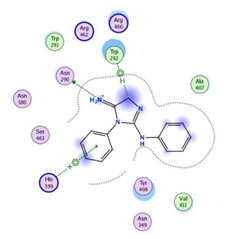	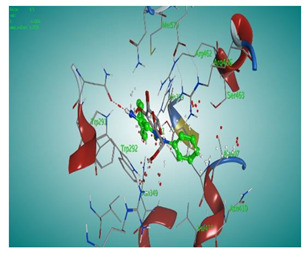	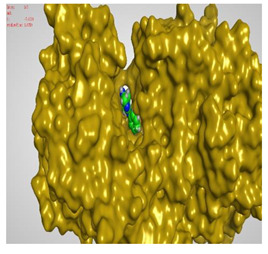
**3**	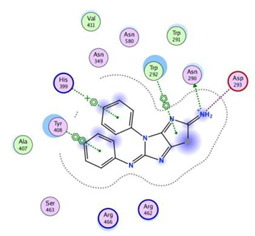	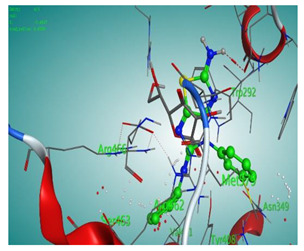	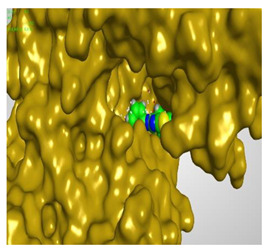
**5**	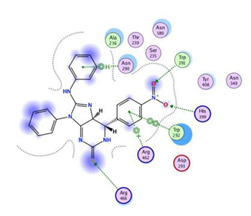	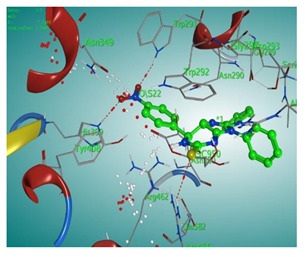	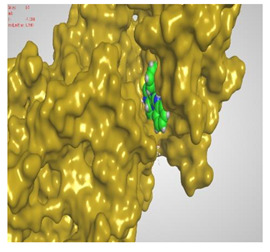
**6**	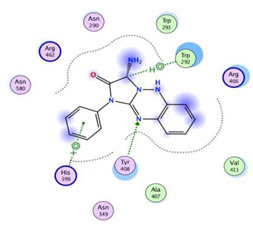	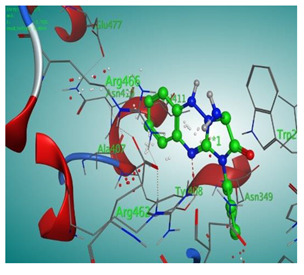	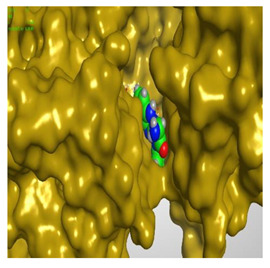
**Ascorbic** **Acid** **(Docked)**	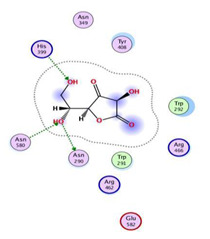	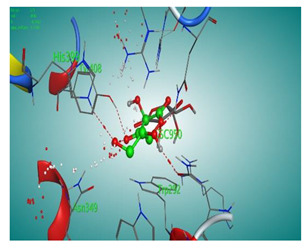	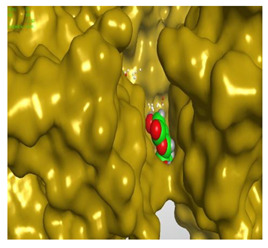

## Data Availability

Not applicable.

## Supplementary Materials

The following are available online. Figure S1: 2D interaction images of ascorbic acid with the active sites of 2X08, Figure S2: 3D interaction images of ascorbic acid with the active sites of 2X08, Figure S3: 2D interaction images of compound **2** with the active sites of 2X08, Figure S4: 3D interaction images of compound **2** with the active sites of 2X08, Figure S5: 2D interaction images of compound **3** with the active sites of 2X08, Figure S6: 3D interaction images of compound **3** with the active sites of 2X08, Figure S7: 2D interaction images of compound **5** with the active sites of 2X08, Figure S8: 3D interaction images of compound **5** with the active sites of 2X08, Figure S9: 2D interaction images of compound **6** with the active sites of 2X08, Figure S10: 3D interaction images of compound **6** with the active sites of 2X08, Figure S11: 2D interaction images of ascorbic acid with the active sites of 1F9G, Figure S12: 3D interaction images of ascorbic acid with the active sites of 1F9G, Figure S13: 2D interaction images of compound **2** with the active sites of 1F9G, Figure S14: 3D interaction images of compound **2** with the active sites of 1F9G, Figure S15: 2D interaction images of compound **3** with the active sites of 1F9G, Figure S16: 3D interaction images of compound **3** with the active sites of 1F9G, Figure S17: 2D interaction images of compound **5** with the active sites of 1F9G, Figure S18: 3D interaction images of compound **5** with the active sites of 1F9G, Figure S19: 2D interaction images of compound **6** with the active sites of 1F9G, Figure S20: 3D interaction images of compound **6** with the active sites of 1F9G.

## Sample Availability

Samples of the compounds, are available from the authors.
