# Supplementary material for: Design, Synthesis, Molecular Docking, and Evaluation Antioxidant and Antimicrobial Activities for Novel 3-phenylimidazolidin-4-one and 2-aminothiazol-4-one Derivatives
Source: Molecules. 2022 Jan 24;27(3):767. doi: 10.3390/molecules27030767 (PMC8840376; doi:10.3390/molecules27030767)
Supplement: Supplementary file 1 [file molecules-27-00767-s001.zip › molecules-1541471-supplementary.pdf]

Design, Synthesis, Molecular Docking, and Evaluation Antioxidant and Antimicrobial Activities for Novel 3-Phenylimidazolidin-4-One and 2-Aminothiazol-4-One Derivatives

Wesam S. Shehab [1,*,†], Maged A. Aziz [1,†], Nourhan Kh. R. Elhoseni [1], Mohamed G. Assy [1], Magda H. Abdellattif [2], and Eman O. Hamed [1]

Docking study

A molecular docking study of the sixteen newly designed and synthesized 3-phenylimidazolidin-4-one and 2-aminothiazol-4-one compounds was performed using MOE 2019.0102 program. The newly synthesized compounds were drawn using ChemDraw, imported into the MOE program window, converted for their 3D forms, adjusted for the partial charges, and energy minimized as described earlier. The database was built containing the newly synthesized candidates (**1–11**) together with the co-crystallized ascorbic acid as a reference standard. A general docking process was performed using the site of the co-crystallized ascorbic acid inside cytochrome *c* peroxidase and streptococcus pneumoniae hyaluronate lyase enzyme as the docking site. Also, all the other docking parameters were adjusted as previously discussed in detail. Moreover, it is worth mentioning that a program validation process was performed at first before applying the docking process by redocking the co-crystallized ascorbic acid at its binding pocket of the cytochrome *c* peroxidase enzyme and streptococcus pneumoniae hyaluronate lyase enzyme. A valid performance was confirmed by obtaining a low RMSD value (<1).

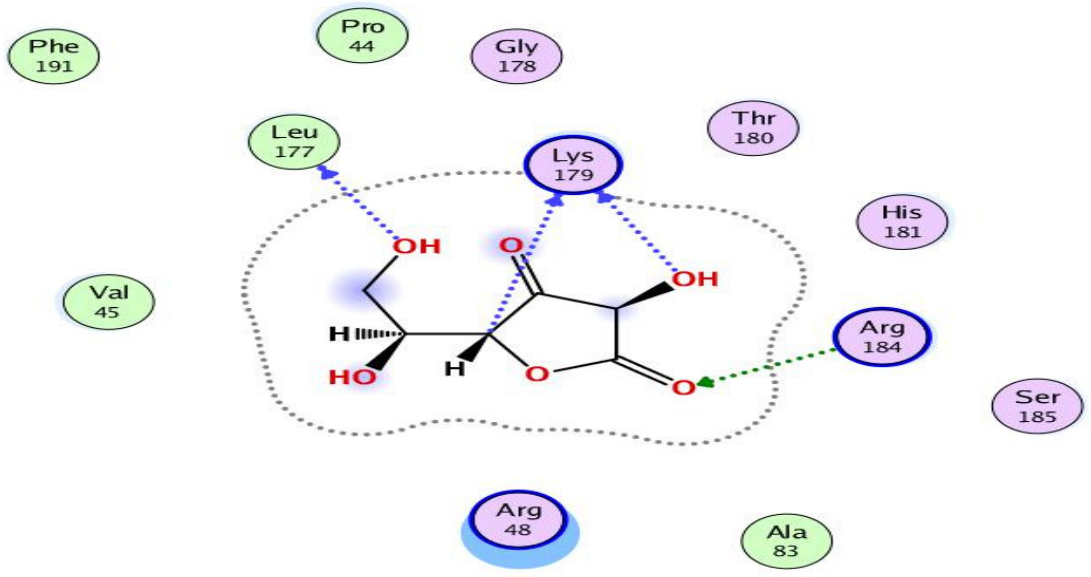

Figure S1. 2D interaction images of ascorbic acid with the active sites of 2X08.

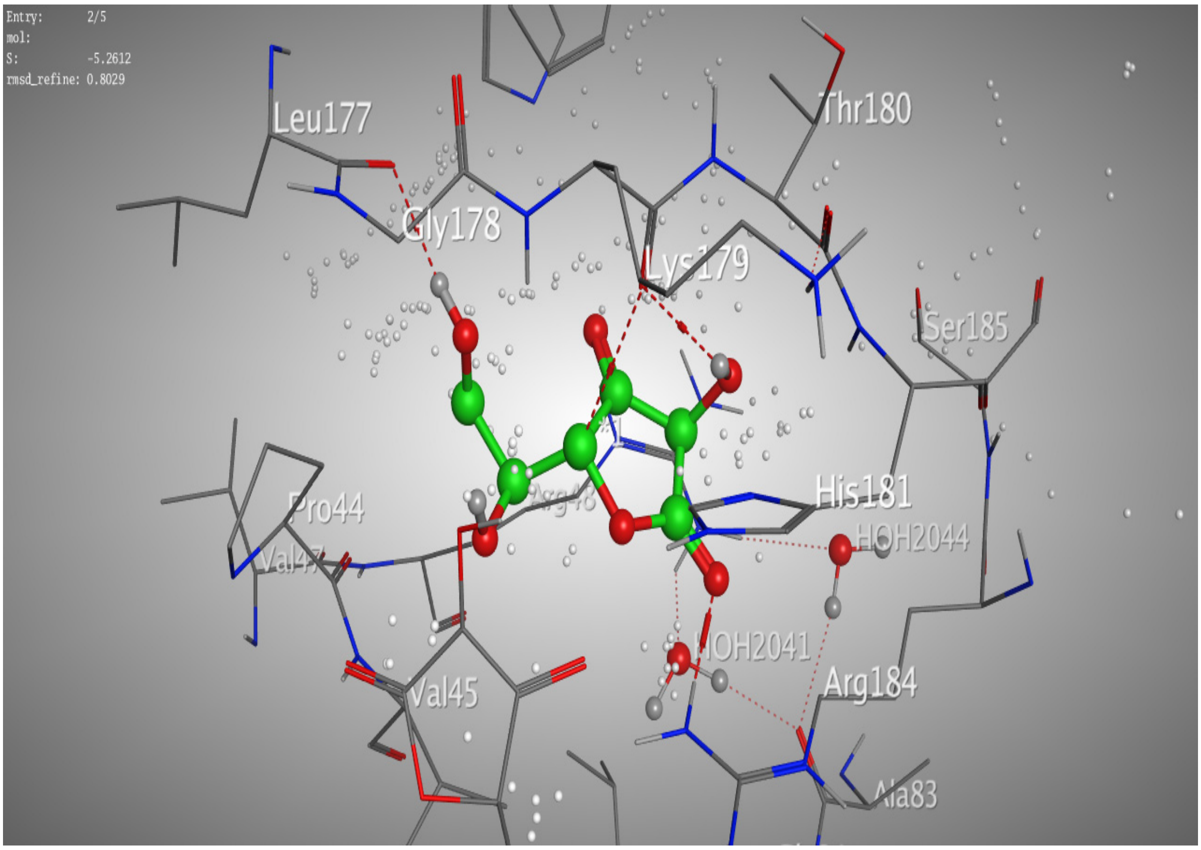

Figure S2. 3D interaction images of ascorbic acid with the active sites of 2X08.

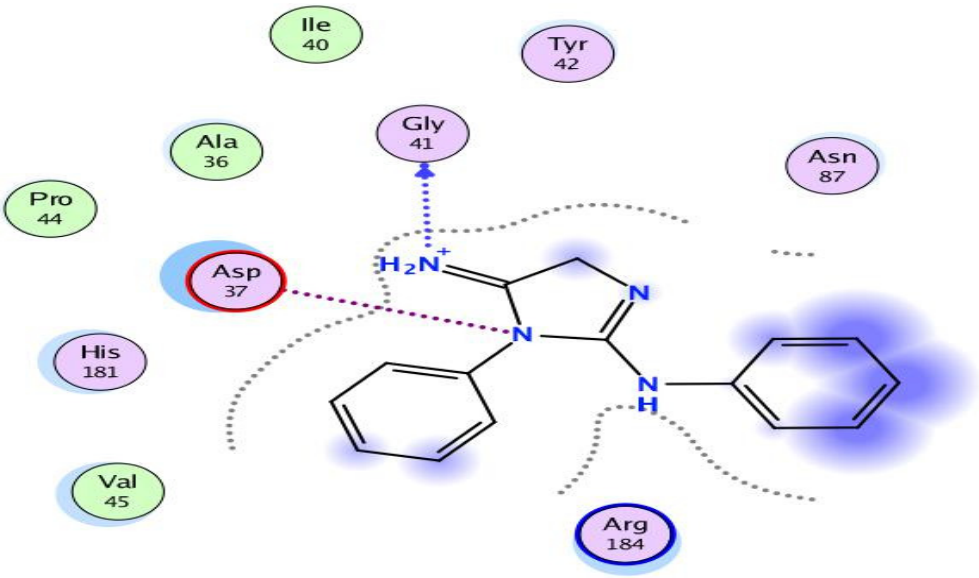

Figure S3. 2D interaction images of compound **2** with the active sites of 2X08.

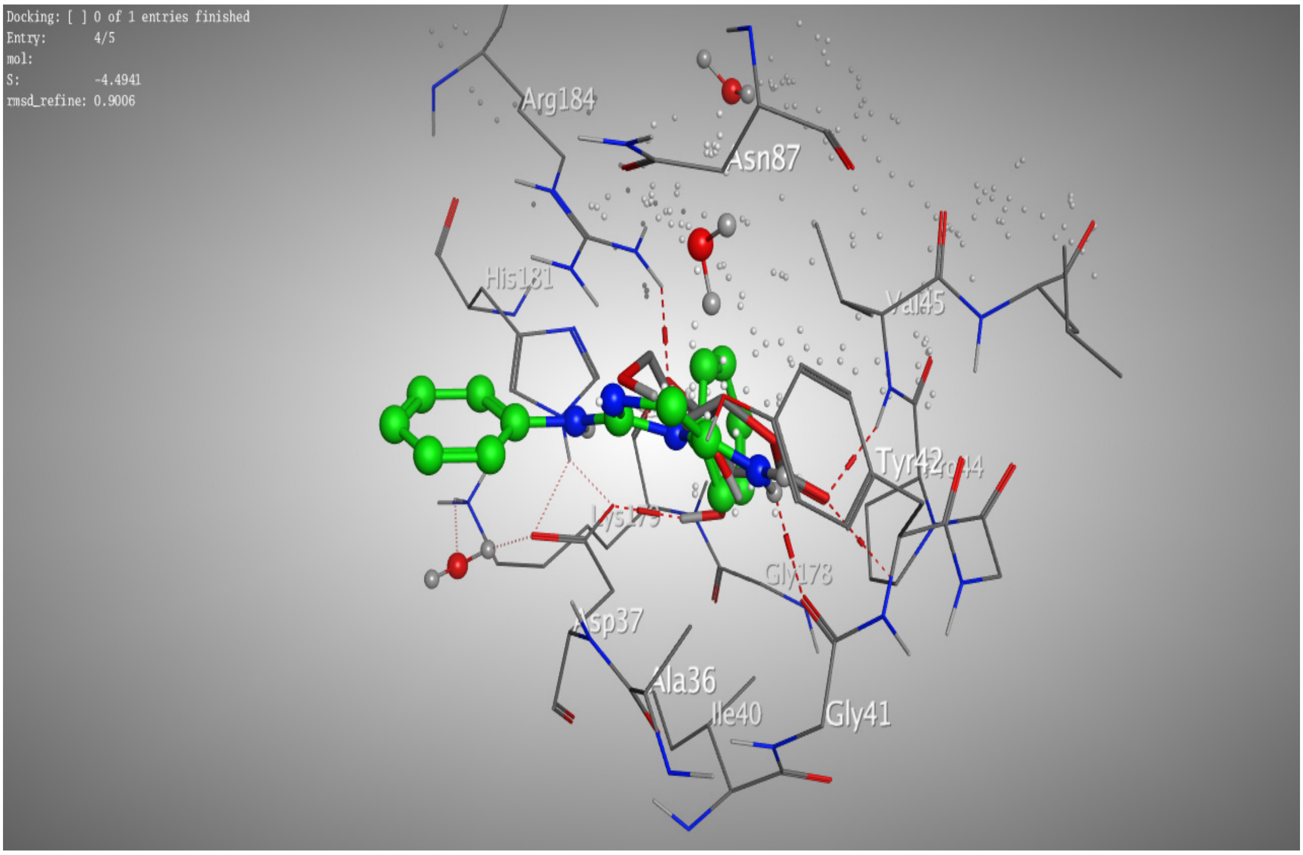

Figure S4. 3D interaction images of compound **2** with the active sites of 2X08.

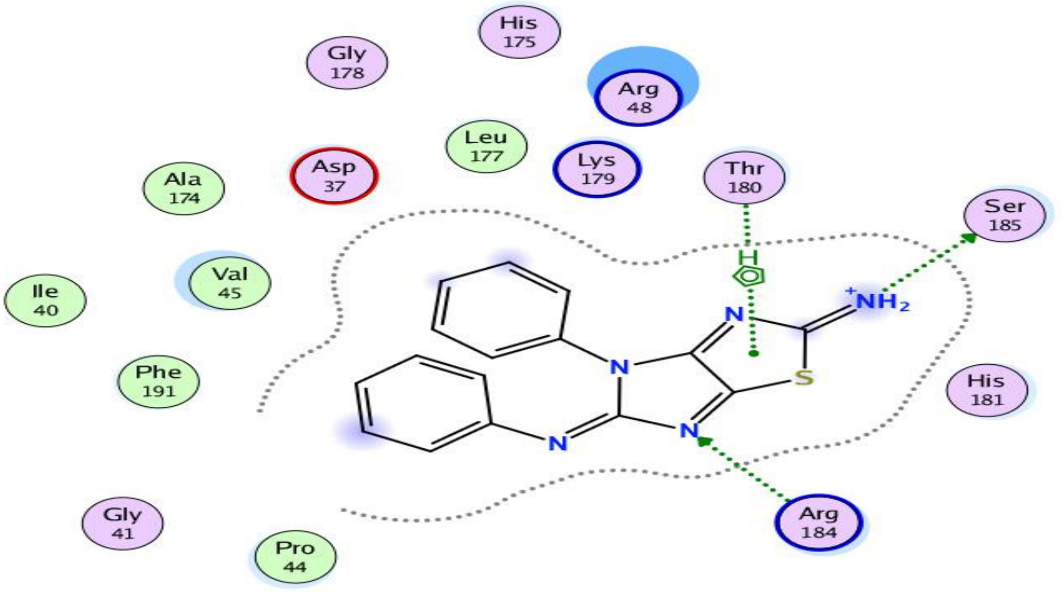

Figure S5. 2D interaction images of compound **3** with the active sites of 2X08.

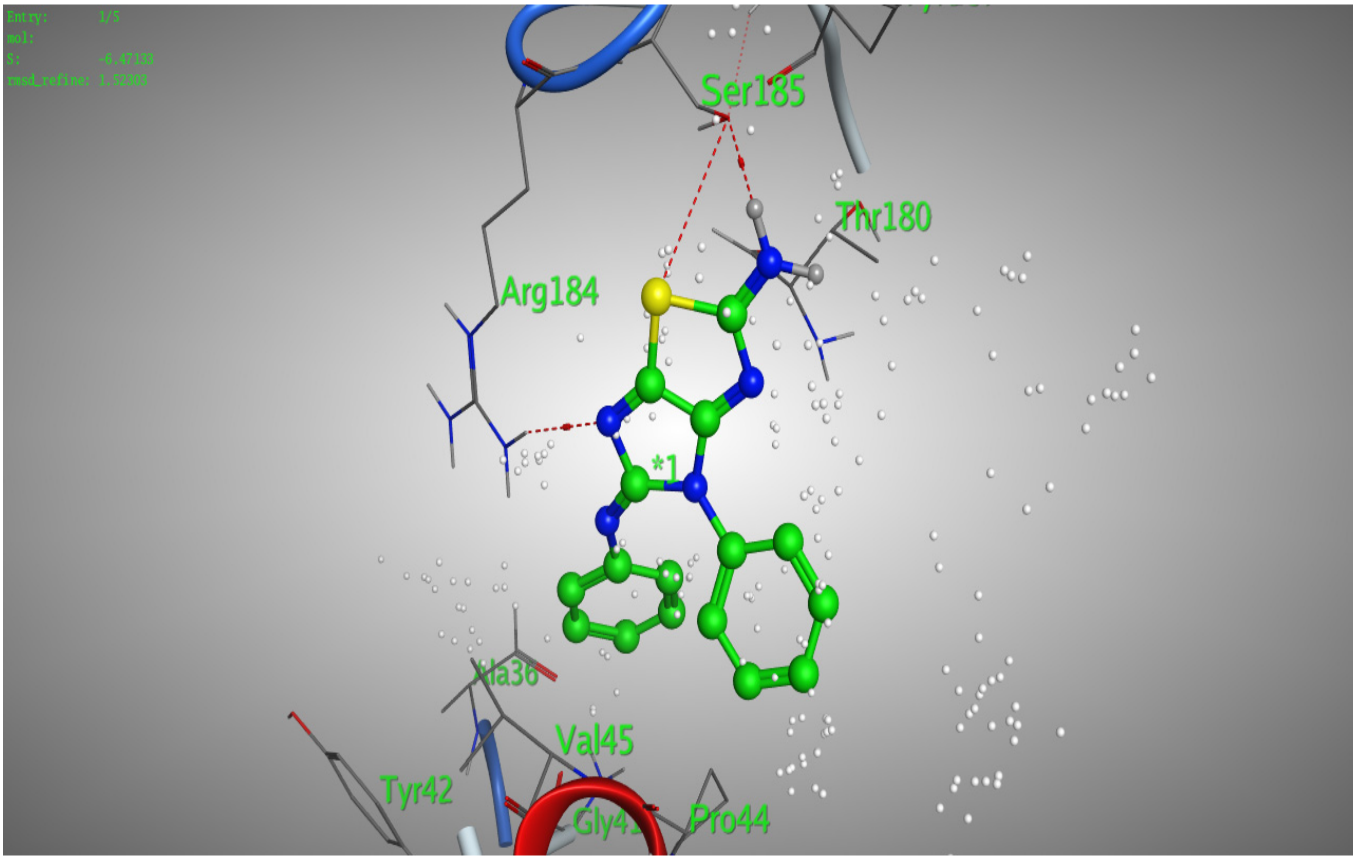

Figure S6. 3D interaction images of compound **3** with the active sites of 2X08.

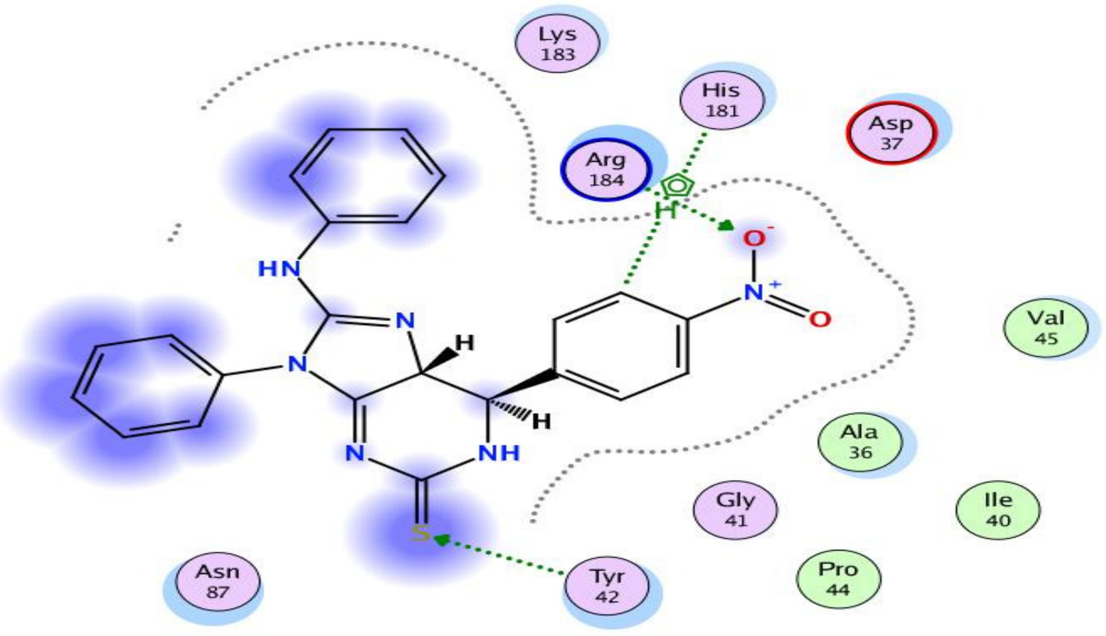

Figure S7. 2D interaction images of compound **5** with the active sites of 2X08.

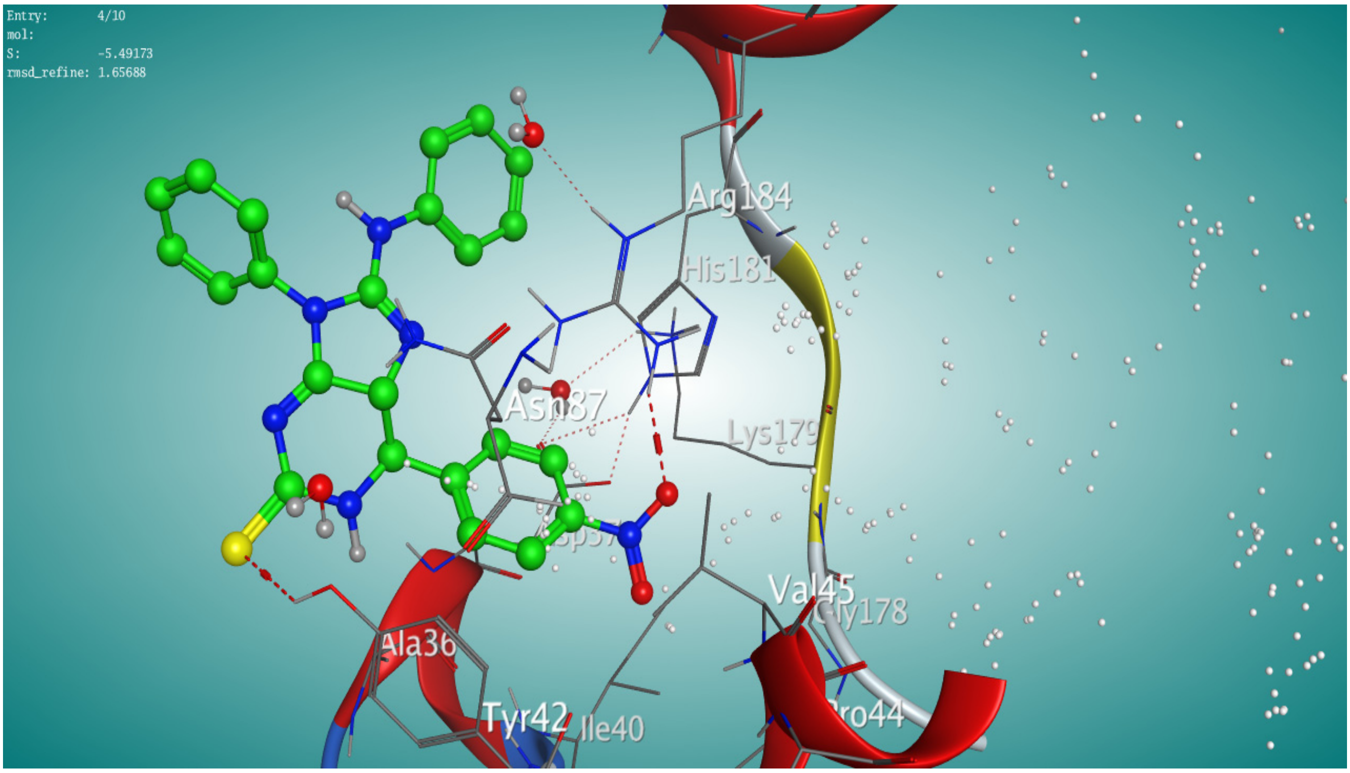

Figure S8. 3D interaction images of compound **5** with the active sites of 2X08.

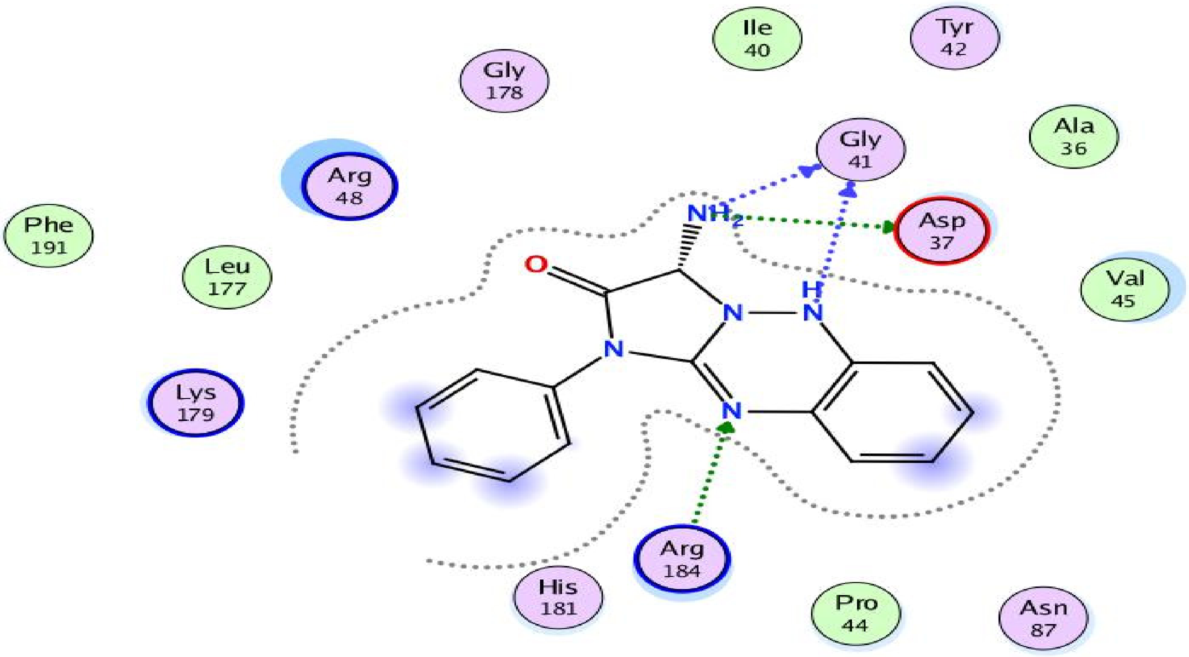

Figure S9. 2D interaction images of compound **6** with the active sites of 2X08.

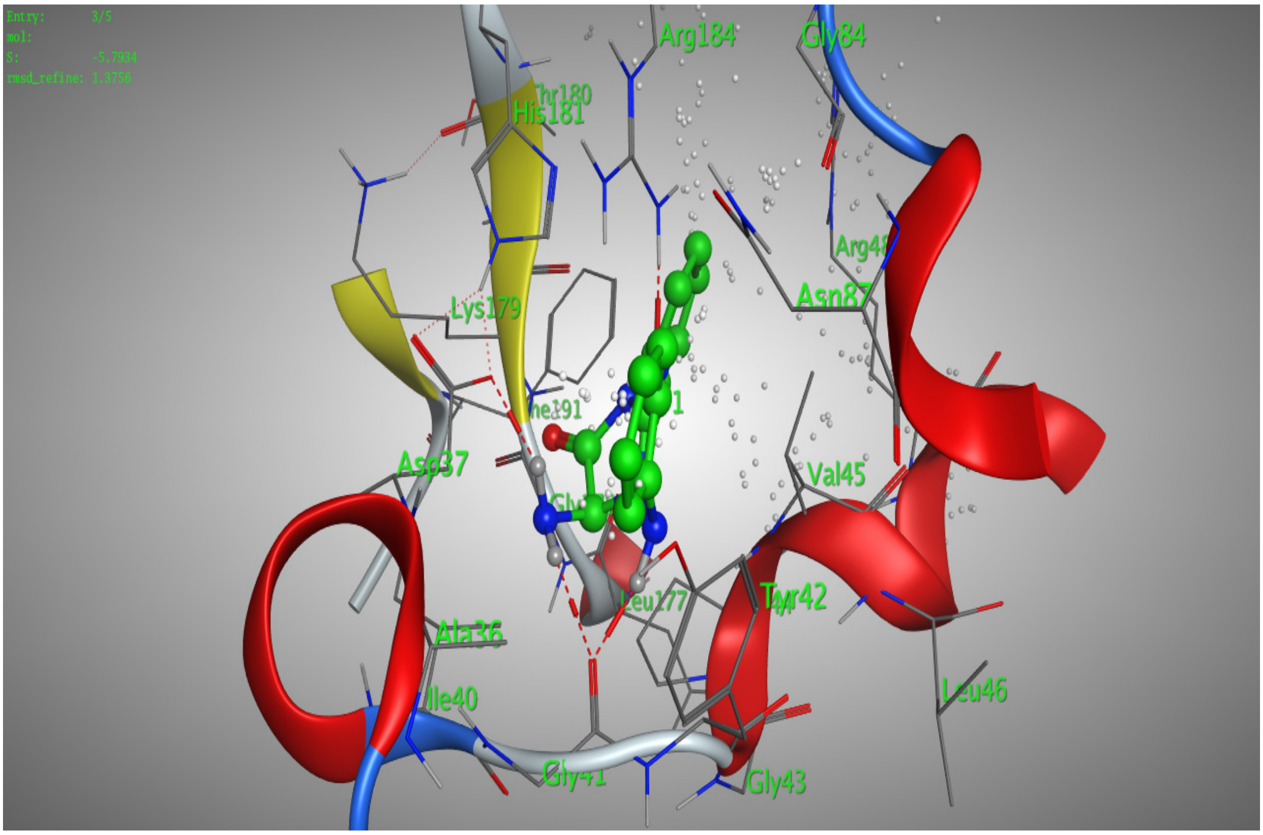

Figure S10. 3D interaction images of compound **6** with the active sites of 2X08.

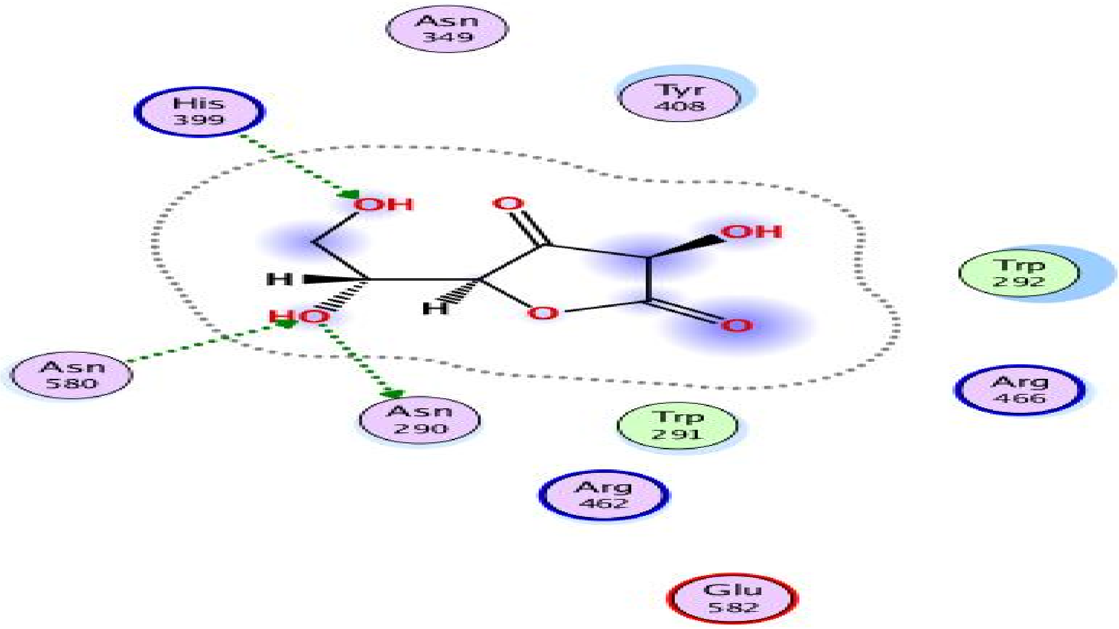

Figure S11. 2D interaction images of ascorbic acid with the active sites of 1F9G.

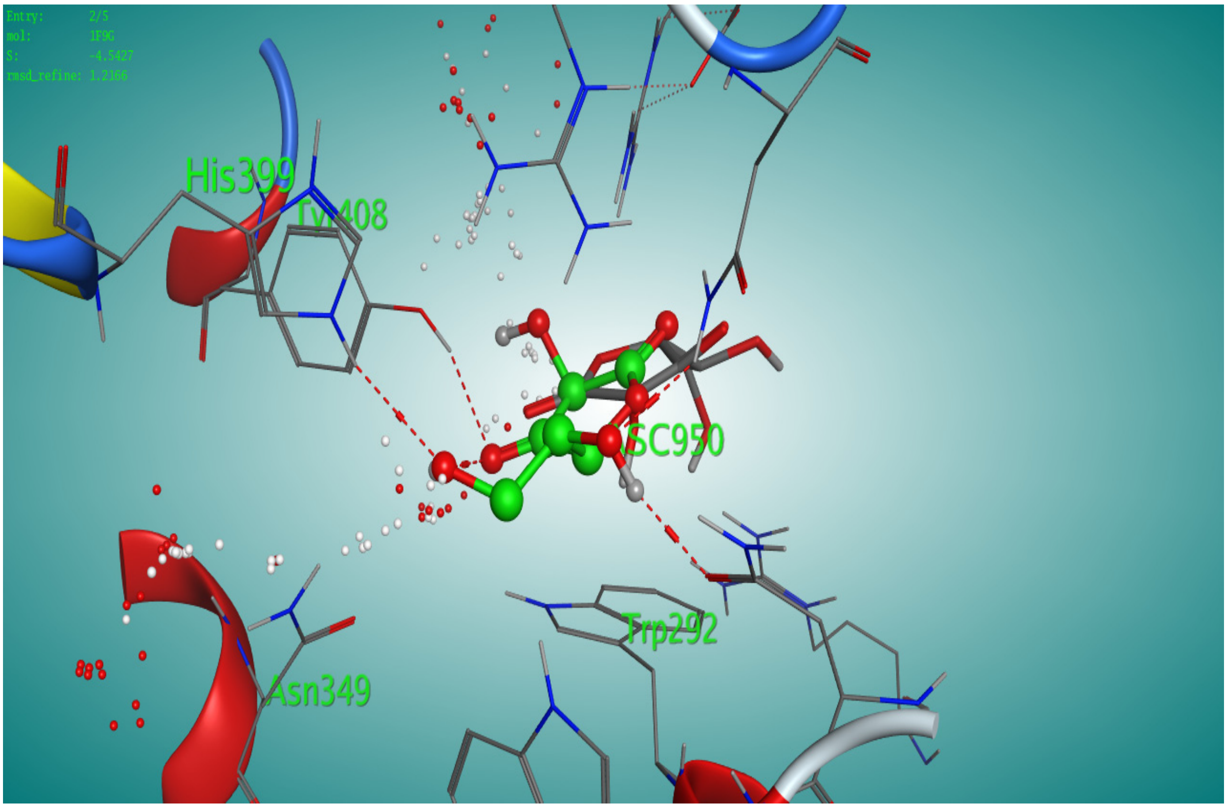

Figure S12. 3D interaction images of ascorbic acid with the active sites of 1F9G.

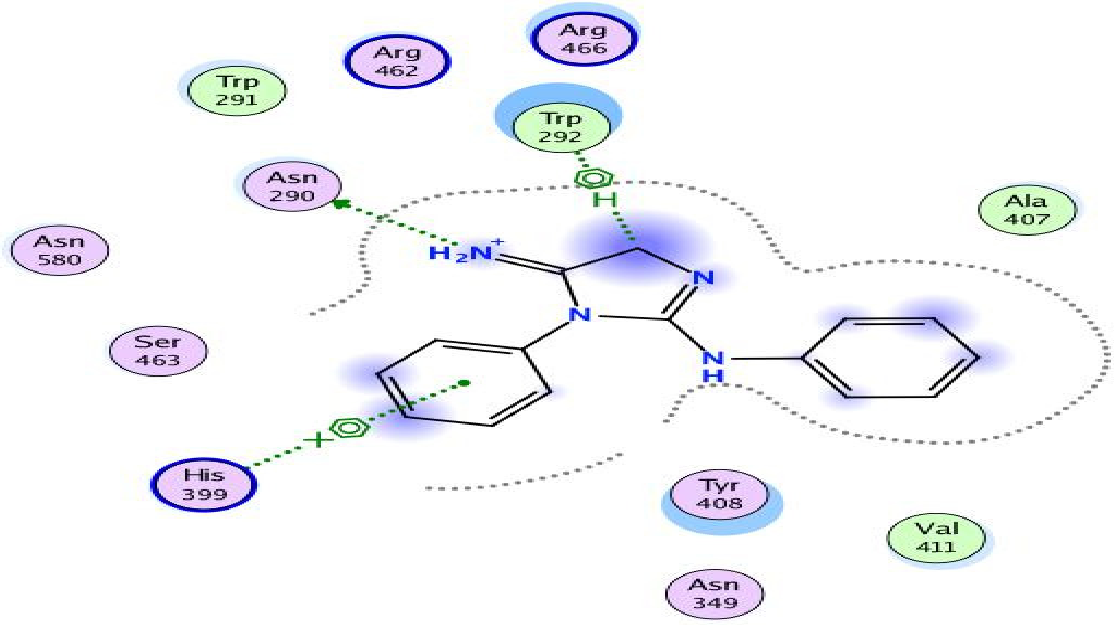

Figure S13. 2D interaction images of compound **2** with the active sites of 1F9G.

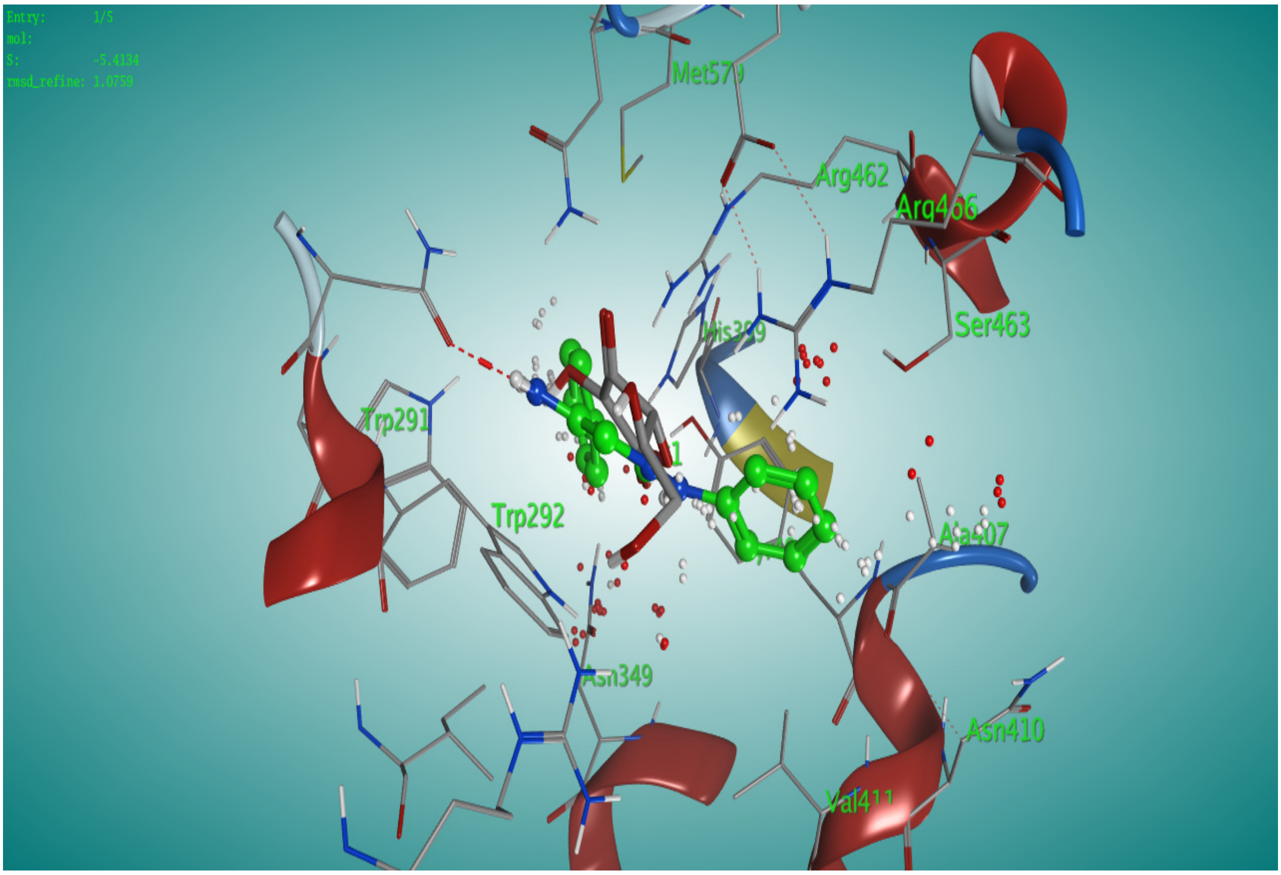

Figure S14. 3D interaction images of compound **2** with the active sites of 1F9G.

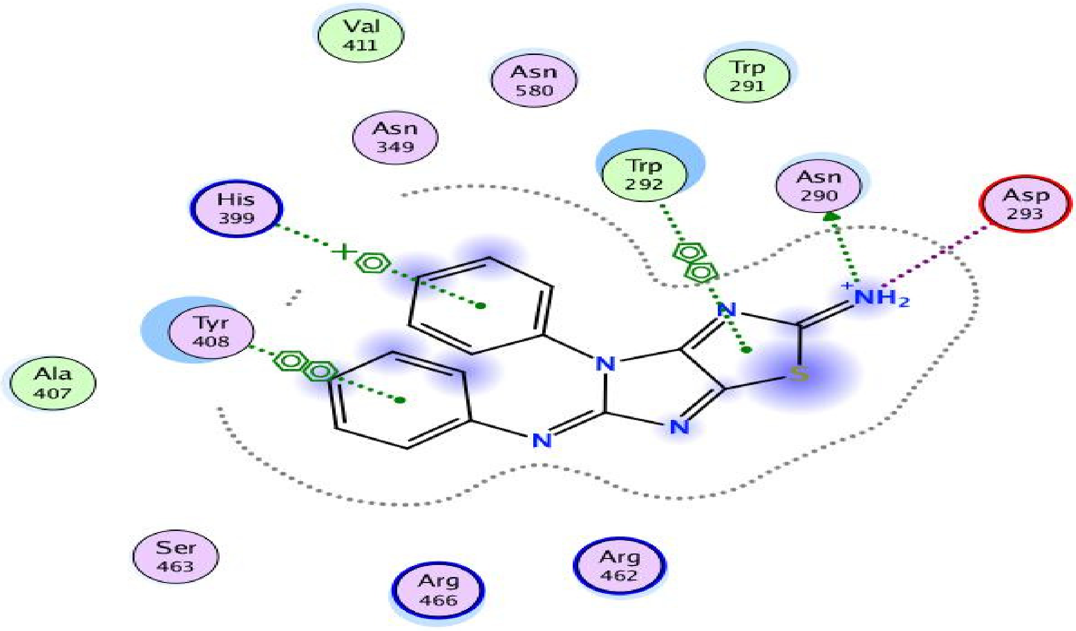

Figure S15. 2D interaction images of compound **3** with the active sites of 1F9G.

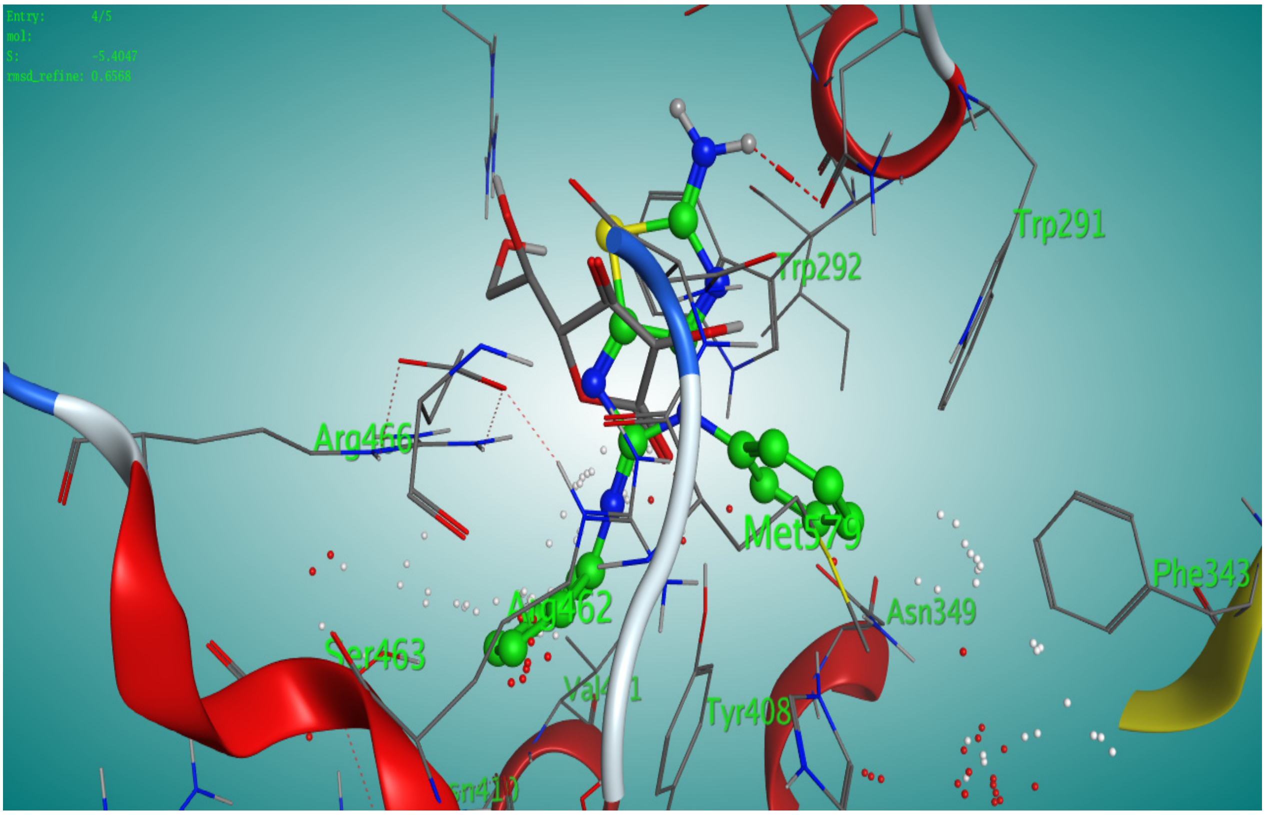

Figure S16. 3D interaction images of compound **3** with the active sites of 1F9G.

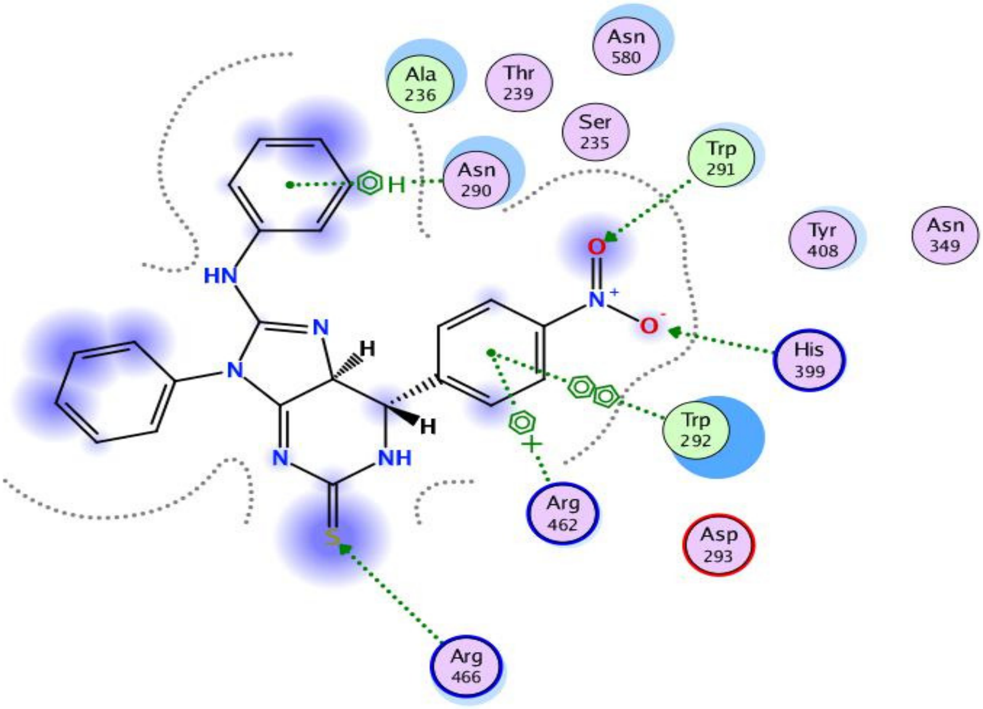

Figure S17. 2D interaction images of compound **5** with the active sites of 1F9G.

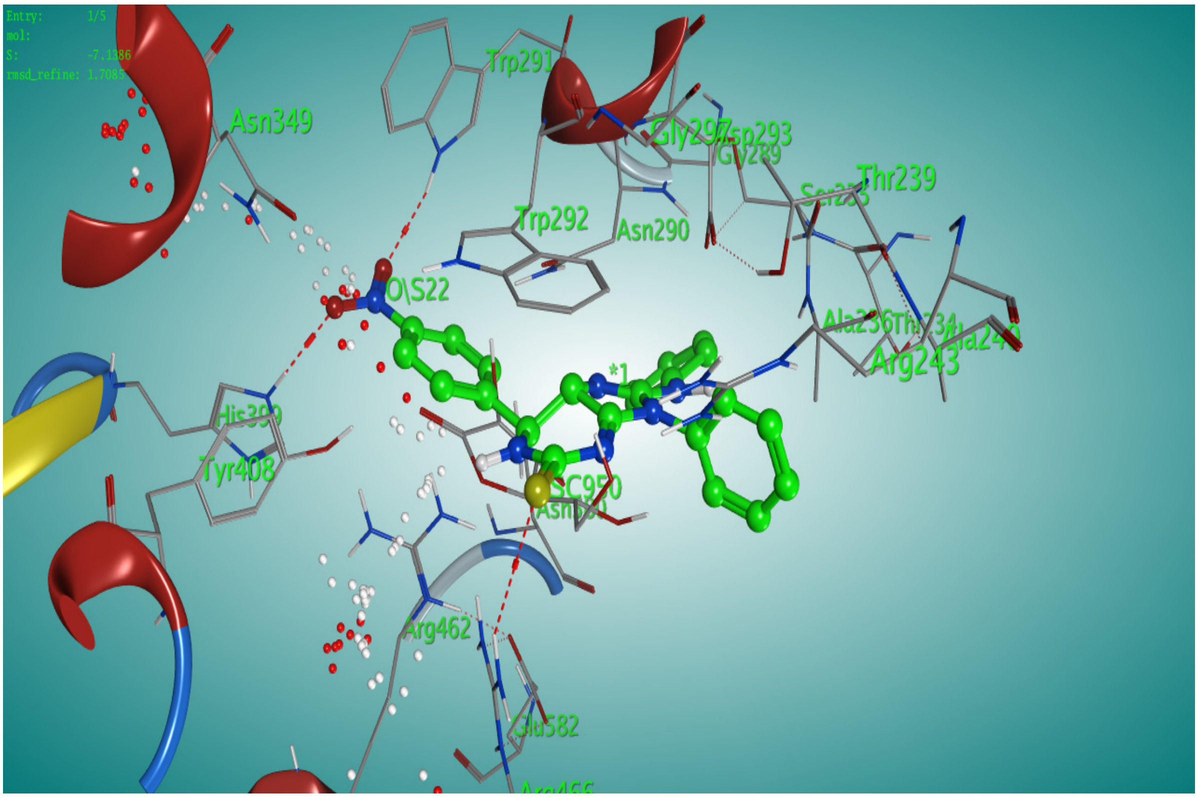

Figure S18. 3D interaction images of compound **5** with the active sites of 1F9G.

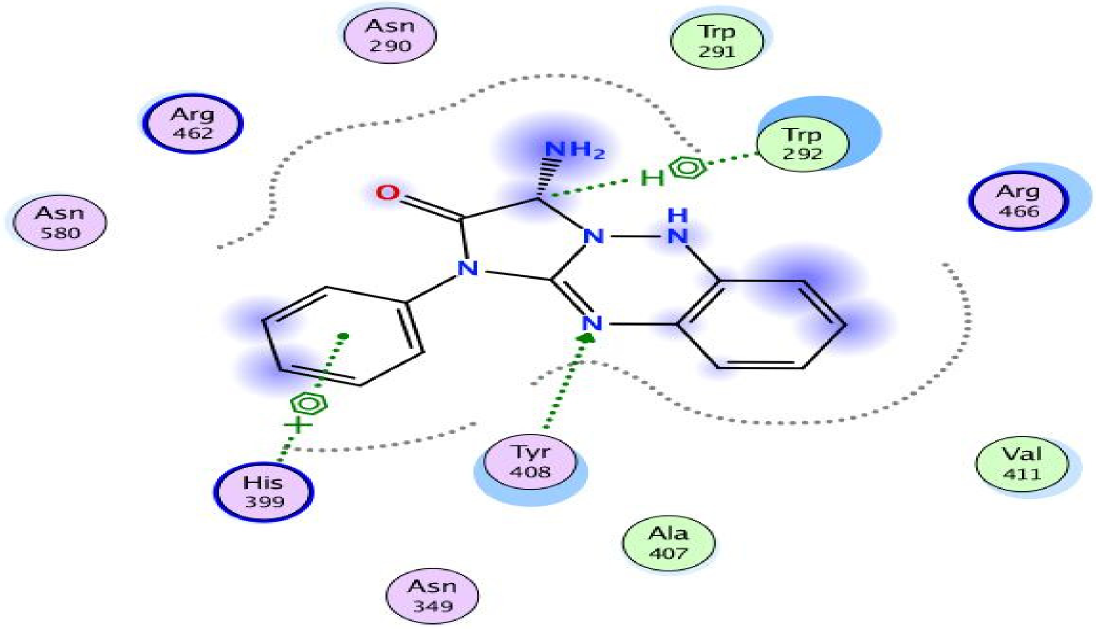

Figure S19. 2D interaction images of compound **6** with the active sites of 1F9G.

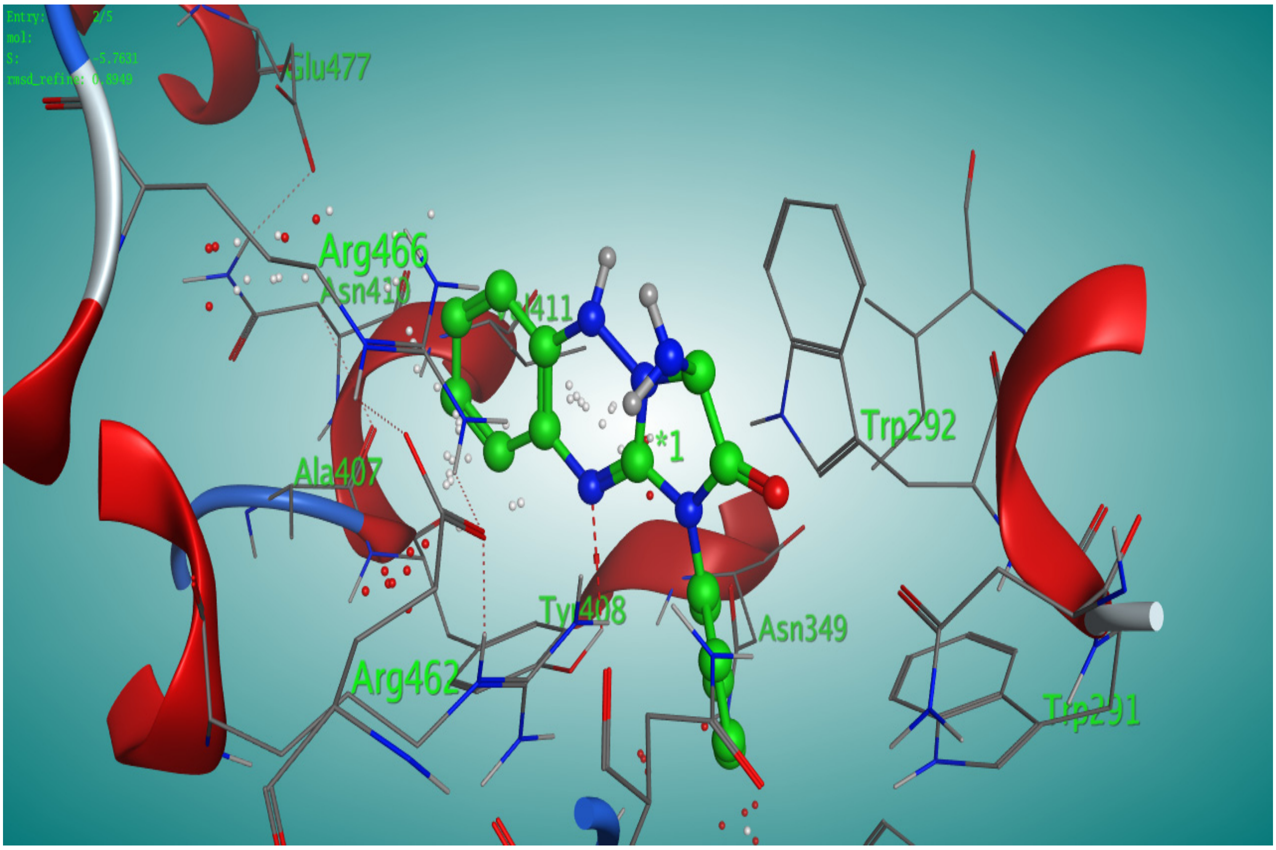

Figure S20. 3D interaction images of compound **6** with the active sites of 1F9G.

Chemistry

All chemicals were purchased from Sigma-Aldrich (Taufkirchen, Germany), and all solvents were purchased from El-Nasr Pharmaceutical Chemicals Company (analytical reagent grade, Egypt). All chemicals were used as supplied without further purification. The melting points were measured by a digital Electrothermal IA 9100 Series apparatus Cole-Parmer, Beacon Road, Stone, Staffordshire, ST15 OSA, UK) and were uncorrected. C, H, and N analyses were carried out on a PerkinElmer CHN 2400. IR spectra were recorded on FT-IR 460 PLUS (KBr disks) in the range from 4000 to 400 cm^{-1}. ^{1}H and ^{13}C-NMR spectra were recorded on a Bruker 400 MHz NMR Spectrometer using tetramethylsilane (TMS) as the internal standard, chemical shifts are expressed in δ (ppm), and DMSO-d6 was used as the solvent. At the Regional Centre for Mycology & Biotechnology (RCMB) Al-Azhar University, Naser City, Cairo.

Data analysis

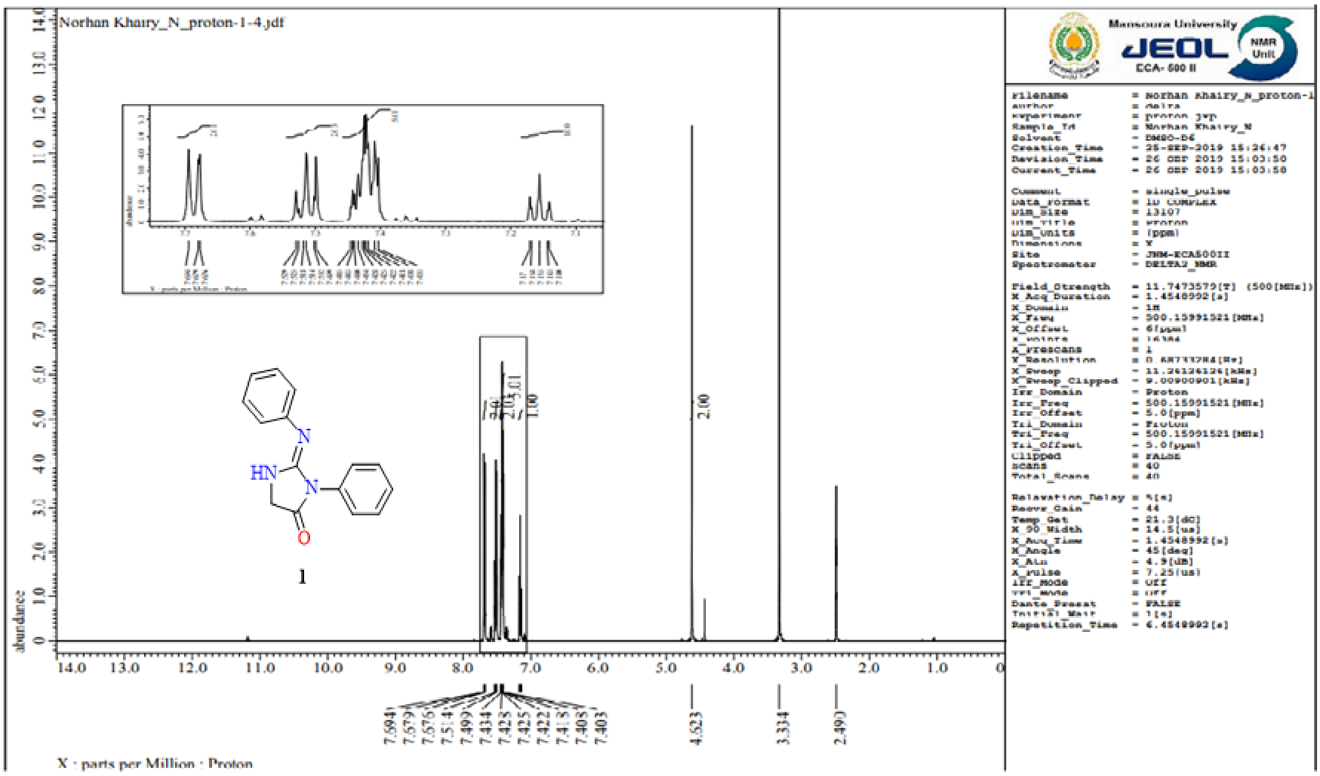

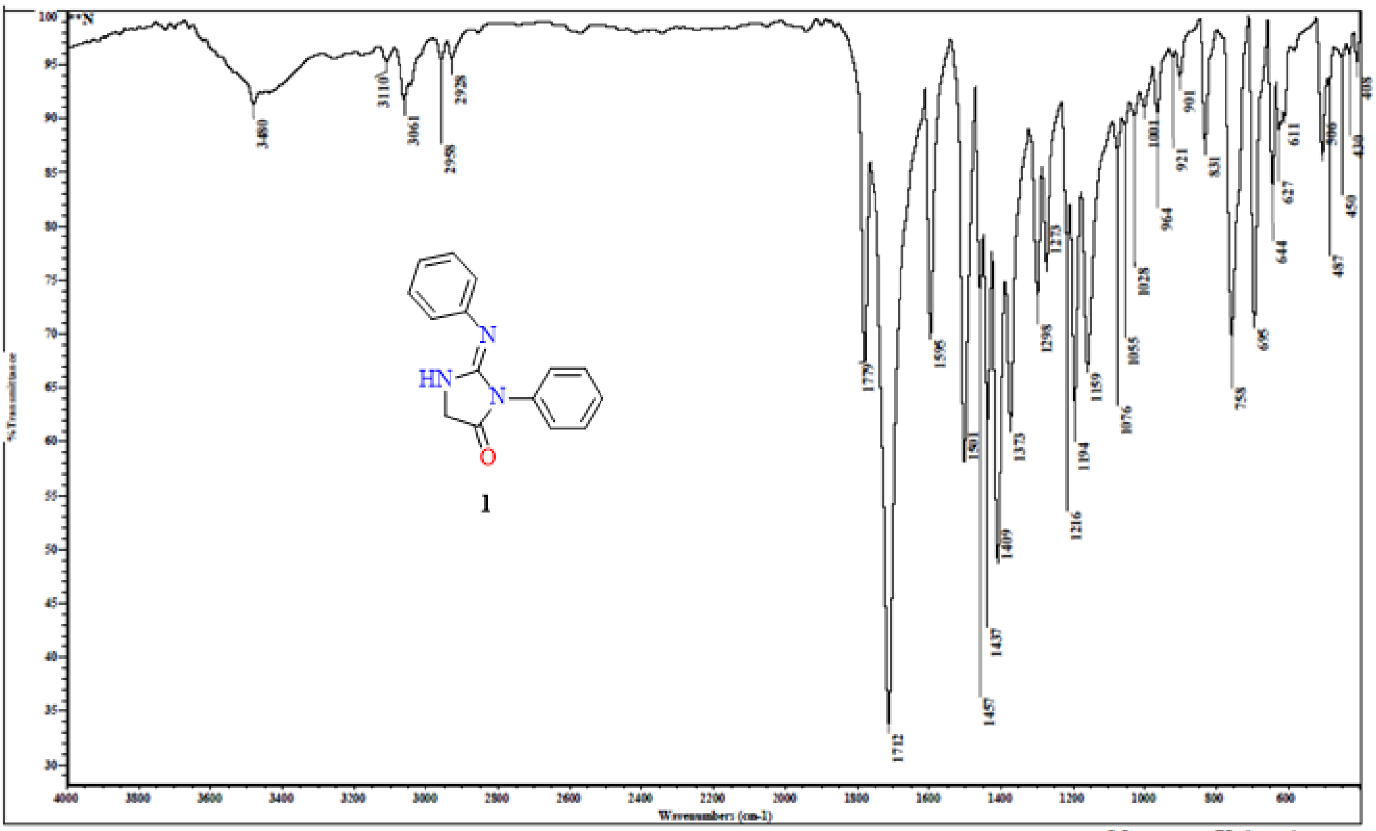

Number of sample scans: 32
Number of background scans: 32
Resolution: 4.000
Sample gain: 2.0
Optical velocity: 0.4747
Aperture: 80.00

Thermo Fisher SCIENTIFIC
Thu Sep 12 09:40:01 2019 (GMT+02:00)

Mansoura University
Faculty of Science
Spectral Analysis Unit
unitofspectra@gmail.com

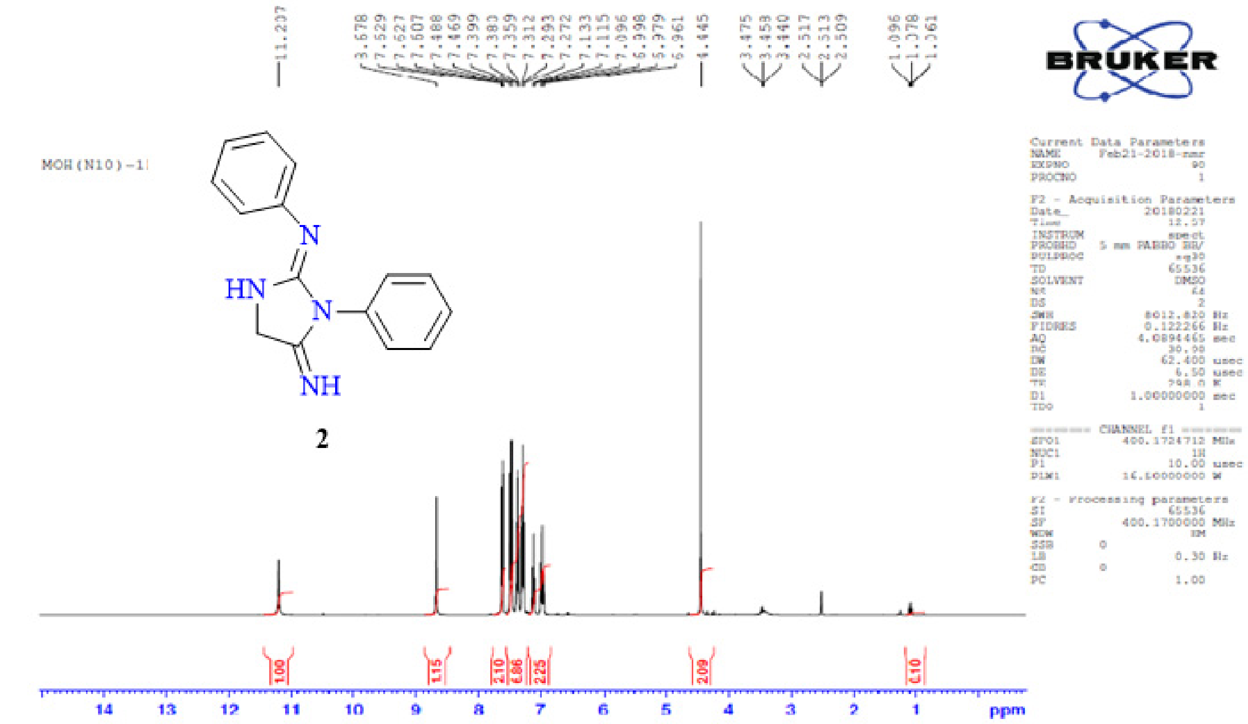

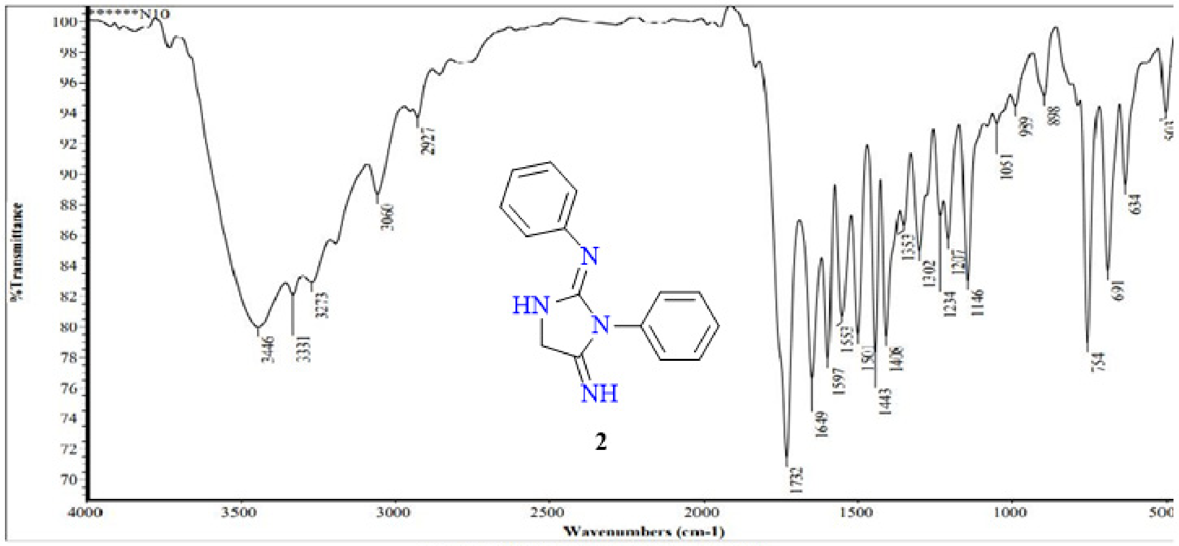

******N10
Number of sample scans: 32
Number of background scans: 32
Resolution: 4.000
Sample gain: 2.0
Optical velocity: 0.4747
Aperture: 80.00

Sat Mar 10 08:58:46 2018 (GMT-08:00)

ThermoFisher SCIENTIFIC

Mansoura University
Faculty of Science
Spectral Analyses unit
Chemistry Department
ThermoFisher Nicolete IS10, USA
Spectral range: 4000 - 400 cm-1

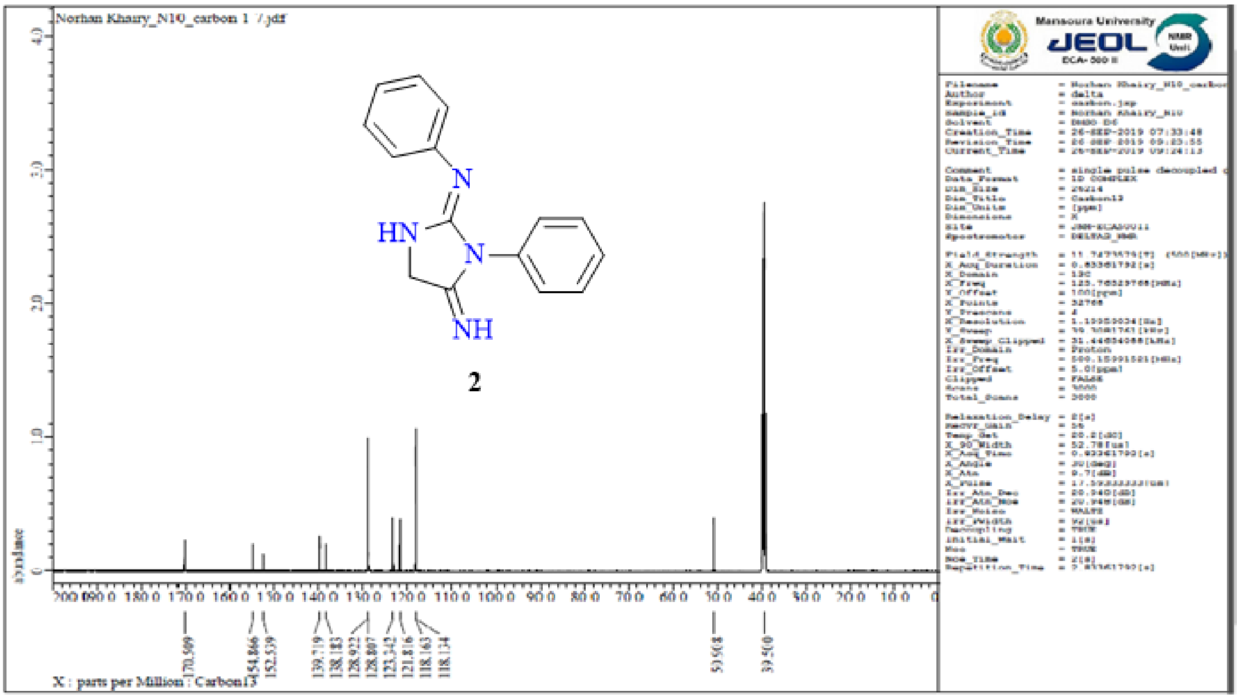
HN
N
N
NH
2
Mansoura University
JEOL
ECA- 500 II
170.509
154.866
152.539
139.719
138.183
128.922
128.807
123.342
121.816
118.163
118.134
50.508
39.500
X : parts per Million : Carbon13

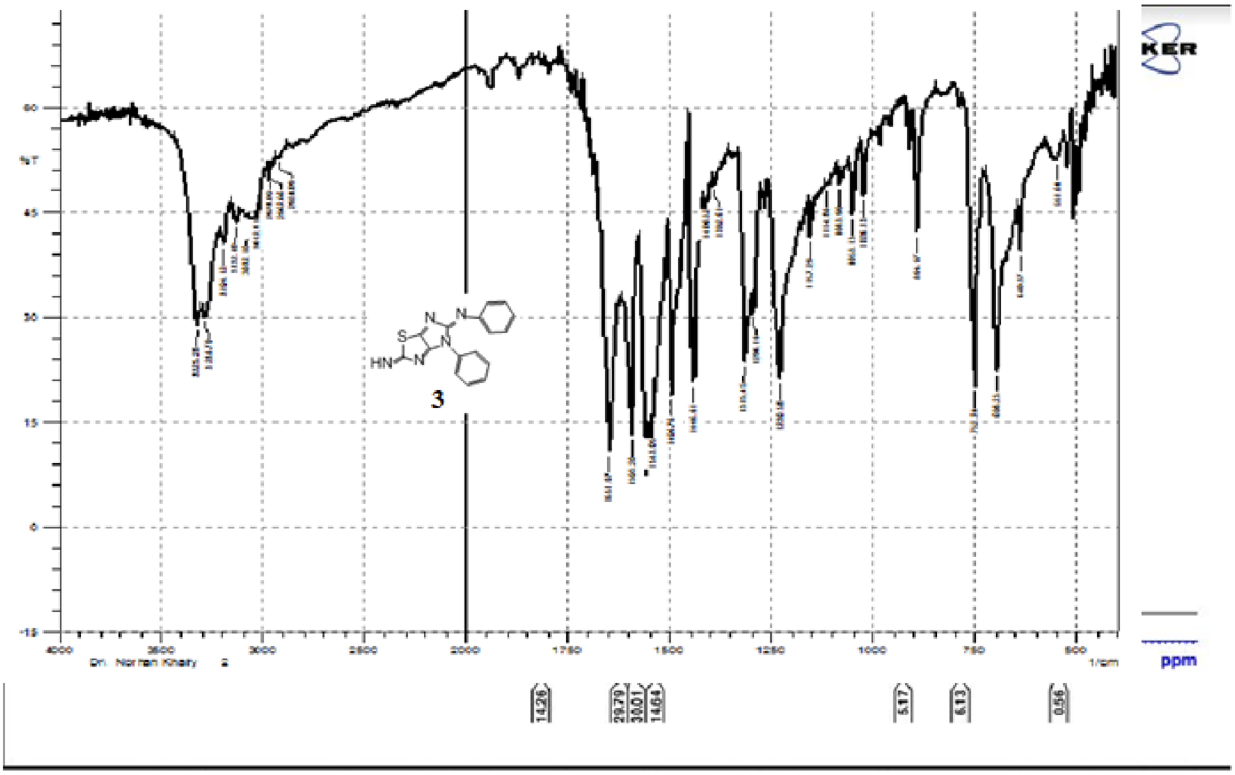
%T
1/cm
3
ppm

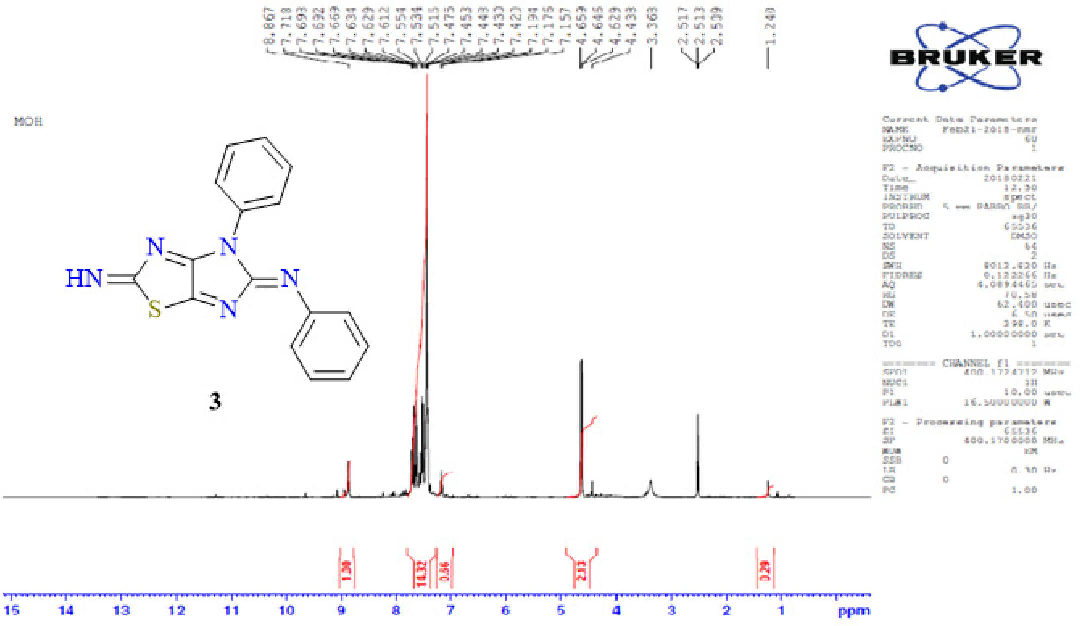
BRUKER
8.867
7.713
7.693
7.692
7.669
7.634
7.629
7.612
7.554
7.534
7.515
7.475
7.453
7.443
7.433
7.420
7.194
7.175
7.157
4.659
4.645
4.629
4.433
3.363
2.517
2.513
2.509
1.240
HN
N
S
3
1.20
14.32
3.56
2.13
0.29
ppm

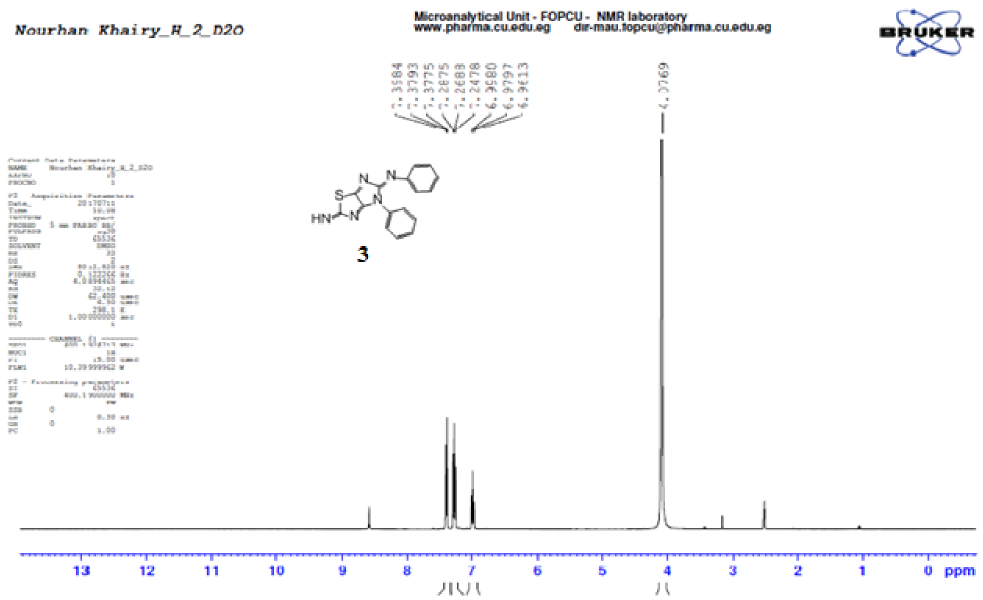

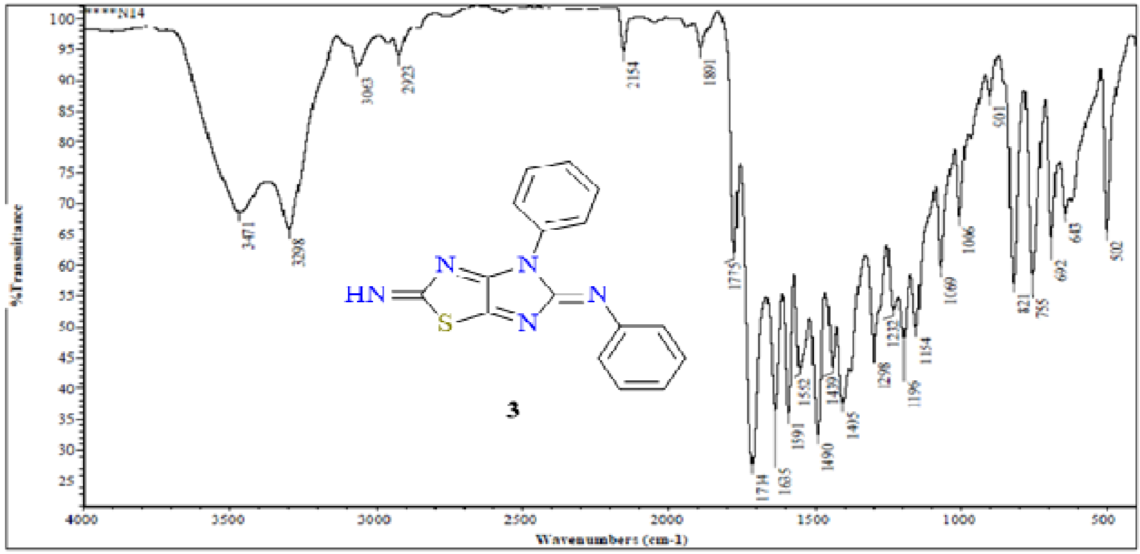

****N14
Number of sample scans: 32
Number of background scans: 32
Resolution: 4.000
Sample gain: 4.0
Optical velocity: 0.4747
Aperture: 80.00

Sat Mar 10 09:02:46 2018 (GMT-08:00)

ThermoFisher SCIENTIFIC

Mansoura University
Faculty of Science
Spectral Analyses unit
Chemistry Department
ThermoFisher Nicolete IS10, USA
Spectral range: 4000 - 400 cm-1

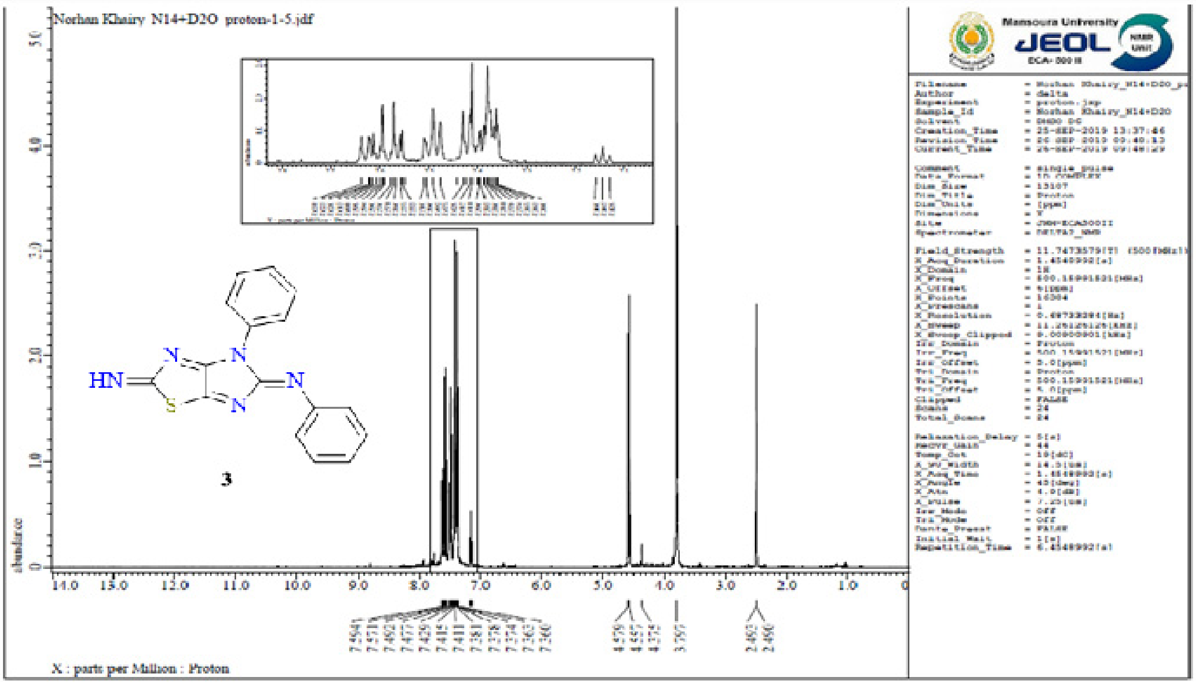

Norhan Khairy N14+D2O proton-1-5.jdf
HN
N
N
N
S
N
3
abundance
X : parts per Million : Proton
14.0
13.0
12.0
11.0
10.0
9.0
8.0
7.0
6.0
5.0
4.0
3.0
2.0
1.0
7.494
7.451
7.462
7.477
7.429
7.418
7.411
7.381
7.378
7.374
7.363
7.360
4.579
4.557
4.374
3.797
2.493
2.490
Mansoura University
JEOL
NMR Unit
ECA-500 II

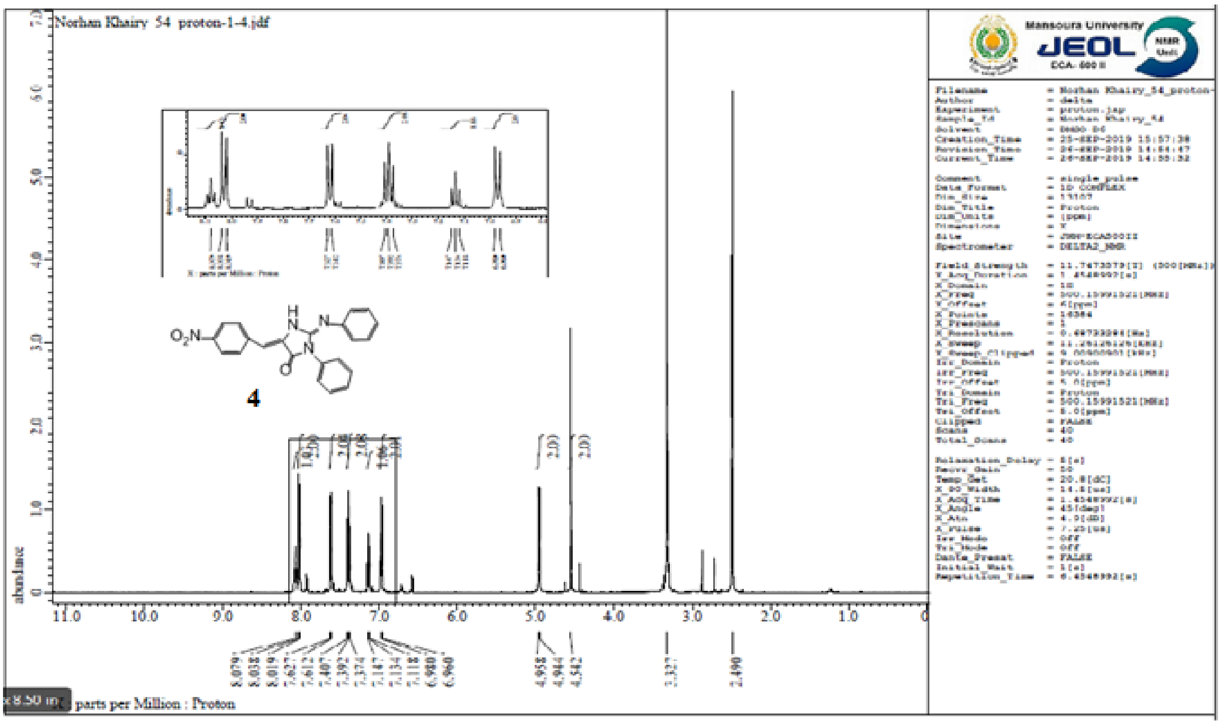

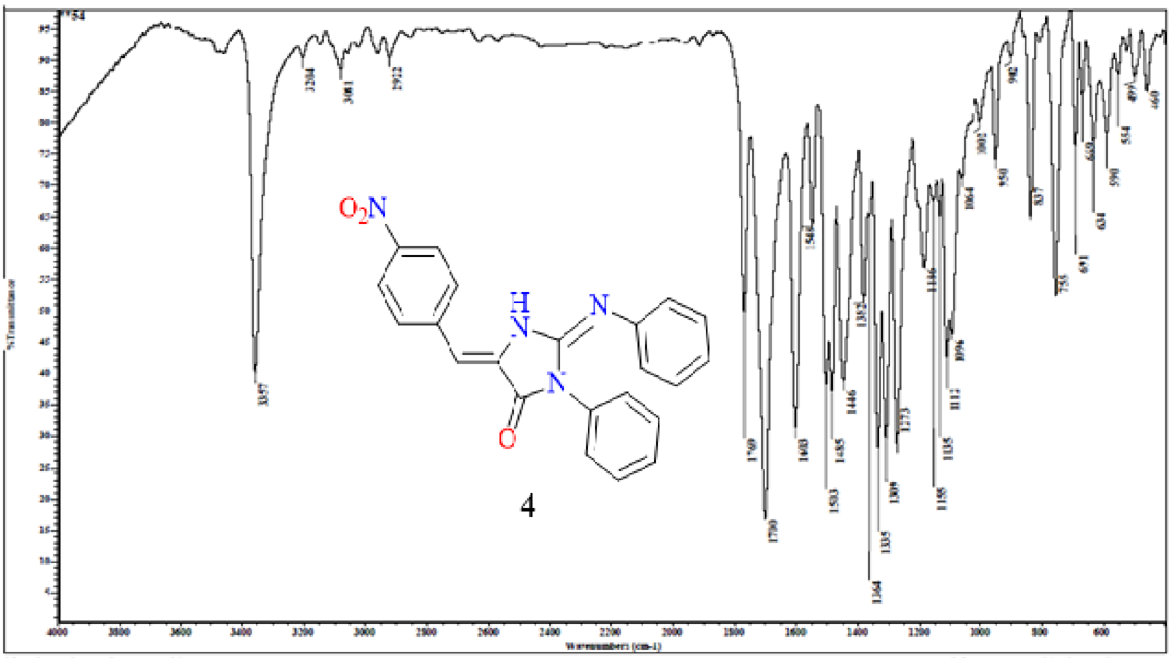

Number of sample scans: 32
Number of background scans: 32
Resolution: 4.000
Sample gain: 2.0
Optical velocity: 0.4747
Aperture: 80.00

ThermoFisher SCIENTIFIC
Thu Sep 12 09:39:34 2019 (GMT+02:00)

Mansoura University
Faculty of Science
Spectral Analysis Unit
unitofspectra@gmail.com

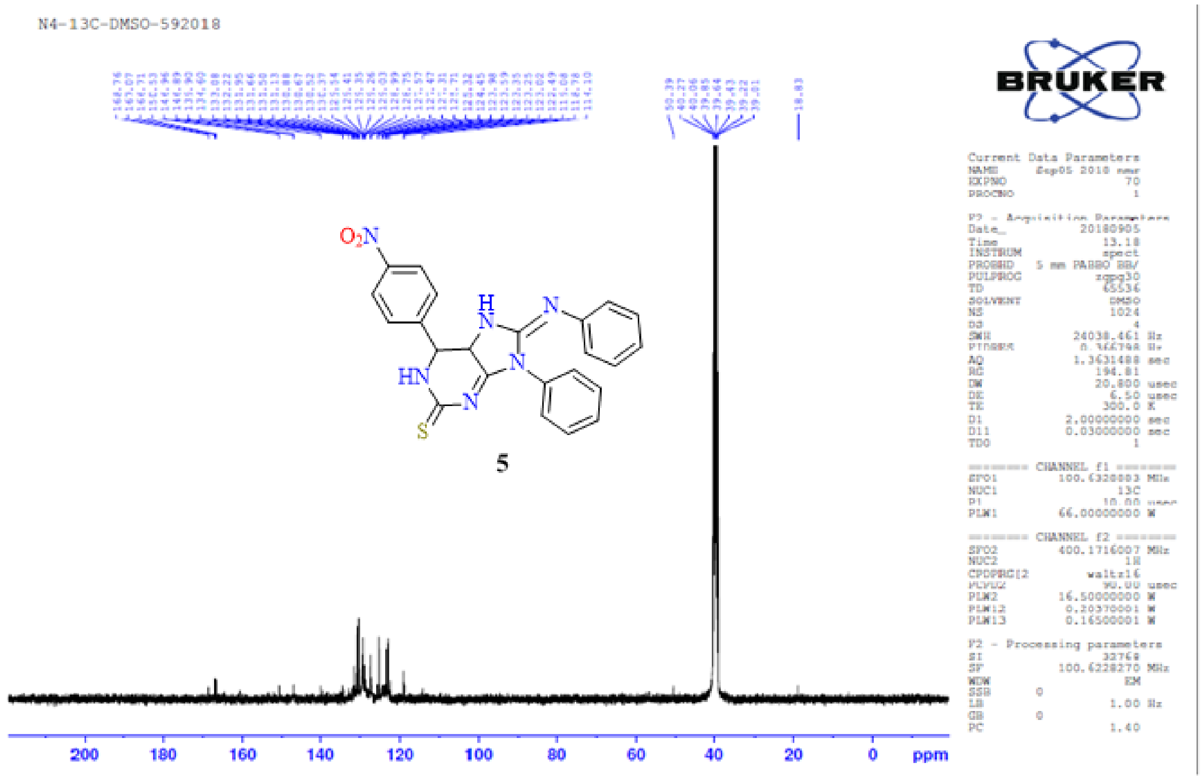
N4-13C-DMSO-592018
BRUKER
O2N
HN
N
H
N
N
S
5
200
180
160
140
120
100
80
60
40
20
0
ppm

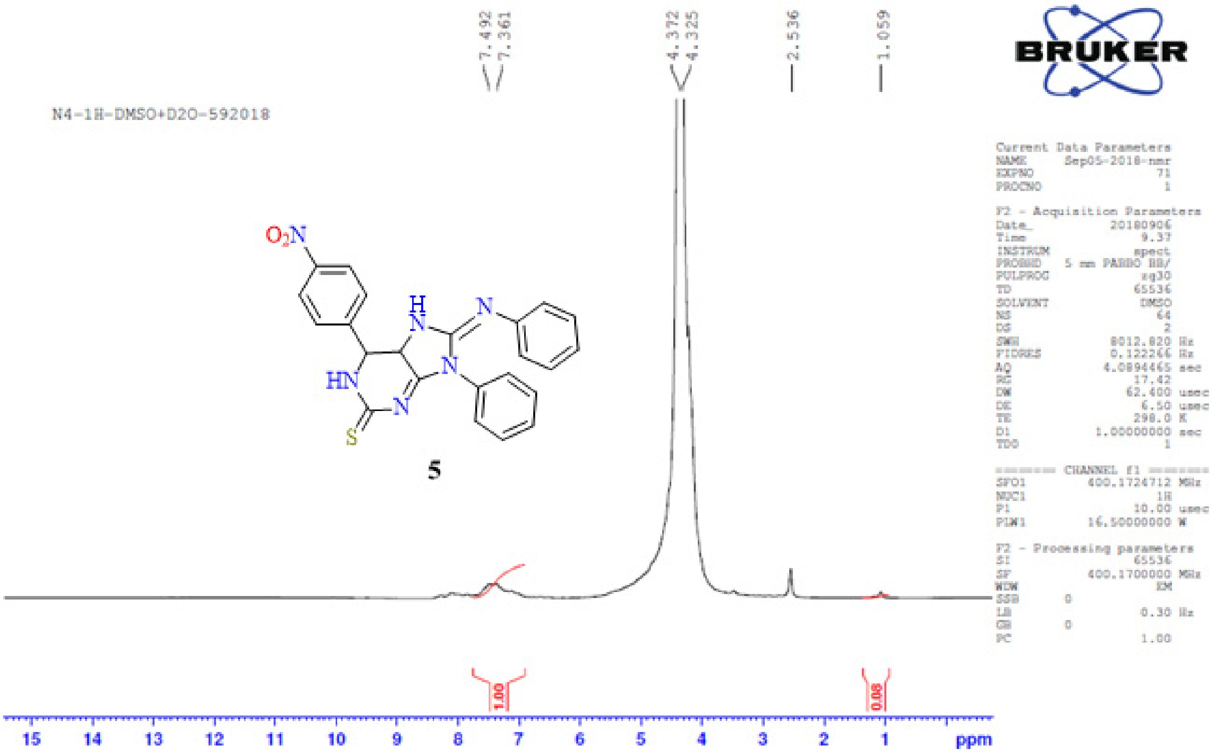
N4-1H-DMSO+D2O-592018
7.492
7.361
4.372
4.325
2.536
1.059
BRUKER
O2N
HN
N
H
N
N
S
5
1.00
0.08
15
14
13
12
11
10
9
8
7
6
5
4
3
2
1
ppm

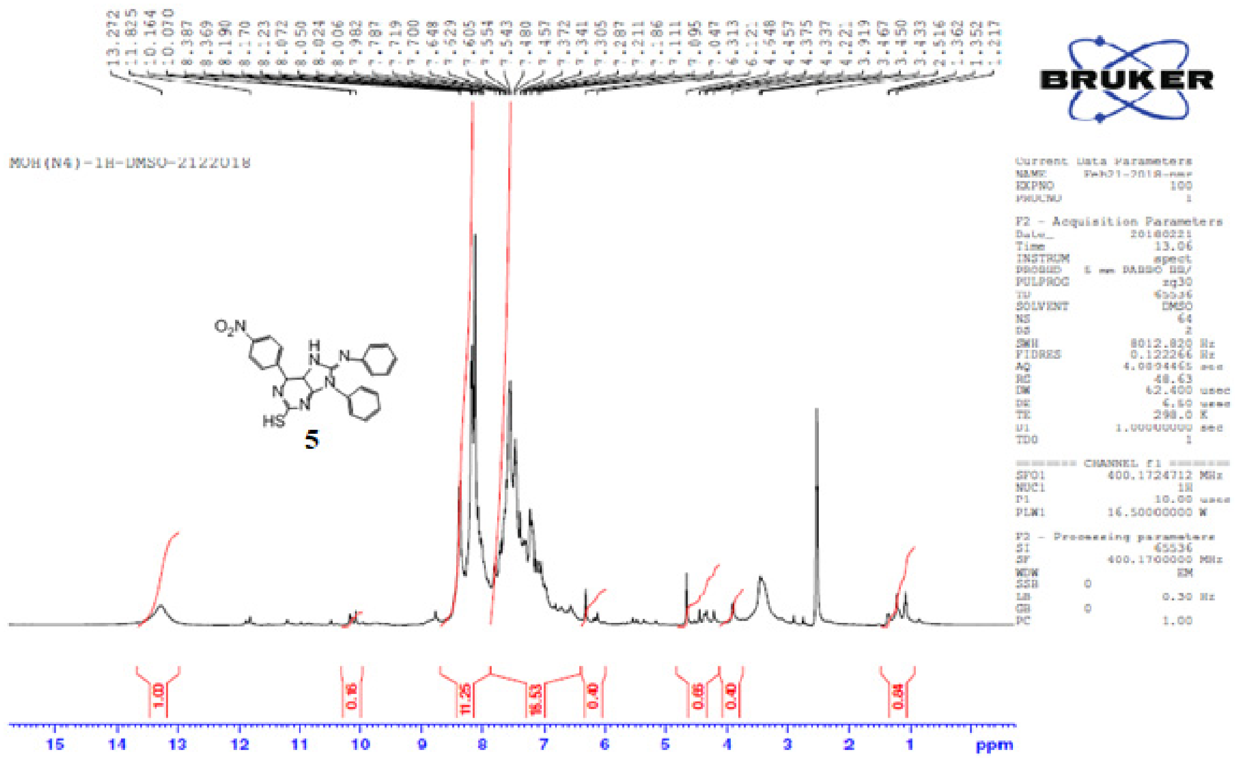

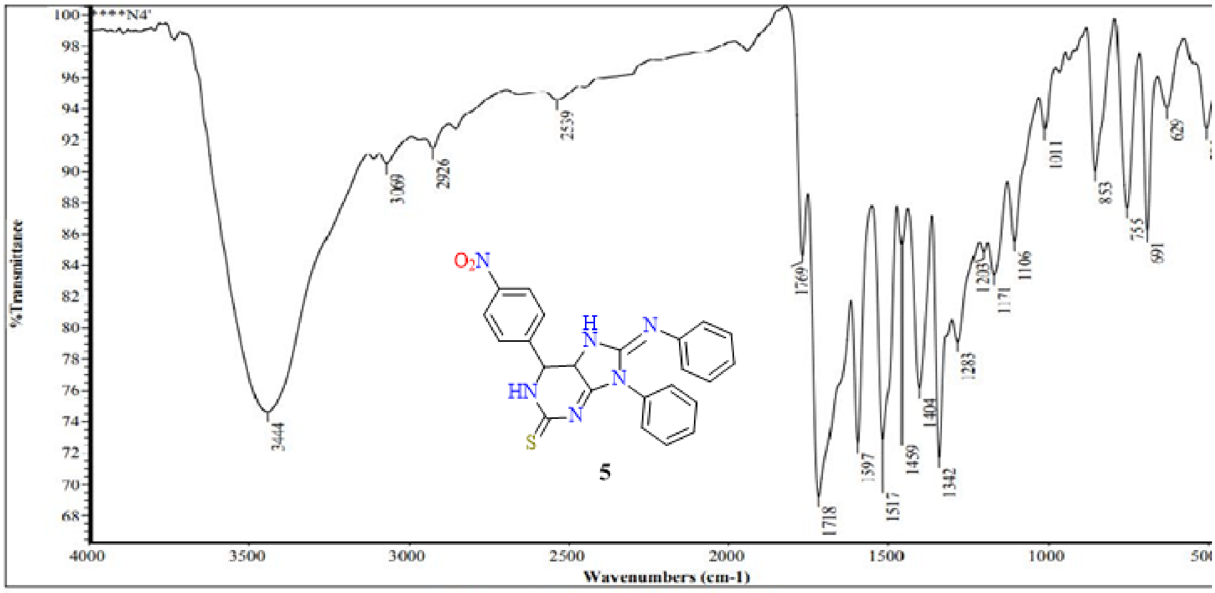

Sat Mar 10 09:00:11 2018 (GMT-08:00)

****N4'
Number of sample scans: 32
Number of background scans: 32
Resolution: 4.000
Sample gain: 2.0
Optical velocity: 0.4747
Aperture: 80.00

Mansoura University
Faculty of Science
Spectral Analyses unit
Chemistry Department
ThermoFisher Nicolete IS10, US.
Spectral range: 4000 - 400 cm-1

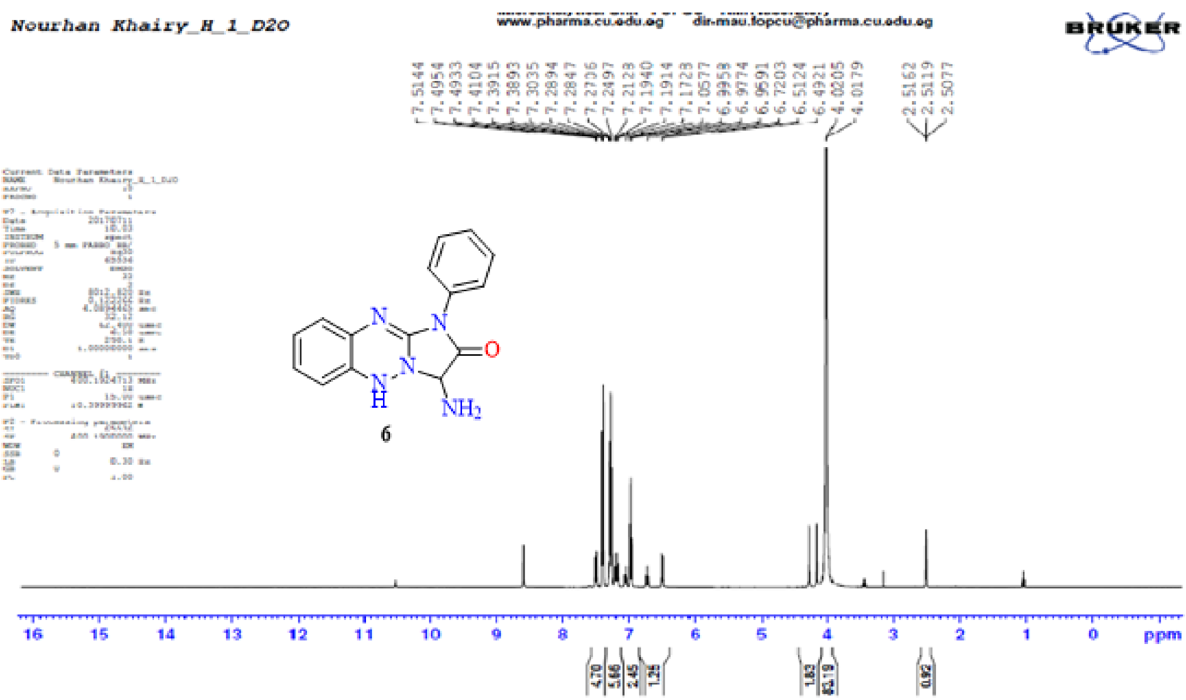
Nourhan Khairy_H_1_D2O
www.pharma.cu.edu.eg dir-mau.fopcu@pharma.cu.edu.eg
BRUKER
7.5144 7.4954 7.4933 7.4104 7.3915 7.3893 7.3035 7.2894 7.2847 7.2706 7.2497 7.2123 7.1940 7.1914 7.1728 7.0577 6.9958 6.9774 6.9591 6.7203 6.5124 6.4921 4.0205 4.0179
2.5162 2.5119 2.5077
N
N
N
N
H
O
NH2
6
16 15 14 13 12 11 10 9 8 7 6 5 4 3 2 1 0 ppm
4.70 5.66 2.45 1.26 1.83 63.19 0.92

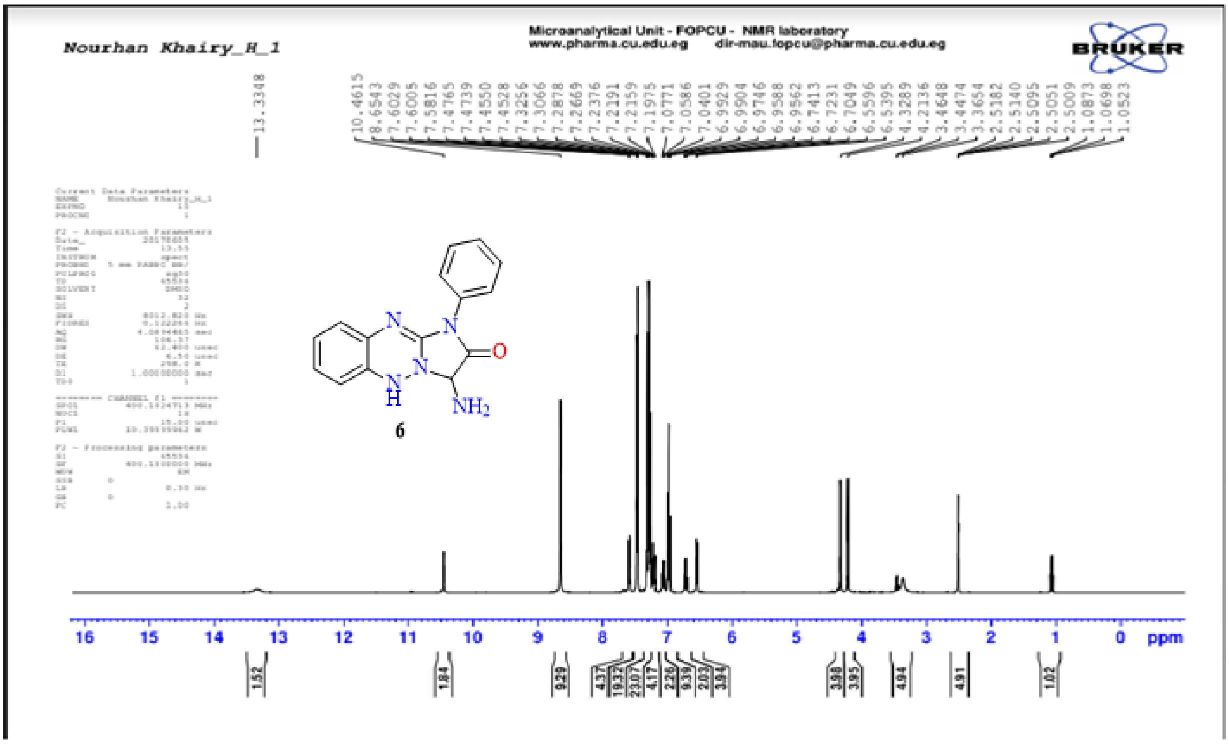
Nourhan Khairy_H_1
Microanalytical Unit - FOPCU - NMR laboratory
www.pharma.cu.edu.eg dir-mau.fopcu@pharma.cu.edu.eg
BRUKER
13.3348
10.4615 8.6543 7.6029 7.6005 7.5816 7.4765 7.4739 7.4550 7.4528 7.3256 7.3066 7.2878 7.2669 7.2376 7.2191 7.2159 7.1975 7.0771 7.0586 7.0401 6.9929 6.9904 6.9746 6.9588 6.9562 6.7413 6.7231 6.7049 6.5596 6.5395 4.3289 4.2136 3.4648 3.4474 3.3654 2.5382 2.5140 2.5095 2.5051 2.5009 1.0873 1.0698 1.0523
N
N
N
N
H
O
NH2
6
16 15 14 13 12 11 10 9 8 7 6 5 4 3 2 1 0 ppm
1.52 1.84 9.29 4.37 19.32 23.07 4.17 2.26 9.39 2.03 3.94 3.98 3.95 4.94 4.91 1.02

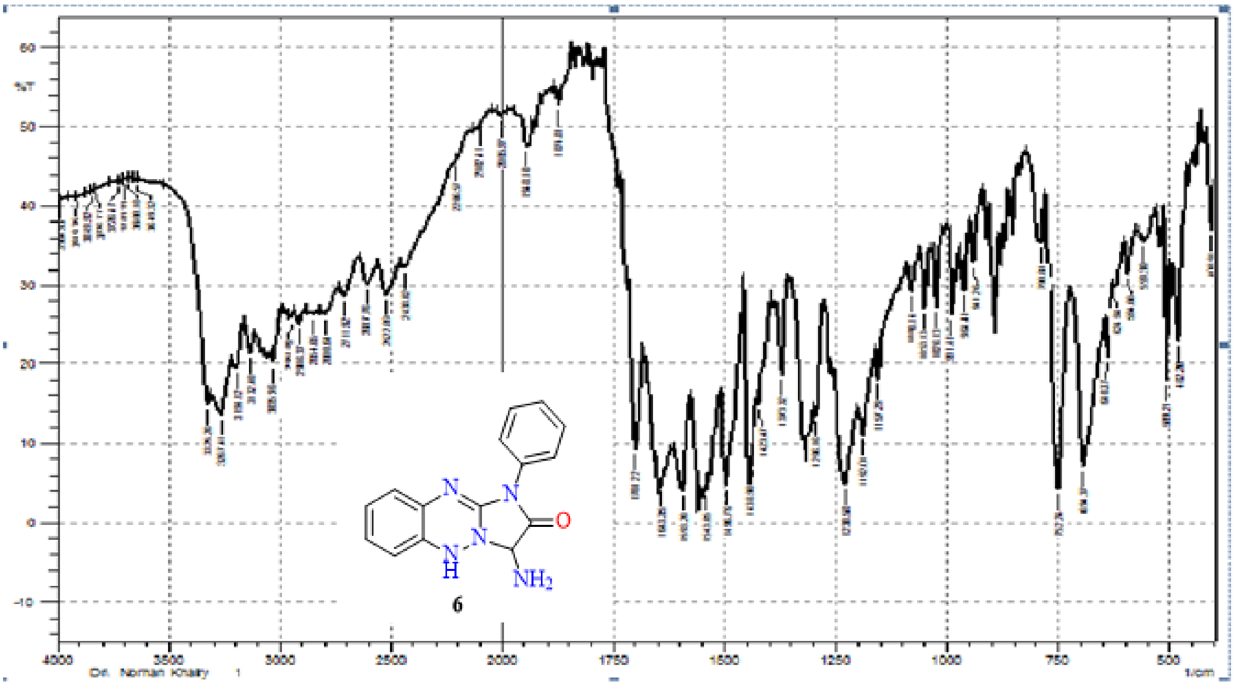
%T
60
50
40
30
20
10
0
-10
4000
3500
3000
2500
2000
1750
1500
1250
1000
750
500
1/cm
Dr. Noman Khairy 1
N
N
N
O
H
NH2
6

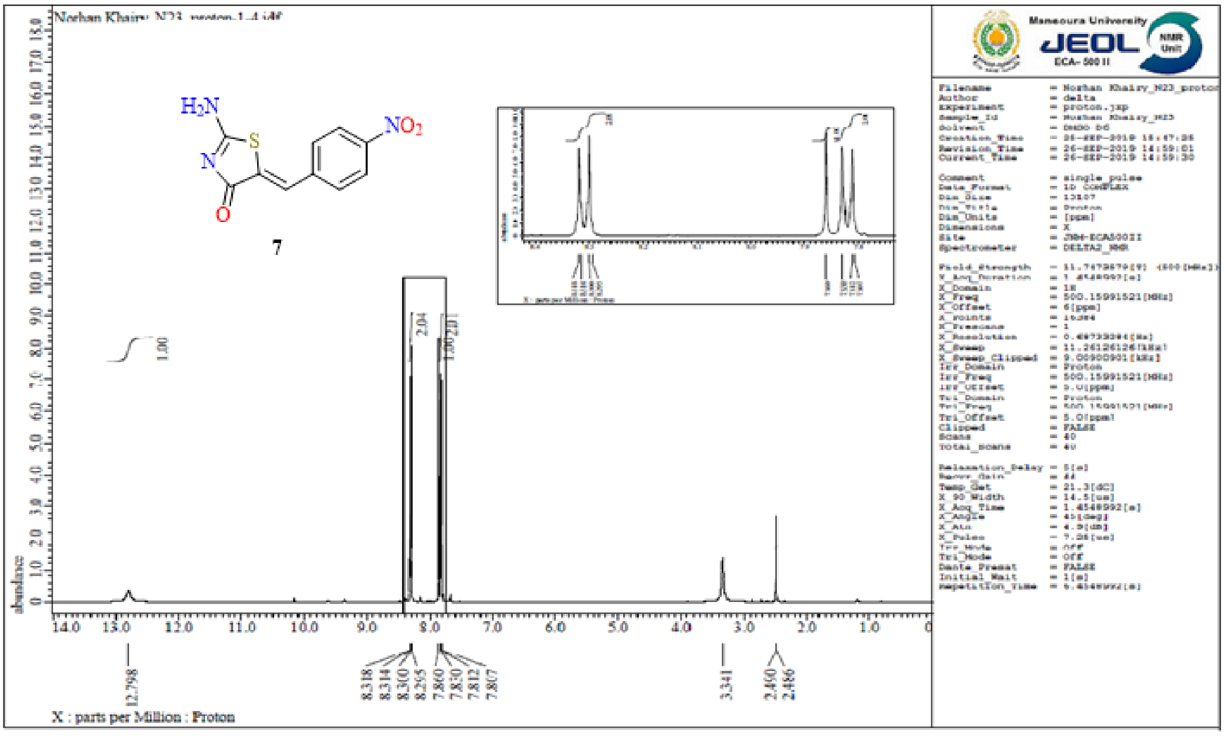

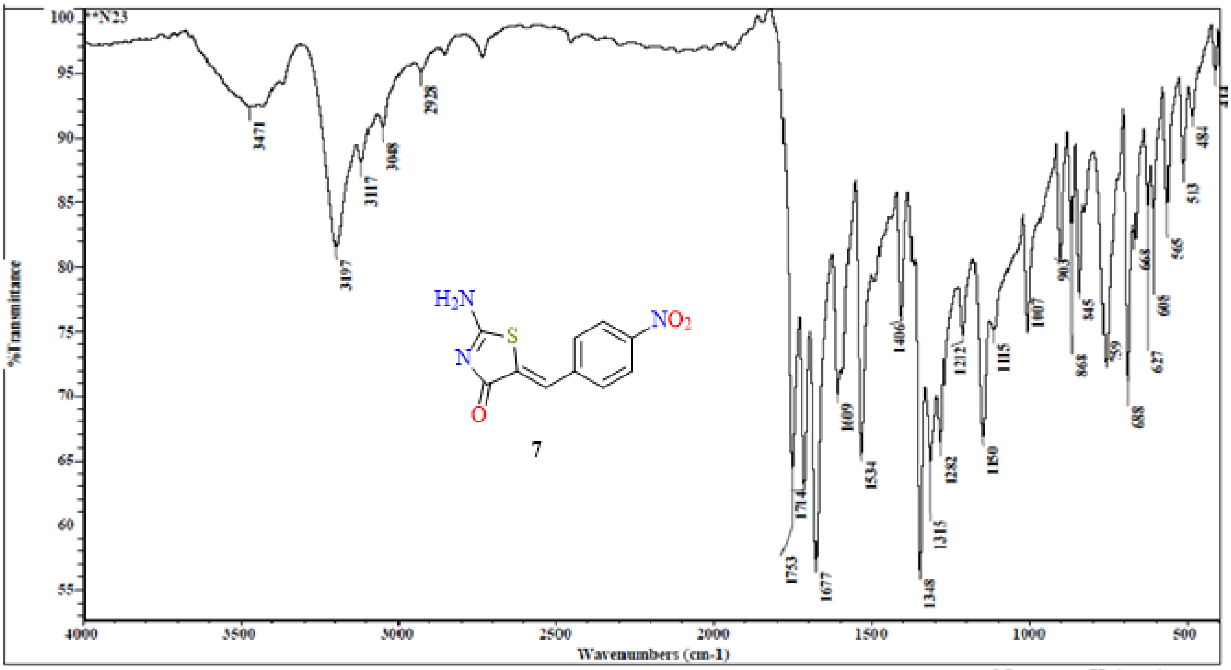

Number of sample scans: 32
Number of background scans: 32
Resolution: 4.000
Sample gain: 2.0
Optical velocity: 0.4747
Aperture: 80.00

ThermoFisher SCIENTIFIC
Thu Sep 12 09:37:07 2019 (GMT+02:00)

Mansoura University
Faculty of Science
Spectral Analysis Unit
unitofspectra@gmail.com

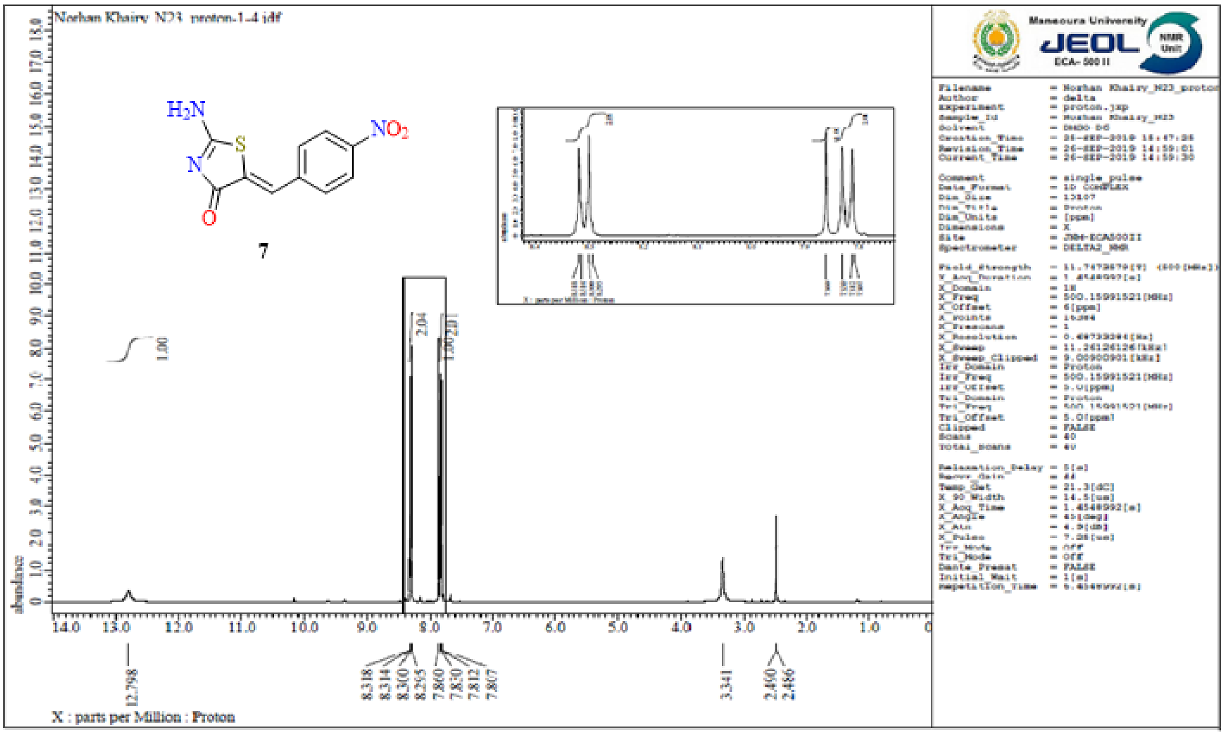

Norhan Khairy_N23_proton-1-4.jdf
H_2N
S
N
O
NO_2
7
Mansoura University
JEOL
NMR Unit
Filename = Norhan Khairy_N23_proton
Author = delta
Experiment = proton.jxp
Revision_Time = 26-SEP-2019 14:59:01
Comment = single_pulse
Data_Format = 1D COMPLEX
Dim_Units = [ppm]
Dimensions = X
Spectrometer = DELTA2_NMR
X_Domain = 1H
X_Freq = 500.15991521[MHz]
X_Offset = 6[ppm]
X_Sweep_Clipped = 9.00900901[kHz]
Irr_Domain = Proton
Irr_Freq = 500.15991521[MHz]
Tri_Offset = 5.0[ppm]
Clipped = FALSE
Scans = 40
Temp_Get = 21.3[dC]
X_90_Width = 14.5[us]
X_Acq_Time = 1.4548992[s]
Dante_Presat = FALSE
Initial_Wait = 1[s]
abundance
1.00
2.04
1.00
2.01
14.0 13.0 12.0 11.0 10.0 9.0 8.0 7.0 6.0 5.0 4.0 3.0 2.0 1.0 0
12.798
8.318
8.314
8.300
8.293
7.860
7.830
7.812
7.807
3.341
2.460
2.486
X : parts per Million : Proton

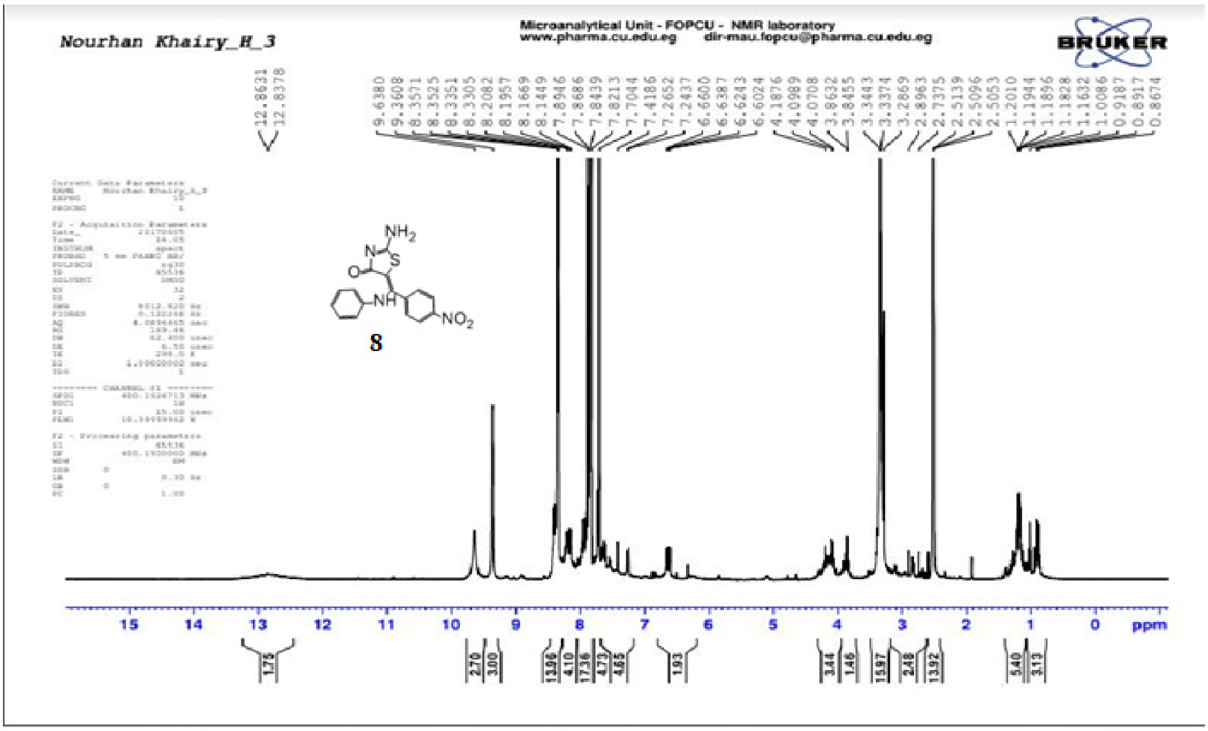
Nourhan Khairy_H_3
Microanalytical Unit - FOPCU - NMR laboratory
www.pharma.cu.edu.eg dir-mau.fopcu@pharma.cu.edu.eg
BRUKER
12.8631
12.8378
9.6380
9.3608
8.3571
8.3525
8.3351
8.3305
8.2082
8.1957
8.1669
8.1449
7.8946
7.8686
7.8439
7.8213
7.7044
7.4186
7.2652
7.2437
6.6600
6.6387
6.6243
6.6024
4.1876
4.0989
4.0708
3.8632
3.8455
3.3443
3.3374
3.2869
2.8963
2.7375
2.5139
2.5096
2.5053
1.2010
1.1944
1.1896
1.1828
1.1632
1.0086
0.9187
0.8917
0.8674
NH_2
NO_2
8
ppm
1.75
2.70
3.00
13.96
4.10
17.36
4.73
4.65
1.93
3.44
1.46
15.97
2.48
13.92
5.40
3.13

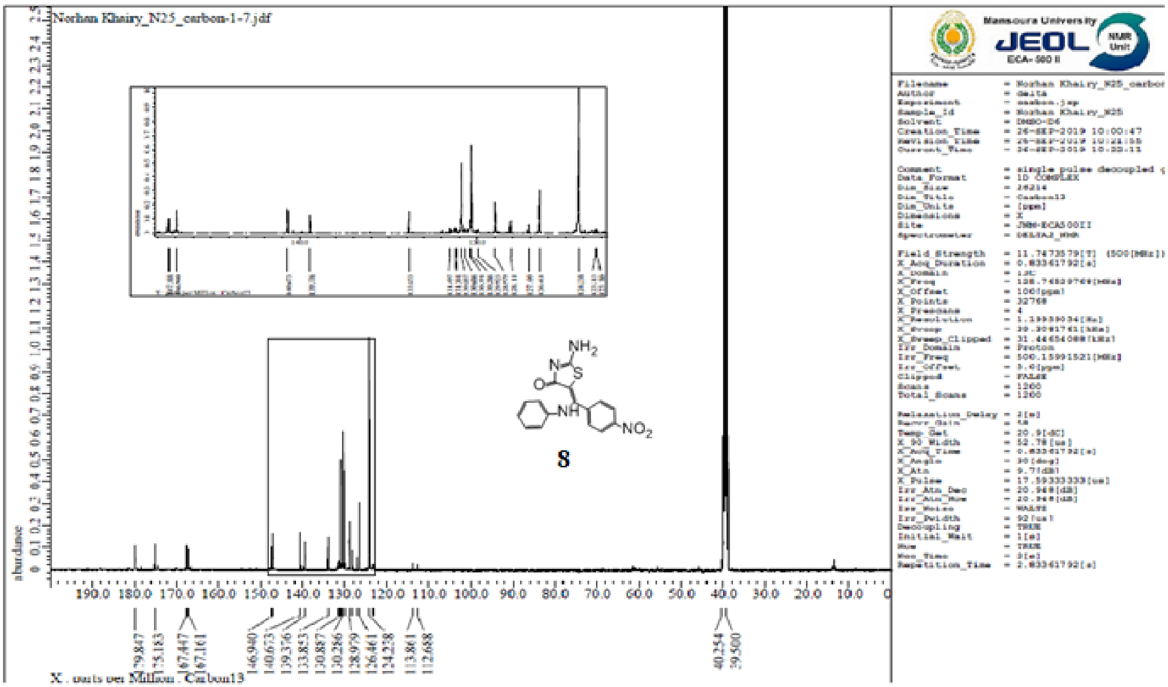
Norhan Khairy_N25_carbon-1-7.jdf
Mansoura University
JEOL
ECA- 500 II
NMR Unit
NH_2
NO_2
8
abundance
X : parts per Million : Carbon13
179.847
175.183
167.447
167.161
146.940
140.673
139.376
133.843
130.887
130.286
128.979
126.461
124.238
113.861
112.688
40.254
39.500

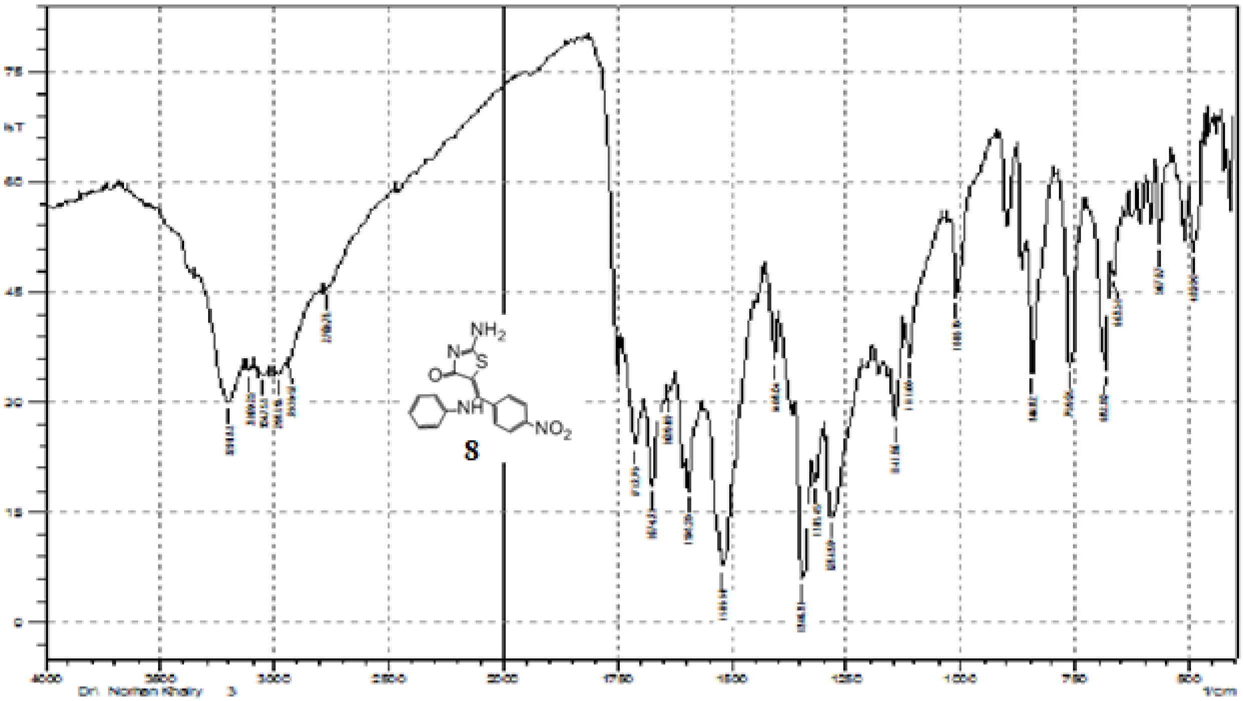

Nourhan Khairy_H_3_D2O

Microanalytical Unit - FOPCU - NMR laboratory
www.pharma.cu.edu.eg dir-mau.fopcu@pharma.cu.edu.eg

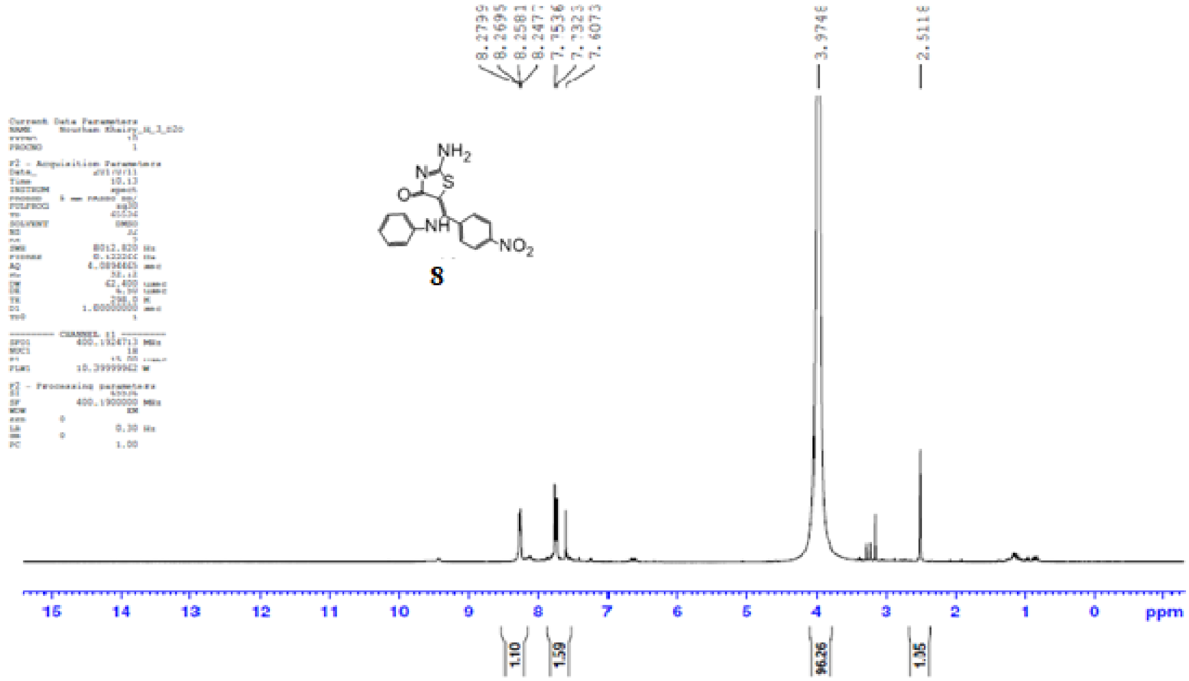

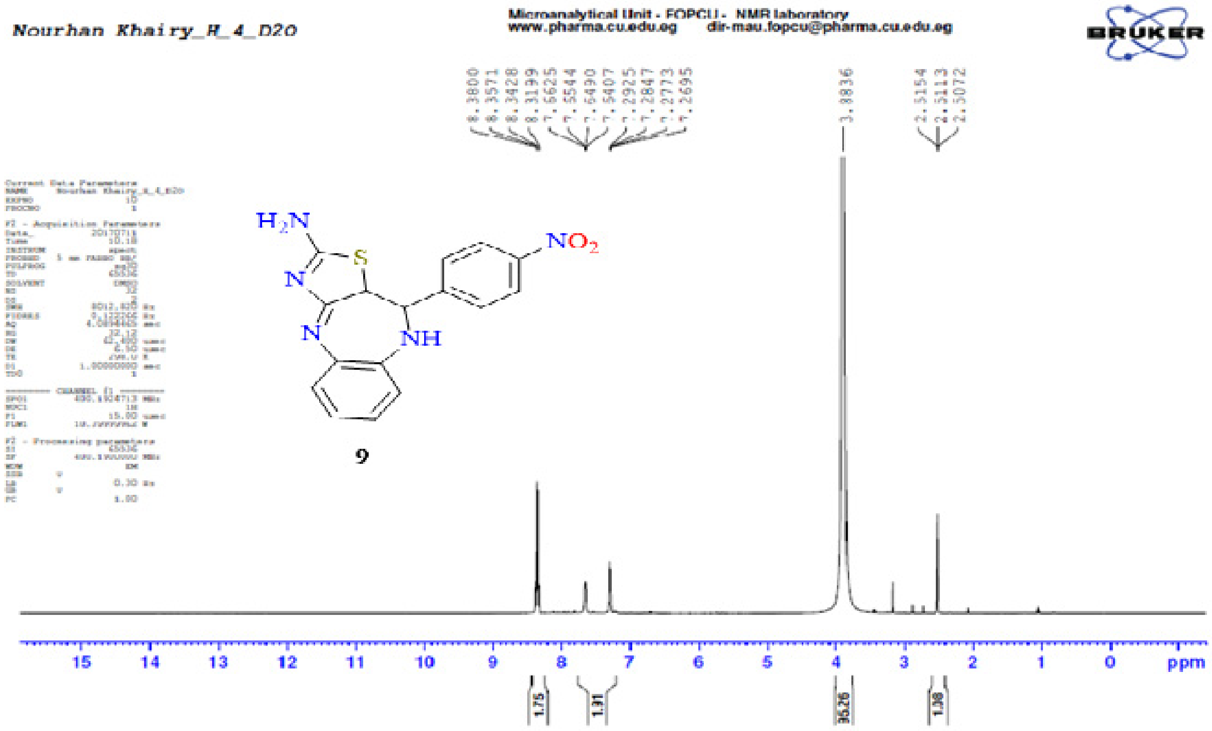

Nourhan Khairy_H_4_D2O
Microanalytical Unit - FOPCU - NMR laboratory
www.pharma.cu.edu.eg dir-mau.fopcu@pharma.cu.edu.eg
BRUKER
8.3800
8.3571
8.3428
8.3199
7.5625
7.5544
7.6490
7.5407
7.2925
7.2847
7.2773
7.2695
3.8836
2.5154
2.5113
2.5072
H2N
S
N
NO2
N
NH
9
15
14
13
12
11
10
9
8
7
6
5
4
3
2
1
0
ppm
1.75
1.31
35.26
1.08

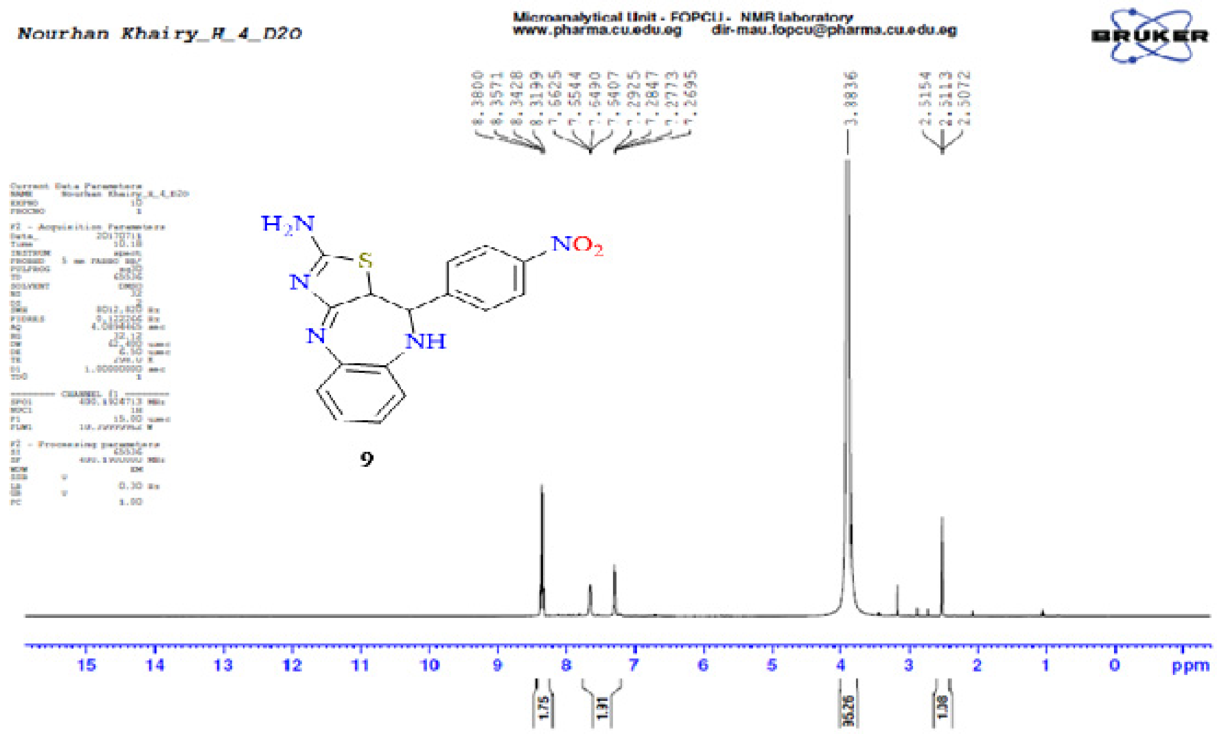
Nourhan Khairy_H_4_D2O
Microanalytical Unit - FOPCU - NMR laboratory
www.pharma.cu.edu.eg dir-mau.fopcu@pharma.cu.edu.eg
BRUKER
8.3800
8.3571
8.3428
8.3199
7.5625
7.5544
7.6490
7.5407
7.2925
7.2847
7.2773
7.2695
3.8836
2.5154
2.5113
2.5072
H2N
S
N
N
NH
NO2
9
15 14 13 12 11 10 9 8 7 6 5 4 3 2 1 0 ppm
1.75
1.31
35.26
1.08

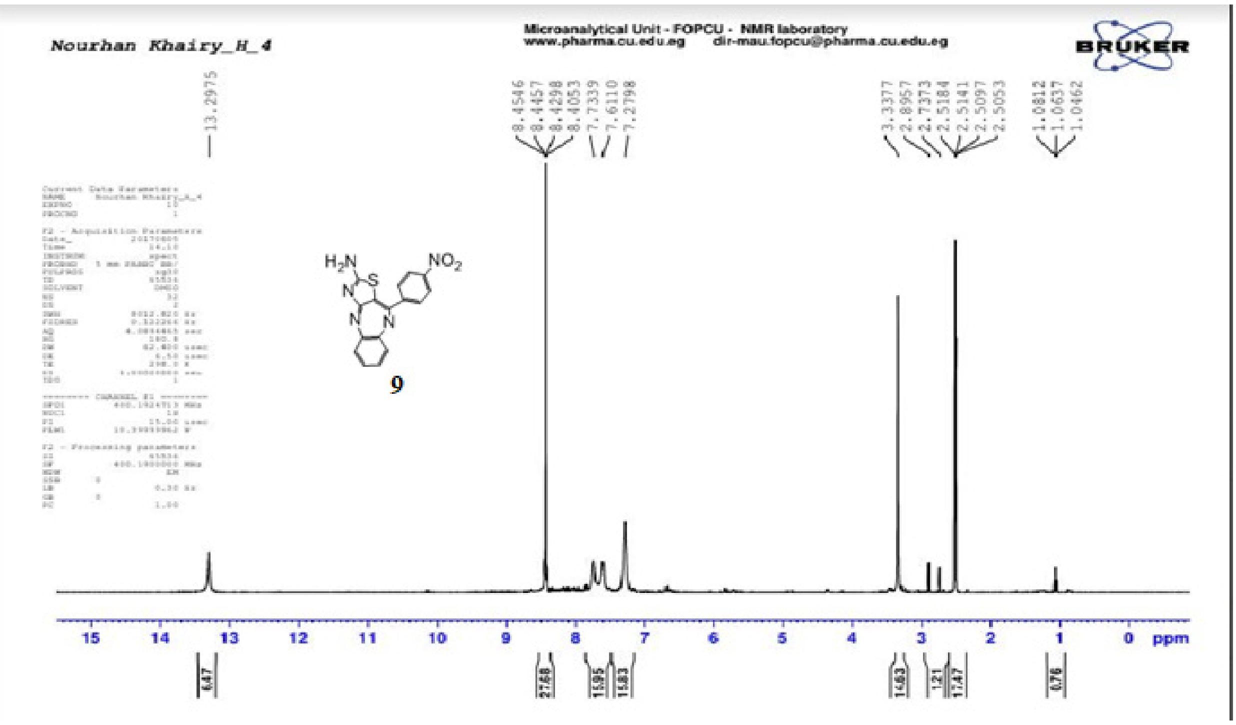
Nourhan Khairy_H_4
Microanalytical Unit - FOPCU - NMR laboratory
www.pharma.cu.edu.eg dir-mau.fopcu@pharma.cu.edu.eg
BRUKER
13.2975
8.4546
8.4457
8.4298
8.4053
7.7339
7.6110
7.2798
3.3377
2.8957
2.7373
2.5184
2.5141
2.5097
2.5053
1.0812
1.0637
1.0462
H2N
S
N
N
N
NO2
9
15 14 13 12 11 10 9 8 7 6 5 4 3 2 1 0 ppm
6.47
21.68
15.95
15.83
14.63
1.21
17.47
0.76

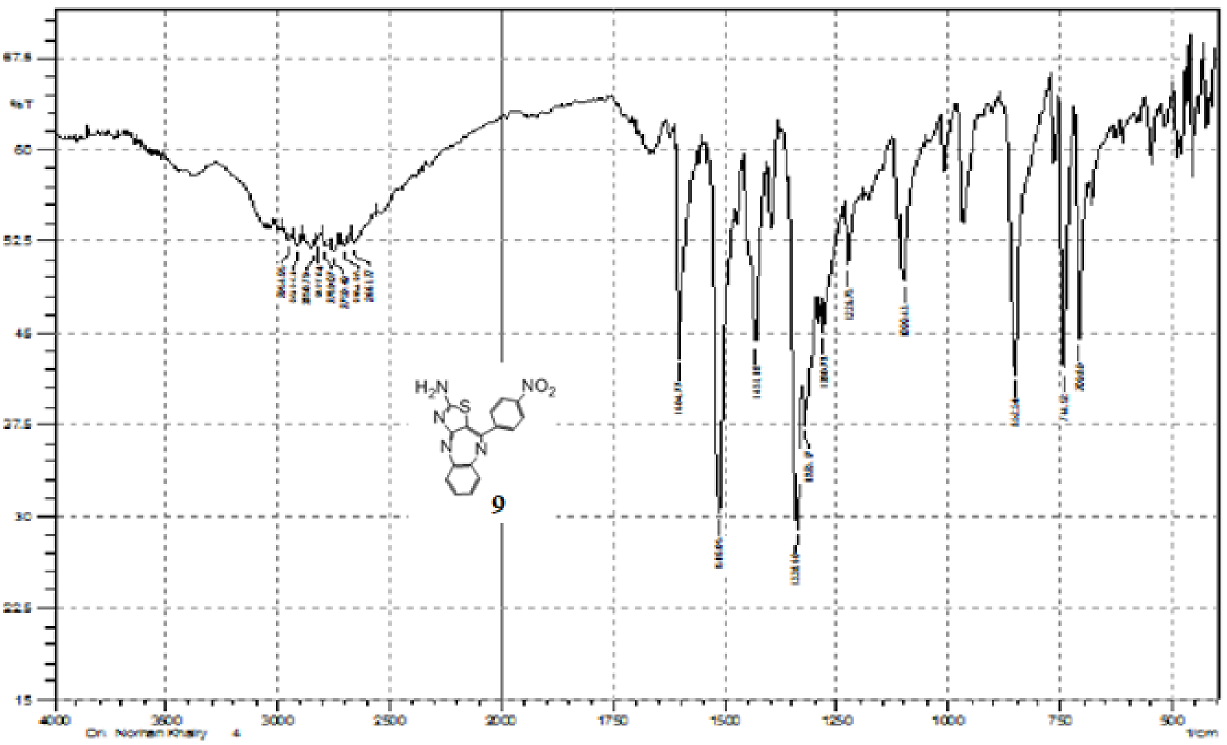

%T
cm-1
9

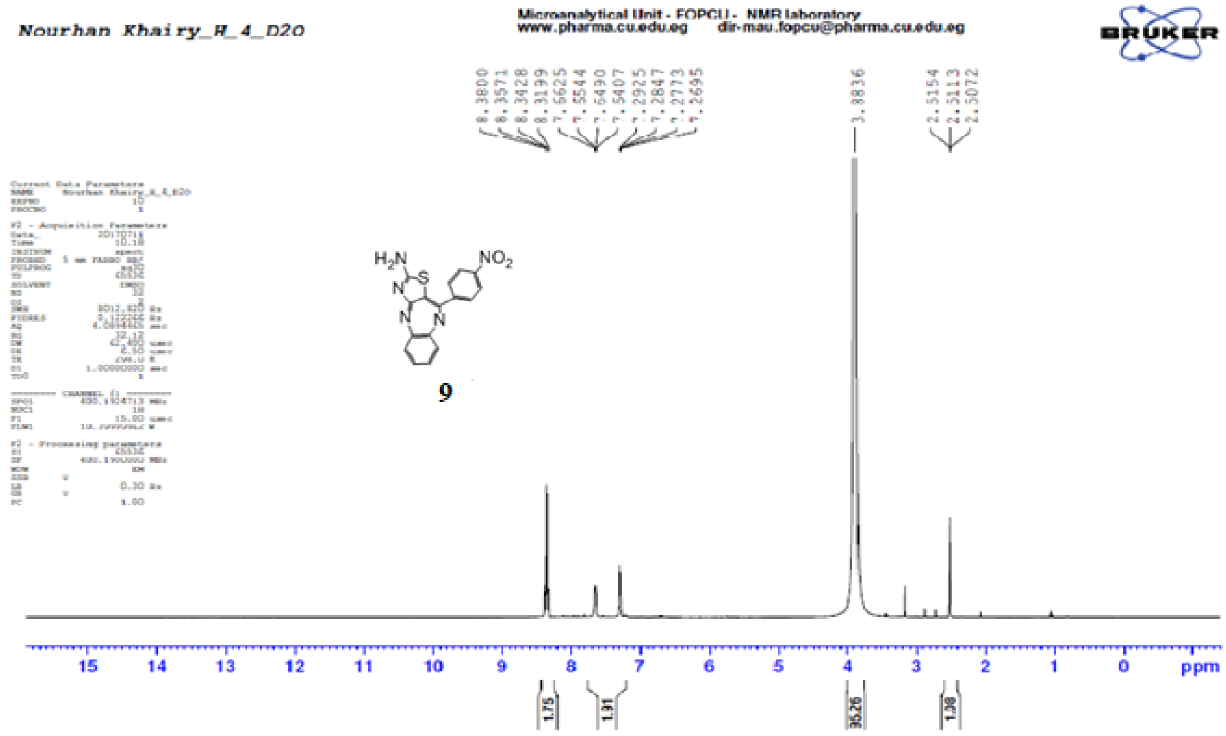

Nourhan Khairy_H_4_D2O
Microanalytical Unit - FOPCU - NMR laboratory
www.pharma.cu.edu.eg dir-mau.fopcu@pharma.cu.edu.eg
9
ppm

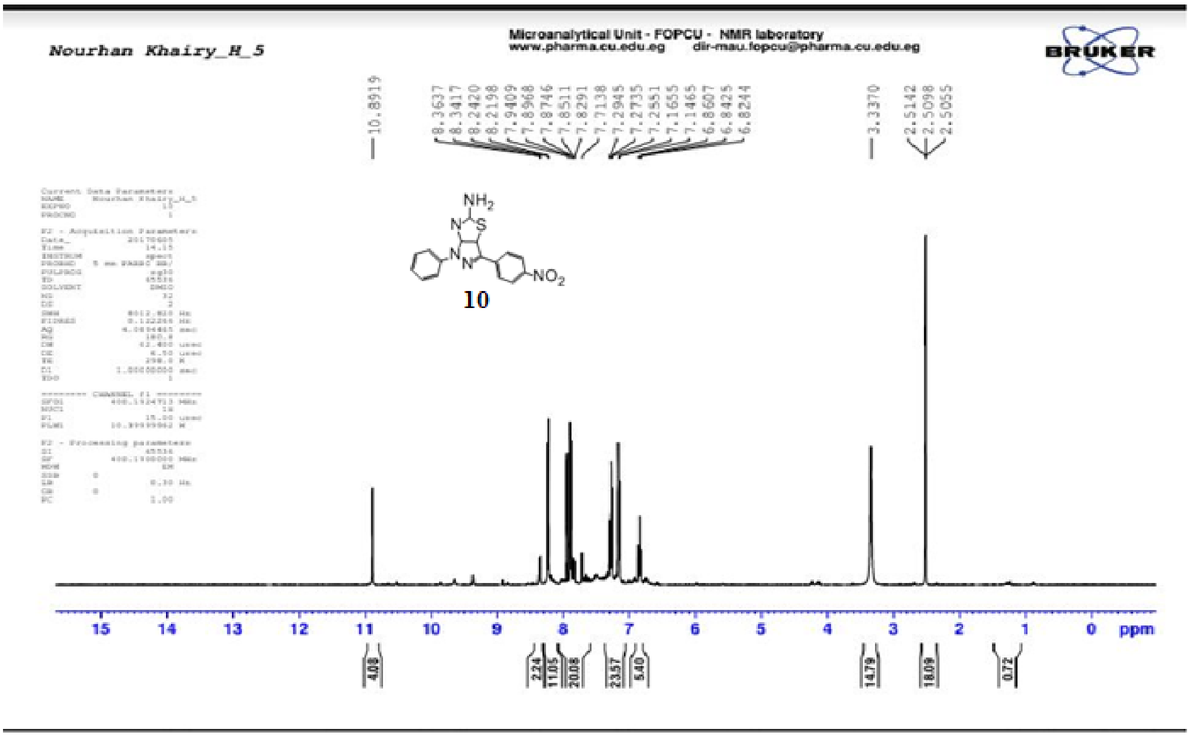
Nourhan Khairy_H_5
Microanalytical Unit - FOPCU - NMR laboratory
www.pharma.cu.edu.eg dir-mau.fopcu@pharma.cu.edu.eg
10.8919
8.3637
8.3417
8.2420
8.2198
7.9409
7.8968
7.8746
7.8511
7.8291
7.7138
7.2945
7.2735
7.2551
7.1655
7.1465
6.8607
6.8425
6.8244
3.3370
2.5142
2.5098
2.5055
NH2
N
S
N
N
NO2
10
15 14 13 12 11 10 9 8 7 6 5 4 3 2 1 0 ppm
4.08
2.24
11.05
20.08
23.57
5.40
14.79
18.09
0.72

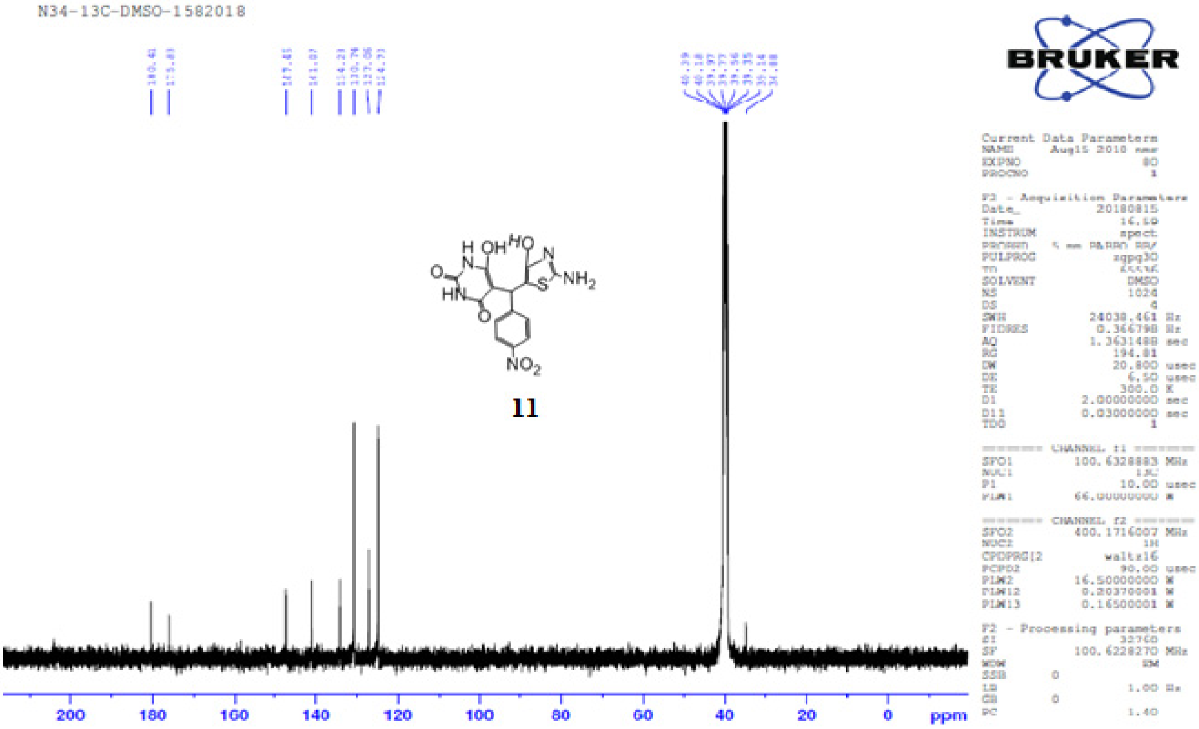
N34-13C-DMSO-1582018
180.41
175.83
147.45
141.07
134.23
130.76
127.06
124.73
BRUKER
11
200
180
160
140
120
100
80
60
40
20
0
ppm

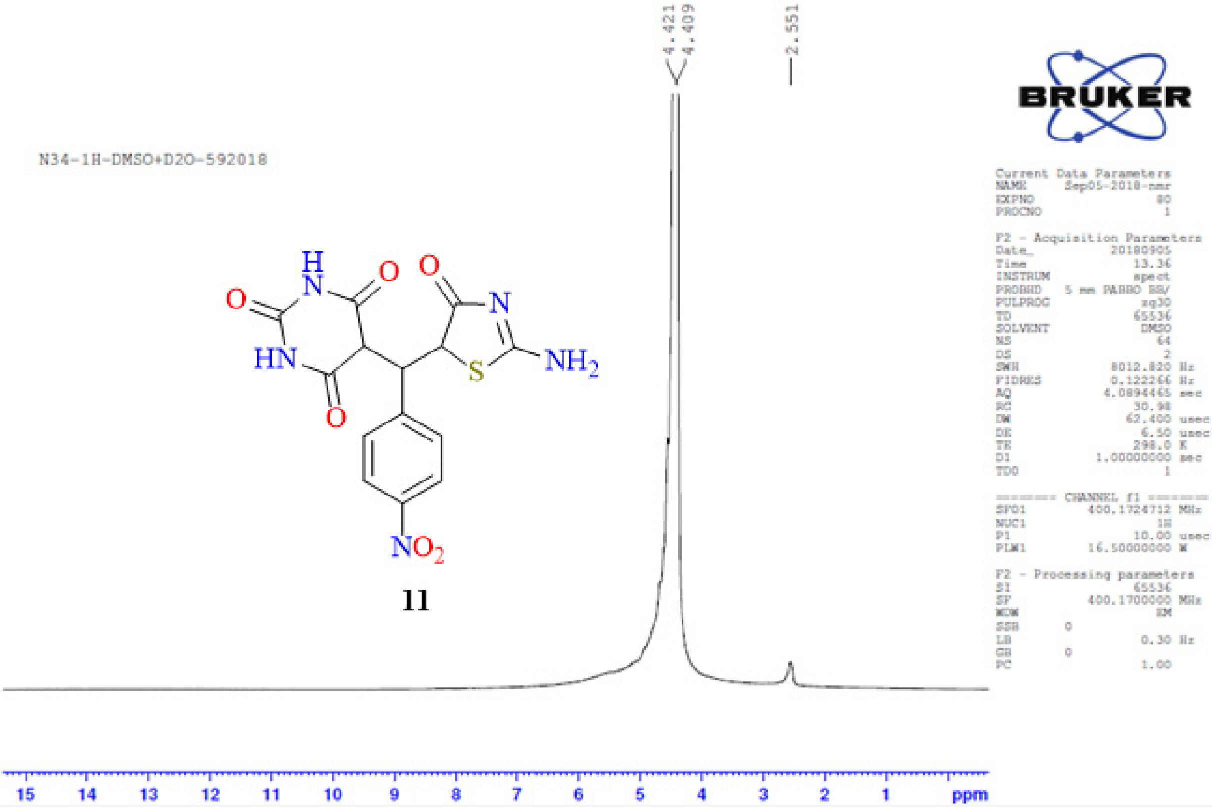
4.421
4.409
2.551
BRUKER
N34-1H-DMSO+D2O-592018
11
Current Data Parameters
NAME Sep05-2018-nmr
EXPNO 80
PROCNO 1
F2 - Acquisition Parameters
Date_ 20180905
Time 13.36
INSTRUM spect
PROBHD 5 mm PABBO BB/
PULPROG zg30
TD 65536
SOLVENT DMSO
NS 64
DS 2
SWH 8012.820 Hz
FIDRES 0.122266 Hz
AQ 4.0894465 sec
RG 30.98
DW 62.400 usec
DE 6.50 usec
TE 298.0 K
D1 1.00000000 sec
TD0 1
======== CHANNEL f1 ========
SFO1 400.1724712 MHz
NUC1 1H
P1 10.00 usec
PLW1 16.50000000 W
F2 - Processing parameters
SI 65536
SF 400.1700000 MHz
WDW EM
SSB 0
LB 0.30 Hz
GB 0
PC 1.00
15
14
13
12
11
10
9
8
7
6
5
4
3
2
1
ppm

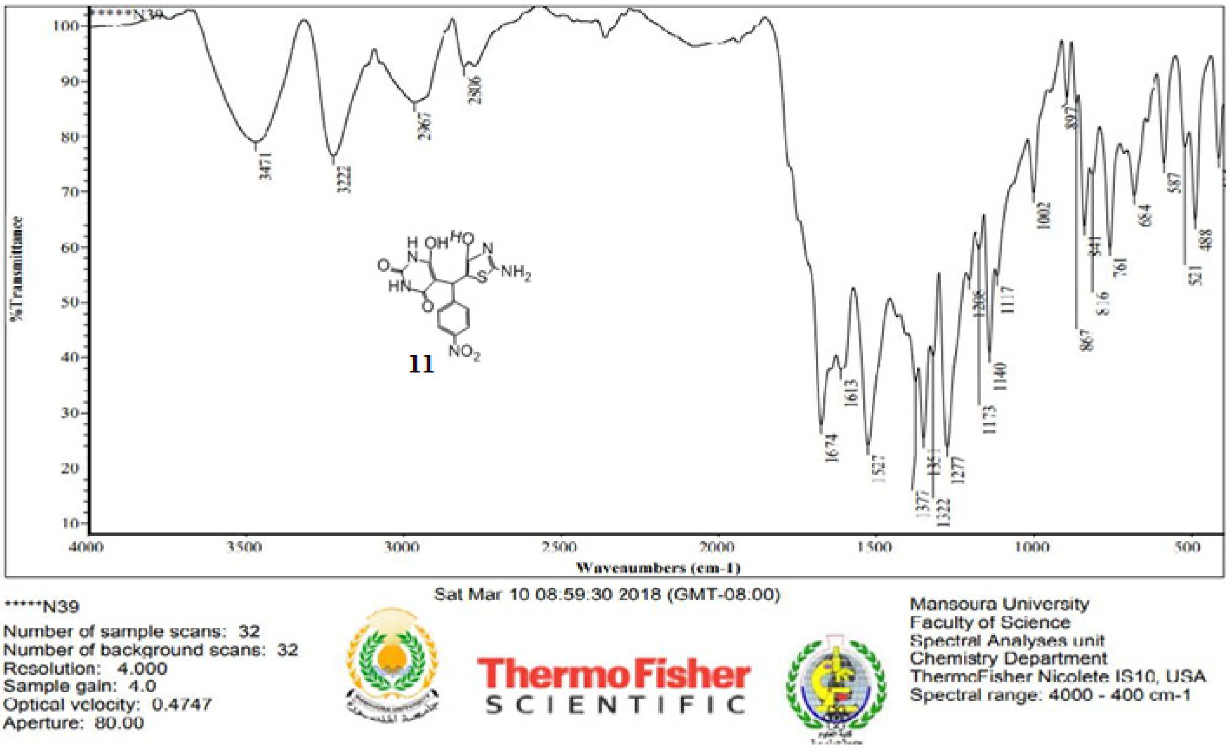
*****N39
%Transmittance
Wavenumbers (cm-1)
3471
3222
2967
2806
1674
1613
1527
1377
1322
1277
1206
1173
1140
1117
1002
897
867
841
816
761
684
587
521
488
11
Sat Mar 10 08:59:30 2018 (GMT-08:00)
*****N39
Number of sample scans: 32
Number of background scans: 32
Resolution: 4.000
Sample gain: 4.0
Optical velocity: 0.4747
Aperture: 80.00
Thermo Fisher SCIENTIFIC
Mansoura University
Faculty of Science
Spectral Analyses unit
Chemistry Department
ThermoFisher Nicolete IS10, USA
Spectral range: 4000 - 400 cm-1

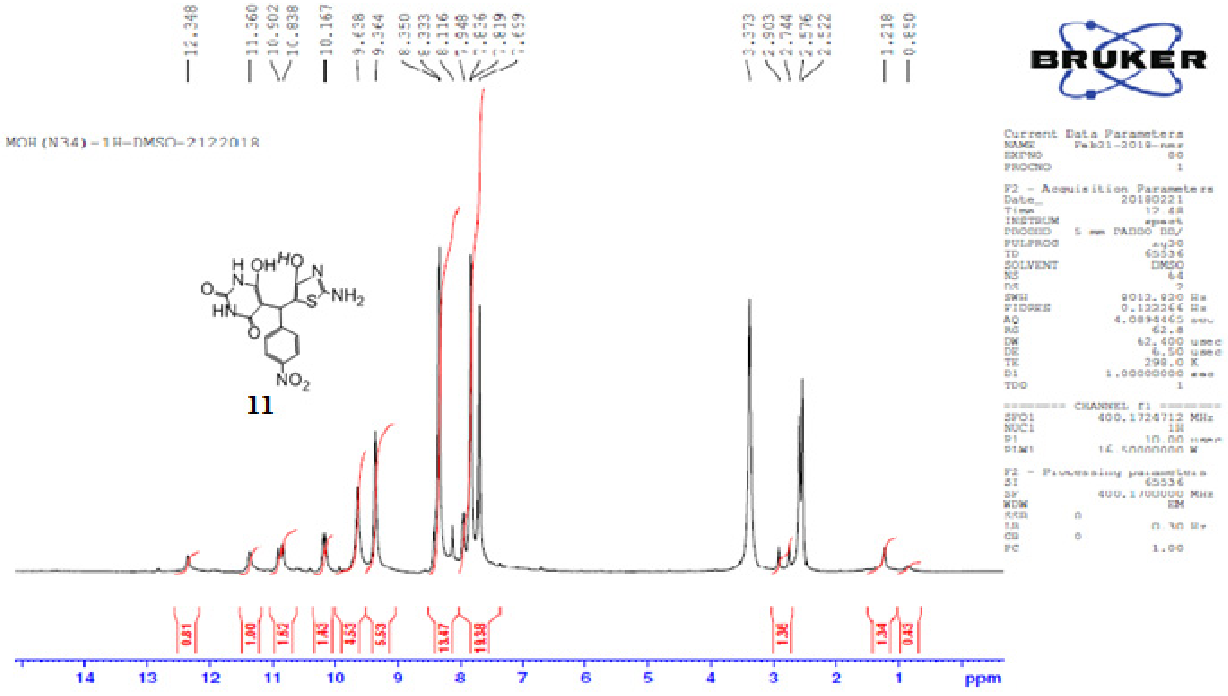
12.348
11.360
10.902
10.838
10.167
9.638
9.364
8.350
8.333
8.116
7.948
7.836
7.819
7.659
3.373
2.903
2.744
2.576
2.522
1.218
0.850
BRUKER
MOH(N34)-1H-DMSO-2122018
11
Current Data Parameters
F2 - Acquisition Parameters
CHANNEL f1
SFO1 400.1724712 MHz
F2 - Processing parameters
0.81
1.00
1.52
1.43
4.53
5.53
13.47
19.38
1.30
1.34
0.45
ppm
